# A revision of the Neotropical caddisfly genus *Leucotrichia* Mosely, 1934 (Hydroptilidae, Leucotrichiinae)

**DOI:** 10.3897/zookeys.499.8360

**Published:** 2015-04-23

**Authors:** Robin E. Thomson, Ralph W. Holzenthal

**Affiliations:** 1 Department of Entomology, University of Minnesota, 219 Hodson Hall, 1980 Folwell Avenue, St. Paul, Minnesota, 55108, U.S.A.

**Keywords:** Trichoptera, caddisflies, Hydroptilidae, *Leucotrichia*, new species, Neotropical

## Abstract

A revision of *Leucotrichia* (Trichoptera, Hydroptilidae) is provided, including a generic diagnosis, illustrations, a key, and descriptions of males. A total of 43 species are treated, 13 described as new: *Leucotrichia angelinae* **sp. n.** (Venezuela), *Leucotrichia denticulata* **sp. n.** (Mexico), *Leucotrichia dianeae* **sp. n** (Costa Rica), *Leucotrichia fulminea* **sp. n.** (Ecuador), *Leucotrichia hispida* **sp. n.** (Costa Rica), *Leucotrichia kateae* **sp. n.** (Venezuela), *Leucotrichia pectinata* **sp. n.** (Ecuador), *Leucotrichia procera* **sp. n.** (Brazil), *Leucotrichia repanda* **sp. n.** (Venezuela), *Leucotrichia rhomba* **sp. n.** (Costa Rica), *Leucotrichia riostoumae* **sp. n.** (Ecuador), *Leucotrichia sidneyi* **sp. n.** (Venezuela), and *Leucotrichia tapantia* **sp. n.** (Costa Rica).

## Introduction

The genus *Leucotrichia* Mosely, 1934 belongs to the family Hydroptilidae, the micro- or purse-case making caddisflies, and is the type genus for the subfamily Leucotrichiinae. The genus was originally established for a single species, *Leucotrichia melleopicta* Mosely, 1934, from Mexico (Tabasco). Additional species descriptions and distributions have been provided by numerous authors and the genus now contains a total of 29 extant species (Table 1). A single fossil species, *Leucotrichia adela* Wells & Wichard, 1989, is known from Dominican amber. The genus is endemic to the New World and its distribution includes most of the continental USA, Central America, and northern South America (Table 1) (Flint et al. 1999).

**Table 1.** *Leucotrichia* species, distributions, and species groups (Trichoptera, Hydroptilidae, Leucotrichiinae) († = extinct).

*Leucotrichia* species	Distribution	Species group
*Leucotrichia adela* Wells & Wichard, 1989 †	Dominican amber	*melleopicta*
*Leucotrichia alisensis* Rueda-Martín, 2011	Argentina	*melleopicta*
*Leucotrichia angelinae* sp. n.	Venezuela	*melleopicta*
*Leucotrichia ayura* Flint, 1991	Colombia	*melleopicta*
*Leucotrichia bicornuta* Thomson, 2012	Brazil	*melleopicta*
*Leucotrichia botosaneanui* Flint, 1996	Tobago, Trinidad	*melleopicta*
*Leucotrichia brasiliana* Sattler & Sykora, 1977	Brazil	*melleopicta*
*Leucotrichia brochophora* Flint, 1991	Colombia	*melleopicta*
*Leucotrichia chiriquiensis* Flint, 1970	Panama	*melleopicta*
*Leucotrichia denticulata* sp. n.	Mexico	*melleopicta*
*Leucotrichia dianeae* sp. n.	Costa Rica	*melleopicta*
*Leucotrichia dinamica* Bueno-Soria, 2010	Mexico	*melleopicta*
*Leucotrichia extraordinaria* Bueno-Soria, Santiago-Fragoso & Barba-Alvarez, 2001	Mexico	*melleopicta*
*Leucotrichia fairchildi* Flint, 1970	Colombia, Grenada, Panama, Tobago, Trinidad, Venezuela	*pictipes*
*Leucotrichia forrota* Oláh and Johanson, 2011	Peru, Ecuador	*melleopicta*
*Leucotrichia fulminea* sp. n.	Ecuador	*melleopicta*
*Leucotrichia gomezi* Flint, 1970	Dominican Republic	*melleopicta*
*Leucotrichia hispida* sp. n.	Costa Rica	*melleopicta*
*Leucotrichia imitator* Flint, 1970	Costa Rica, Guatemala, Mexico	*pictipes*
*Leucotrichia inflaticornis* Botosaneanu, 1993	Trinidad	*melleopicta*
*Leucotrichia inops* Flint, 1991	Colombia	*melleopicta*
*Leucotrichia interrupta* Flint, 1991	Colombia	*melleopicta*
*Leucotrichia kateae* sp. n.	Venezuela	*melleopicta*
*Leucotrichia laposka* Oláh & Johanson, 2011	Peru	*melleopicta*
*Leucotrichia lerma* Angrisano & Burgos, 2002	Argentina	*melleopicta*
*Leucotrichia limpia* Ross, 1944	Mexico, U.S.A.	*melleopicta*
*Leucotrichia melleopicta* Mosely, 1934	Mexico, Venezuela	*melleopicta*
*Leucotrichia mutica* Flint, 1991	Colombia	*melleopicta*
*Leucotrichia padera* Flint, 1991	Colombia	*melleopicta*
*Leucotrichia pectinata* sp. n.	Ecuador	*melleopicta*
*Leucotrichia pictipes* (Banks, 1911)	Mexico, U.S.A.	*pictipes*
*Leucotrichia procera* sp. n.	Brazil	*melleopicta*
*Leucotrichia repanda* sp. n.	Venezuela	*melleopicta*
*Leucotrichia rhomba* sp. n.	Costa Rica	*melleopicta*
*Leucotrichia riostoumae* sp. n.	Ecuador	*melleopicta*
*Leucotrichia sarita* Ross, 1944	Costa Rica, El Salvador, Guatemala, Mexico, U.S.A.	*pictipes*
*Leucotrichia sidneyi*, sp. n.	Venezuela	*melleopicta*
*Leucotrichia tapantia*, sp. n.	Costa Rica	*melleopicta*
*Leucotrichia termitiformis* Botosaneanu, 1993	Trinidad	*melleopicta*
*Leucotrichia tritoven* Flint, 1996	Tobago, Trinidad, Venezuela	*melleopicta*
*Leucotrichia tubifex* Flint, 1964	Dominican Republic, Haiti, Jamaica, Puerto Rico	*melleopicta*
*Leucotrichia viridis* Flint, 1967	El Salvador, Guatemala, Mexico, Panama	*melleopicta*
*Leucotrichia yungarum* Angrisano & Burgos, 2002	Argentina	*melleopicta*

In the original description, Mosely (1934) referred to the presence of ocelli, antennal joints in series of white and brown, a well-developed frenulum arising from the subcosta on the hind wing, legs with very dense setae, and a 1, 3, 4 tibial spur formula in the generic diagnosis. Structures of the maxillary and labial palpi were not described by Mosely. Features of the male genitalia and a forewing measurement of 2 mm were given in the species description of *Leucotrichia melleopicta*. Male genitalia features included the flattened terminal dorsal segment covering the genitalia, the deeply excised terminal ventral segment bearing setose margins, a spade-shaped “penis sheath”, and a slender process on the penultimate abdominal segment. Illustrations of the wing venation and the male genitalia were included in the description of the type species (Mosely 1934, figs 46–47).

As additional new species were described and placed in *Leucotrichia*, Flint (1970), in his generic revision, noted that there were certain characters that could be used to unite 2 species groups within the genus. Additional details regarding these species groups are discussed under “Species Relationships.” In his revision, Flint also provided several additional characters for generic-level diagnosis that had not been included in the original generic description by Mosely. Included were the pentagonal metascutellum, the heavily sclerotized trianguloid plate of tergite X, the subgenital plate extending ventrally from the ventral angles of tergite × to the base of the inferior appendages, the subapical spine of the inferior appendages, and the midlength complex of the phallus. Details regarding the wings were not included in Flint’s revision. In her review of all hydroptilid genera known at the time, Marshall (1979) stated that the wings of *Leucotrichia* were unmodified and made no mention of the well-developed frenulum originally mentioned by Mosely (1934).

Larvae have been associated with some species of *Leucotrichia*, but many are still unassociated (Flint et al. 1999). The known larvae are typical of the Leucotrichiinae in that large sclerites are present on 8 or 9 abdominal segments, only the pronotum is divided longitudinally, all legs are short and robust, and the submentum is divided (Flint 1970). The genus can be recognized, however, by the rugose or papillate head, single tarsal claw, and femora bearing spiniform dorsal setae (Marshall 1979). Wiggins (1996) noted the lack of lacunae on the dorsal abdominal sclerites and that the basal seta on each tarsal claw is not enlarged. The first 4 instars are strongly depressed, generally no more than 5 mm in length, and are free-living and found in fast flowing water on the upper surface of rocks, grazing on periphyton (McAuliffe 1982, Wiggins 1996). During the fifth and final retreat dwelling instar, the larva constructs a fixed, silken shelter within which it lives and continues to feed, extending its forelegs from openings in either end of the retreat (Flint 1991, Wiggins 1996). During this time, the larva displays extreme lateral distention of abdominal segments V–VII (Wiggins 1996). The retreats are generally flattened, oval cases no longer than 5.5 mm, slightly domed, and tightly attached to the surface of a rock or boulder. (Marshall 1979, Wiggins 1996).

Like other members of Hydroptilidae, *Leucotrichia* adults are minute, although by reaching a length of 5 mm they represent some of the larger species in the family. Many species bear patches of bright green setae on their forewings, which may fade to a lighter green or yellow color in older, pinned specimens. Adults occasionally come to light at night, but usually are collected from riparian foliage during the day with a net (Flint et al. 1999). Females have been associated for some species, but many remain unassociated.

The most recent works to assess *Leucotrichia* include those done by Flint (1970), Marshall (1979), and Oláh and Johanson (2011). In Flint’s (1970) revision, he proposed the subfamily Leucotrichiinae for *Leucotrichia* and its related genera: *Abtrichia*, *Acostatrichia*, *Alisotrichia*, *Anchitrichia*, *Betrichia*, *Costatrichia*, *Peltopsyche*, and *Zumatrichia*. He also stated that the subfamily could be divided into 2 definite units, 1 consisting of *Alisotrichia* and the other consisting of all the other genera. Aside from this division, Flint made no mention of a more definite placement of *Leucotrichia* in regards to generic relationships within the subfamily. At the time of Marshall’s (1979) review, only 10 species of *Leucotrichia* were described; she also refrained from commenting on possible relationships of *Leucotrichia*. Since then, many new species have been described and added to the genus, including 2 described by Oláh and Johanson (2011). In this same work, Oláh and Johanson also divided Leucotrichiinae into 2 generic clusters: the *Leucotrichia* genus cluster, which included *Abtrichia*, *Acostatrichia*, *Anchitrichia*, *Ascotrichia*, *Betrichia*, *Ceratotrichia*, *Costatrichia*, *Leucotrichia*, and *Zumatrichia*, and the *Celaenotrichia* genus cluster, which included *Alisotrichia*, *Byrsopteryx*, *Celaenotrichia*, *Cerasmatrichia*, *Mejicanotrichia*, and *Scelobotrichia*. The *Leucotrichia* genus cluster can be distinguished from the *Celaenotrichia* genus cluster by members that share a modified spur formula and the typical leucotrichiine phallic median complex originally described in 1970 by Flint (Oláh and Johanson 2011). Table 2 summarizes the features briefly outlined by Oláh and Johanson (2011) that can be used to distinguish the genus *Leucotrichia* from other members of the *Leucotrichia* genus cluster.

**Table 2.** Character states of genera in the *Leucotrichia* genus cluster, modified from Oláh and Johanson (2011). “Modifications” of the head, antennae, and wings, as compared to the “unmodified” conditions of these structures, are discussed in the Generic Description subsection.

Genus	Number of ocelli	Head	Antennae	Wing	Spine on sternum VIII	Process on segment IX	Inferior appendages
*Abtrichia*	2	m	m	m	-	+	s
*Acostatrichia*	3	u	u	m/u	-/+	-/+	s
*Anchitrichia*	2	u	u	u	+	-	s
*Ascotrichia*	2	m	u	u	+	+	f
*Betrichia*	2–3	m/u	m/u	u	-/+	-	f
*Ceratotrichia*	2	m	m	u	+	-	s
*Costatrichia*	3	u	m/u	m/u	-/+	-/+	s
*Leucotrichia*	2–3	m/u	m/u	u	-	-	s/f
*Zumatrichia*	2	m	m	u	-/+	+	s/f

m = modified, u = unmodified, - = absent, + = present, s = separate, f = fused

**Figure 1.**
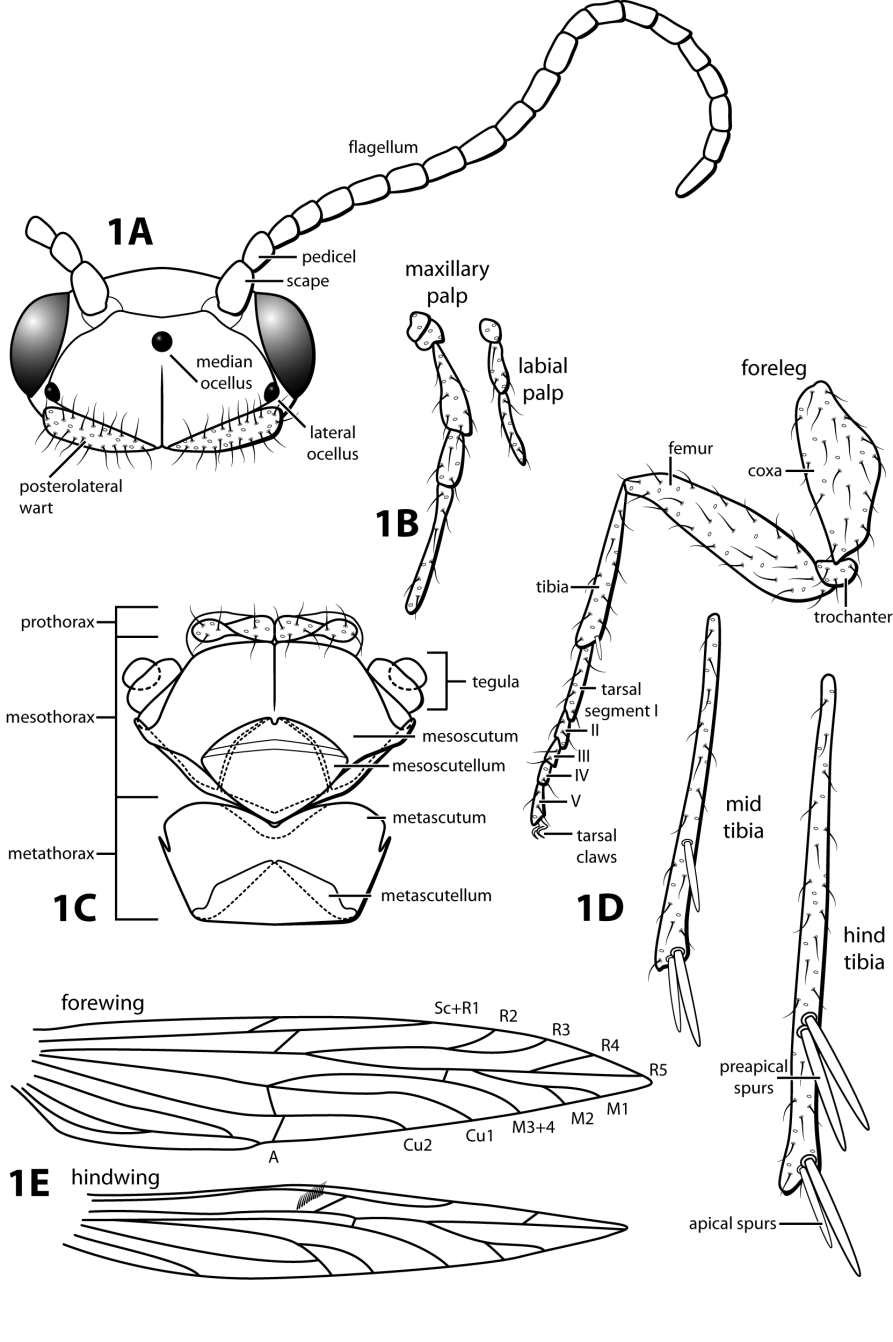
*Leucotrichia melleopicta* Mosely, 1934 (holotype, NHM). **A** head and antennae, dorsal **B** palps **C** thorax, dorsal **D** legs and spur formula (1.3.4) **E** wings.

**Figure 2.**
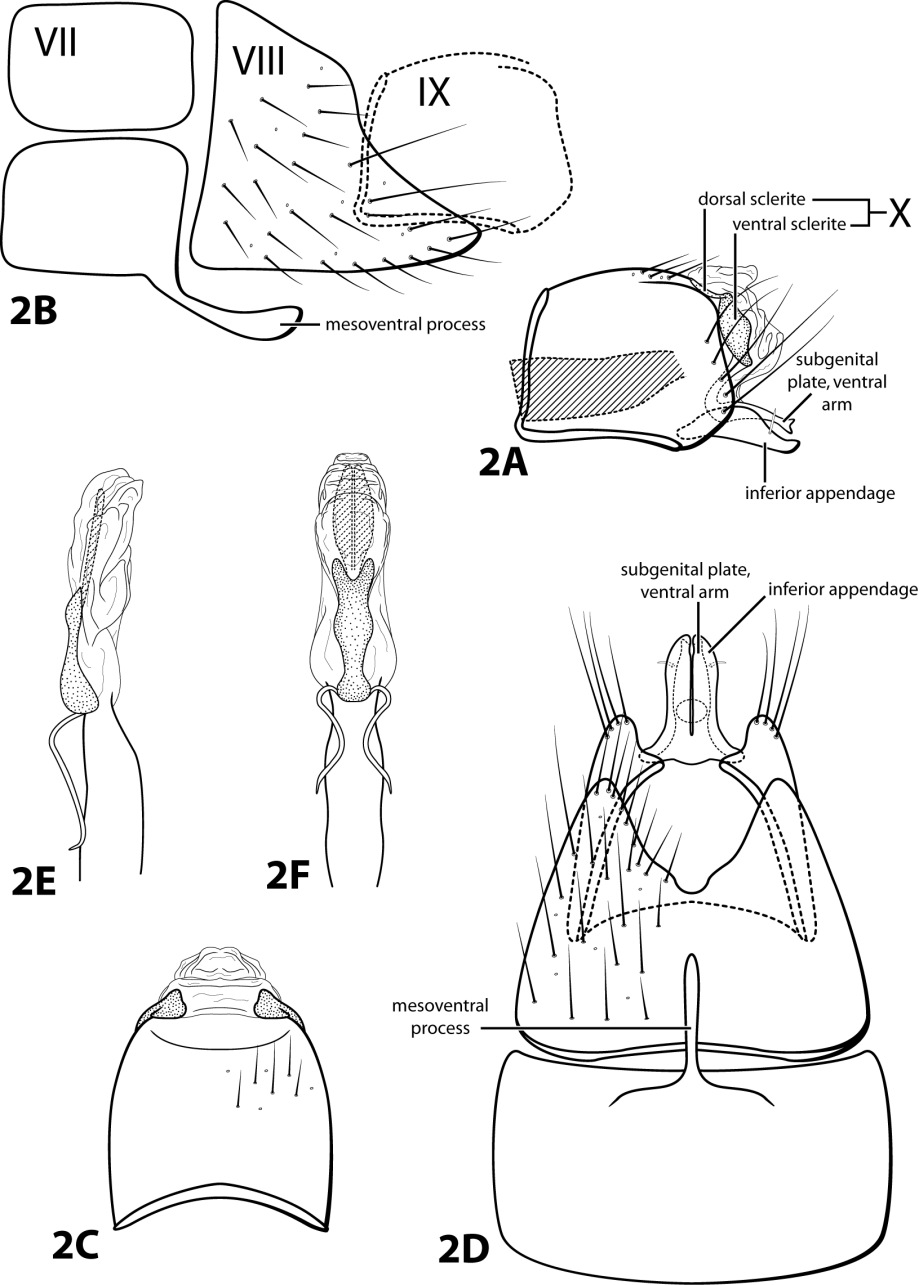
*Leucotrichia melleopicta* Mosely, 1934 (holotype, NHM). Male genitalia: **A** segments IX–X, lateral (base of phallus crosshatched) **B** segments VII–VIII and segment IX margin, lateral **C** segments IX–X, dorsal **D** segments VII–IX, ventral **E** phallus, lateral **F** phallus, dorsal.

In addition to the characters listed in Table 2, a few previously mentioned characters are proposed that can be used to distinguish members of the genus. The structure of the subgenital plate mentioned by Flint (1970), with the presence of the ventral arm, and occasionally the dorsal arm, is a synapomorphy of *Leucotrichia* (Figs 2A, 10A). Additionally, the very prominent row of elongate setae along the posterolateral margin of segment IX is proposed as unique to *Leucotrichia* (Fig. 2A). The genus *Leucotrichia* is monophyletic based on these characters, which can be used to separate them from other leucotrichiine genera. The well-developed frenulum of the hind wing, first mentioned by Mosely (1934) in the original description, combined with the absence of any wing modifications, such as a pouch or bulla, although not an autapomorphky of the genus, is helpful when distinguishing *Leucotrichia* from other genera (Fig. 1E).

The genera traditionally recognized as members of the subfamily have historically been difficult to separate (Flint 1970, Marshall 1979). Further work is needed to delimit the genera and properly assess their taxonomic and phylogenetic status. This review of *Leucotrichia* was undertaken as a first attempt to define the boundaries of the leucotrichiine genera and to improve our knowledge of the genus by defining characters that separate *Leucotrichia* from other leucotrichiine genera (discussed above) and by establishing a standardized terminology of the male genitalic structures among species of the genus. Additionally, we re-describe and illustrate the 30 known *Leucotrichia* species and describe 13 new species, bringing the total number of species to 43. Finally, a key is provided to the males of *Leucotrichia*.

## Materials and methods

### Specimen preparation and observation

To observe structural features of the male genitalia, soft tissues were cleared following procedures explained in detail by Blahnik et al. (2007). Abdomens, including genitalia, were removed from specimens using microscissors and placed individually in carefully labeled Pyrex® test tubes (10 × 75 mm), each containing 2–3 milliliters of 85% lactic acid. Test tubes were then heated in a Fischer Scientific dry bath incubator at approximately 120 °C for 30–35 minutes. At the end of this time, abdomens were carefully removed from the test tubes and rinsed in ethanol to gently flush away any remaining lactic acid. For some specimens, the head was also removed and cleared to more easily observe modifications and eversible structures obscured by dense setae. For specimen examination, cleared genitalia were placed in a standard glass microscope depression slide (1.5 cm diameter × 3 mm deep well) with glycerin and glass microbeads (average diameter 0.5 mm). The glass microbeads held the genitalia in place and allowed structures to be viewed in precise lateral, dorsal, and ventral positions. Genitalia were examined with an Olympus BX41 compound microscope at 250–500 × magnification. Due to their small size and reduced venation, the wings of Hydroptilidae do not provide reliable taxonomic characters (Marshall 1979, Schmid 1998). For these reasons, forewing length only has been provided in species descriptions.

### Illustrations and descriptions

Structures were traced in pencil with the use of an Olympus drawing attachment (model U-DA) mounted on the microscope. Pencil sketches were then scanned (Fujitsu ScanSnap S1500M scanner), edited in Adobe Photoshop (v. 9.0.2, Adobe Systems Inc.), and used as a template in Adobe Illustrator (v. 13.0.2, Adobe Systems Inc.) to be digitally inked. Electronic “drawing” was completed with the aid of a graphics tablet (Bamboo Splash, Wacom Company, Limited). Species descriptions were constructed using the program DELTA (Dallwitz et al. 1999 onwards), which uses a species × character state data matrix to produce natural-language descriptions and promote consistency in descriptive taxonomy.

Description of female specimens has been deferred for several reasons. Since female specimens were available for less than half of the species addressed in this study (18 of 43), comprehensive female descriptions for each species were not possible. Of the females examined, no noticeable or informative differences were observed. While some female specimens were collected from the same locality and date as males, there is still some uncertainty of association. For these reasons, species descriptions reflect observation of male specimens only. Females of some species were included in “Material examined” for the purpose of establishing a record of occurrence and because presumptive association may prove useful for future studies.

### Morphological terminology

Morphological terminology used for male genitalia was adapted from Marshall (1979) and Flint (1970). For simplicity, paired structures are discussed in the singular. Terminology for specific structures is indicated in Figures 1–2, 7, and 9. The wing venation terminology of Figure 1E follows the Comstock-Needham system as interpreted by Ross (1956) and Marshall (1979).

### Depositories

Types and material examined for this study are deposited at the Colleción Nacional de Insectos, Universidad Nacional Autónoma de México, Mexico City, Mexico (CNIN); Coleção Entomológica Professor José Alfredo Pinheiro Dutra, Departamento de Zoologia, Universidade Federal do Rio de Janeiro, Rio de Janeiro, Brazil (DZRJ); Instituto Miguel Lillo, Tucamán, Argentina (IML); Illinois Natural History Survey, Champaign, Illinois, USA (INHS); Museum of Comparative Zoology, Harvard University, Cambridge, Massachusetts, USA (MCZ); Museo del Instituto de Zoología Agrícola, Universidad Central de Venezuela, Maracay, Venezuela (MIZA); Museu de Zoologia, Universidade de São Paolo, São Paolo, Brazil (MZUSP); Museo de Zoología, Universidad Tecnológica Indoamérica, Quito, Ecuador (MZUTI); Natural History Museum, London, United Kingdom (NHM); Swedish Museum of Natural History, Stockholm, Sweden (NHRS); National Museum of Natural History, Washington, D.C., USA (NMNH); Dr. Janós Oláh Private Collection, presently under national protection of the Hungarian Natural History Museum, Hungary (OPC); University of Minnesota Insect Collection, Saint Paul, Minnesota, USA (UMSP); and Zoölogisch Museum Universiteit van Amsterdam, The Netherlands (ZMUA).

Specimen management followed the procedures outlined by Holzenthal and Anderson (2004). Each pinned specimen examined during the study was affixed with a barcode label (4 mil polyester, 8 × 14 mm, code 49) bearing a unique alphanumeric sequence beginning with the prefix UMSP. Specimens in alcohol were given a single barcode label to represent all those in a single vial. The prefix is not meant to imply ownership by the University of Minnesota Insect Collection (UMSP), but only to indicate that the specimen was databased at that collection and to provide unique identification of specimens. Specimens that had already been affixed with a label bearing a unique identification number from their home depository were not given an additional UMSP barcode. Specimen-level taxonomic, locality, collection, and other information are stored in the University of Minnesota Insect Collection Biota Trichoptera Database using the open-source software Biota v. 3.0 (Colwell 2012).

## Systematics

### Generic description

#### Genus Leucotrichia

Taxon classificationAnimaliaTrichopteraHydroptilidae

Mosely, 1934

Leucotrichia Mosely, 1934: 157 [Type species: *Leucotrichia melleopicta* Mosely, 1934, original designation]. Flint 1970: 3 [key, revision]. — Marshall 1979: 178 [review of the genus]. — Oláh and Johanson 2011: 152 [discussion of *Leucotrichia* genus cluster].

##### Description.

**Figure 3.**
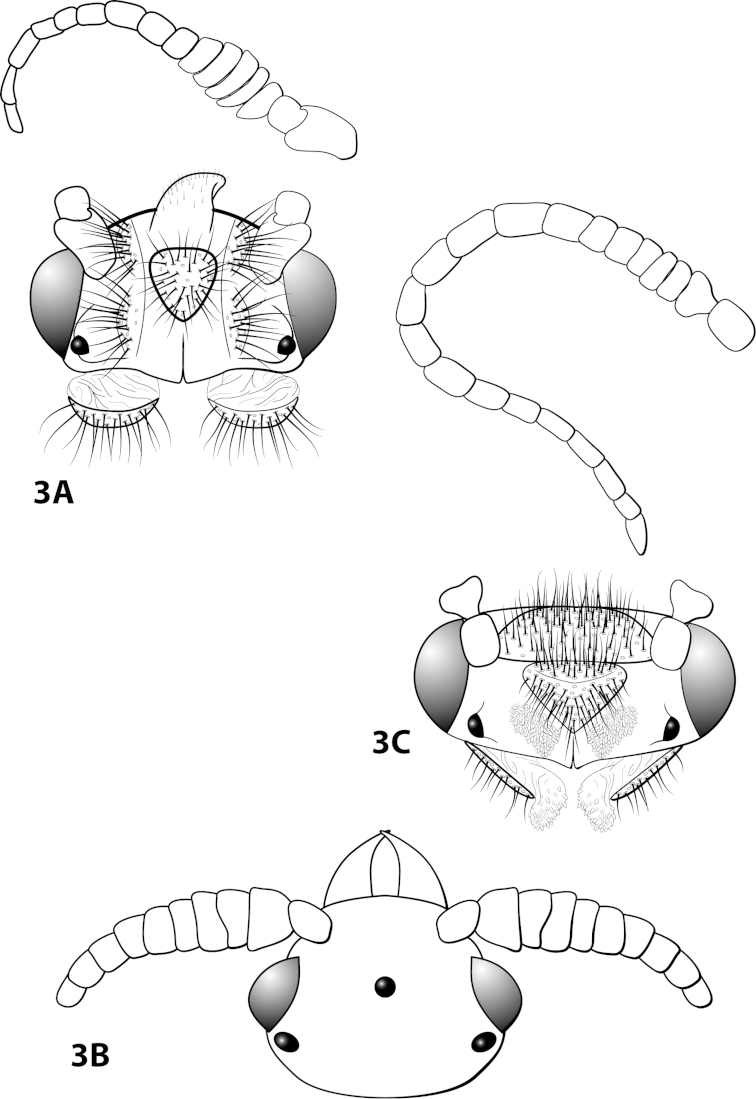
Head and antennae: **A** *Leucotrichia fairchildi* Flint, 1970 (UMSP000140357) **B** *Leucotrichia inflaticornis* Botosaneanu, 1993 (UMSP000140327) **C** *Leucotrichia pictipes* (Banks, 1911) (MCZ11597).

**Figure 4.**
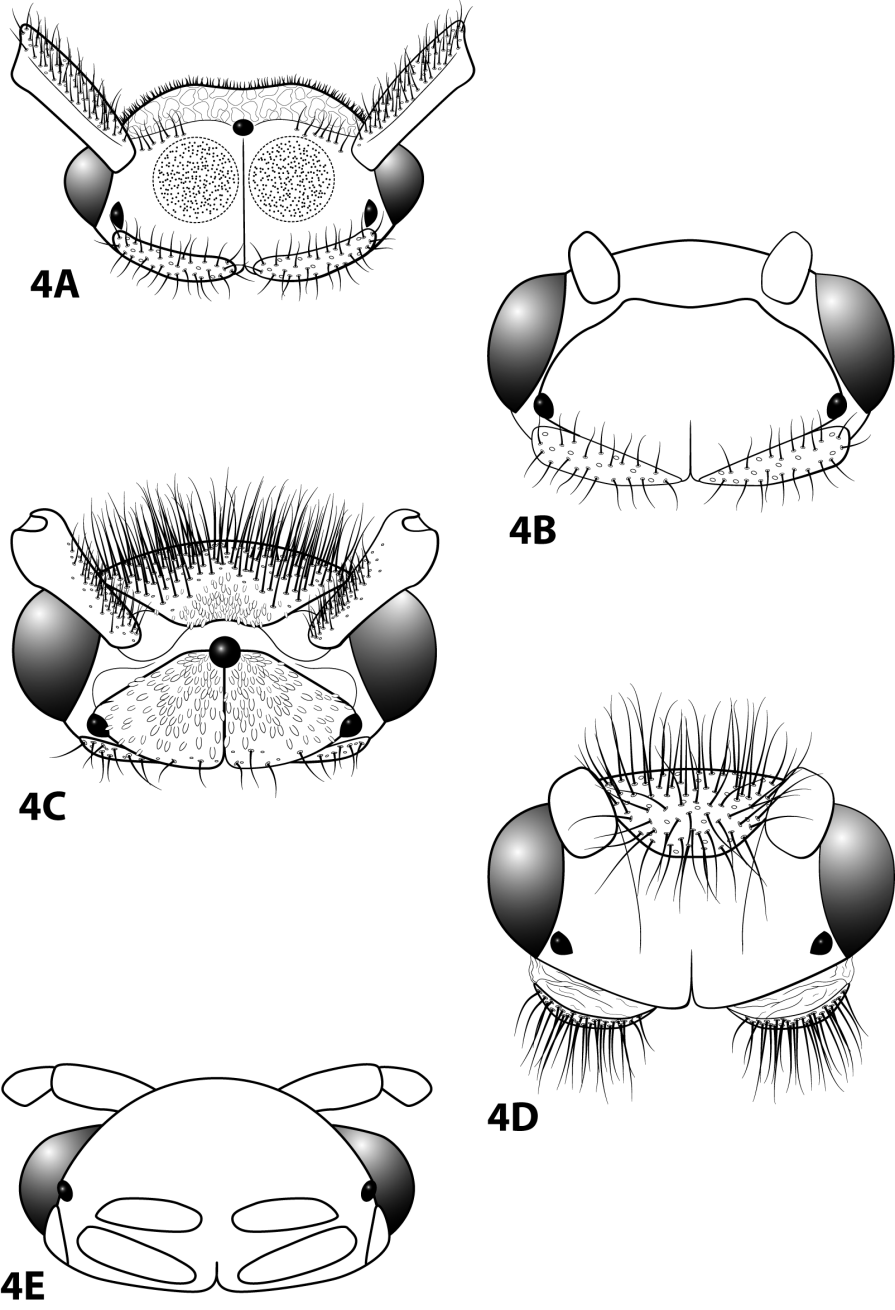
Head and scape: **A** *Leucotrichia chiriquiensis* Flint, 1970 (USNM70896) **B** *Leucotrichia imitator* Flint, 1970 (USNM70898) **C** *Leucotrichia procera* sp. n. (UMSP000047406) **D** *Leucotrichia sarita* Ross, 1944 (INHS22339) **E** *Leucotrichia yungarum* Angrisano & Burgos, 2002 (redrawn from Angrisano and Burgos 2002).

**Figure 7.**
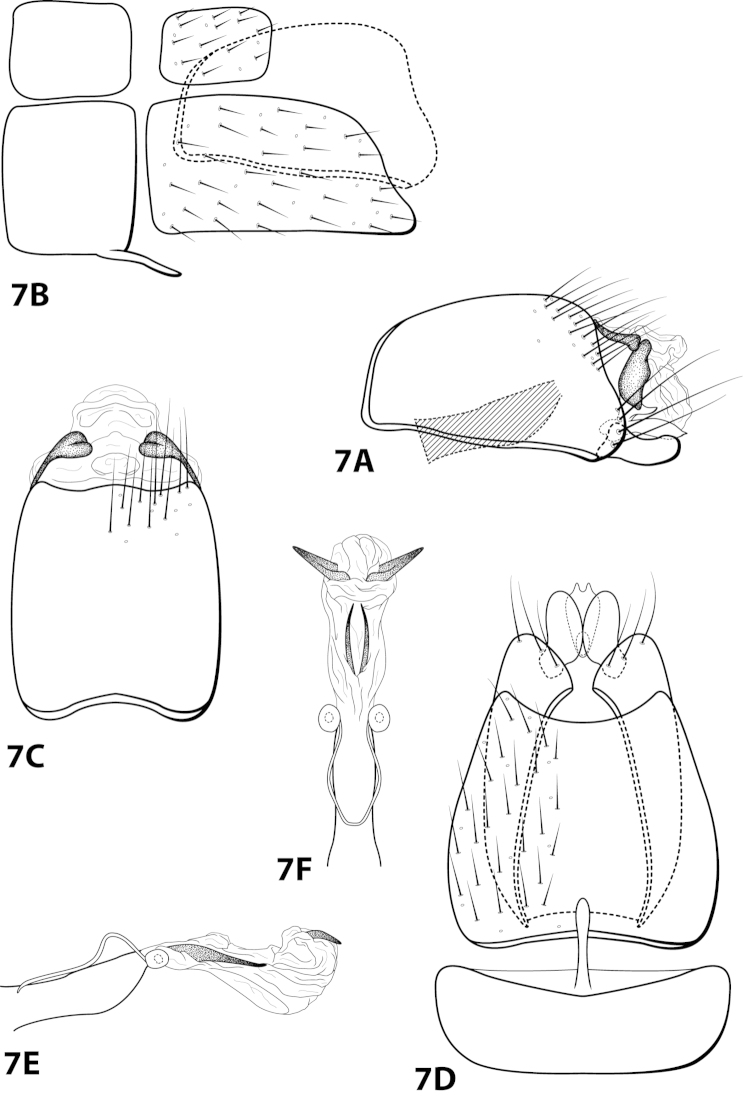
*Leucotrichia angelinae* sp. n. (UMSP000142591). Male genitalia: **A** segments IX–X, lateral (base of phallus crosshatched) **B** segments VII–VIII and segment IX margin, lateral **C** segments IX–X, dorsal **D** segments VII–IX, ventral **E** phallus, lateral **F** phallus, dorsal.

**Figure 8.**
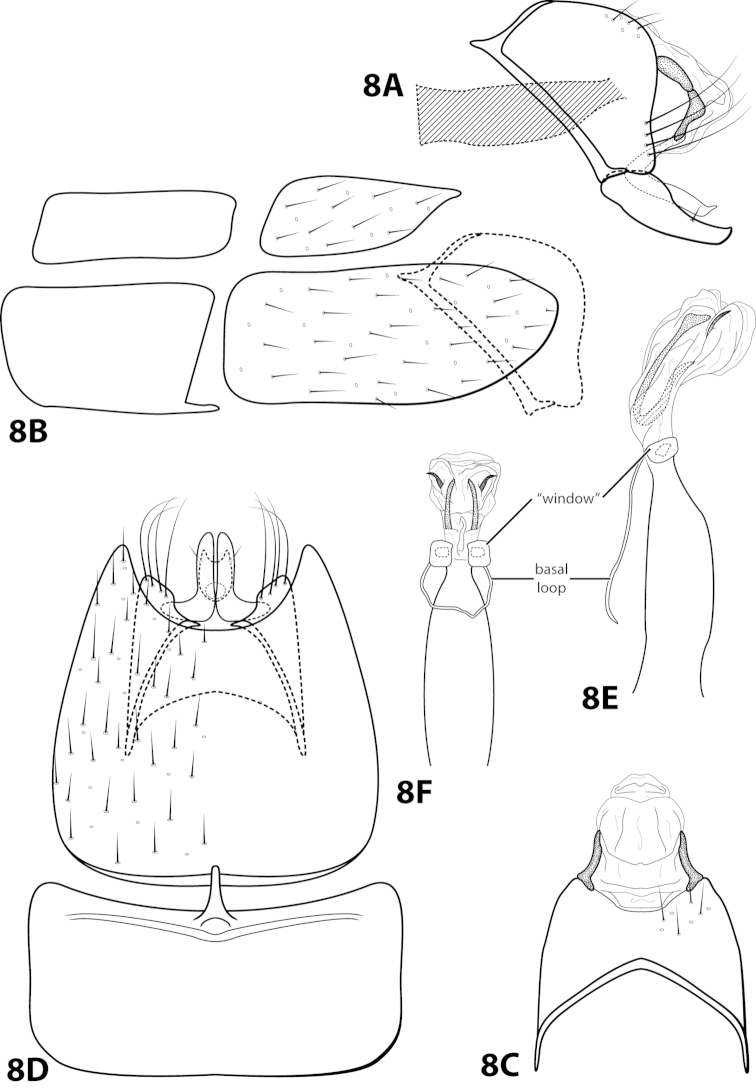
*Leucotrichia ayura* Flint, 1991 (USNM104529). Male genitalia: **A** segments IX–X, lateral (base of phallus crosshatched) **B** segments VII–VIII and segment IX margin, lateral **C** segments IX–X, dorsal **D** segments VII–IX, ventral **E** phallus, lateral **F** phallus, dorsal.

*Male*. Length of forewing ca. 2.0–5.0 mm. Wings unmodified, lacking a pouch, bulla, or patches of scales; forewing broad basally, acute apically; hind wing narrow, more acute than forewing, with row of hooked setae basal to cross vein r (Fig. 1E), edges with long setal fringe. Head with 2 or 3 ocelli; bearing setae and pair of setiferous posterolateral warts, dorsal region sometimes bearing modifications such as eversible posterolateral warts, patches of scales in place of setae, or setiferous protuberances (Figs 1A, 3, 4); antennae generally simple and unmodified, all flagellomeres of uniform size and shape, except in some species in which they may be elongate or have an inflated appearance (Figs 1A, 3). Maxillary palps with 5 segments, labial palps with 3 segments (Fig. 1B). Tibial spur count 1, 3, 4 (Fig. 1D). Mesoscutellum with transverse suture; metascutellum pentagonal (Fig. 1C). *Genitalia*. Abdominal sternum VII with single mesoventral process or tuft of prominent setae (Figs 2B, D, 34B). Sternum VIII produced posteroventrally beneath segment IX (Fig. 2B), with a posteromesal division (Fig. 2D). Segment IX open ventrally, sternum not developed (Fig. 2D), posterolateral margin with row of prominent setae (Fig. 2A). Tergum × with heavily sclerotized lateral plates, consisting of ventral and dorsal sclerites, and membranous apex (Fig. 2A). Subgenital plate connected dorsally to ventral angles of tergum × sclerites, produced ventrally as elongate mesal sclerite extending to base of inferior appendage, sometimes with dorsal arm (Fig. 10A), always with ventral arm (Fig. 2A, 10A). Inferior appendage simple, elongate, sometimes fused mesoventrally, generally with dorsal spine (Fig. 2A, D). Phallus tubular basally, constricted at midlength with median complex bearing basal loop and pair of spherical “windows” (Fig. 8E, F), basal loop sometimes extended on pair of basal supports (Fig. 10F); apex large, membranous, sac-like, generally bearing spines or sclerites.

##### Species relationships.

The species of *Leucotrichia* are divided into 2 main species groups based on adult features, as originally defined by Flint (1970). The *pictipes* species group is considerably smaller in species diversity than the *melleopicta* species group consisting of only *Leucotrichia fairchildi* Flint, 1970, *Leucotrichia imitator* Flint, 1970, *Leucotrichia pictipes* (Banks, 1911), and *Leucotrichia sarita* Ross, 1944 (Table 1).

Character states that distinguish members of the *Leucotrichia melleopicta* species group include males bearing 3 ocelli, a mesoventral process on sternum VII, an unmodified head (except for *Leucotrichia chiriquiensis* Flint, 1970), and 1 or 2 large patches of colored setae on the forewings. These characteristics all seem to represent the primitive state and should not be used to establish monophyly of the *melleopicta* group, although they can perhaps be used as diagnostic features to help identify species within the genus.

Character states distinguishing the *Leucotrichia pictipes* species group include males bearing 2 ocelli, either a brush of setae or a simple spine on sternum VII, a modified head (except for *Leucotrichia imitator*) as discussed above under the generic description, and colored spots or linear setal patterns on the forewings. These characteristics, particularly the reduced number of ocelli and the modifications to the head, may represent synapomorphies for this group of species within the genus.

While the specialized members of the *pictipes* group may form a monophyletic species group, the same cannot be said with certainty of the members of the *melleopicta* group, as they share no synapomorphies that could unite them. Despite being much larger in terms of species numbers, no synapomorphic adult characters were found within the *melleopicta* group that could be used to support its monophyly or to divide it into monophyletic species groups. The 2 species groups, *melleopicta* and *pictipes*, are retained here for historical relevance. All species newly described here have 3 ocelli and are placed in the *melleopicta* species group, which is consistent with its definition.

### Species descriptions

#### Leucotrichia melleopicta

Taxon classificationAnimaliaTrichopteraHydroptilidae

Mosely, 1934 type species

Figs 1 , 2

melleopicta Mosely, 1934: 157 [Type locality: Mexico, Tabasco, Teapa; NHM; male]. — Flint 1970: 5 [male], 1981: 25 [distribution].

##### Diagnosis.

This species is similar to *Leucotrichia mutica*. Both of these species possess an inferior appendage that is broadest mesally in lateral view and digitate in ventral view and the dorsal sclerite of the phallus in each species has an apical emargination. *Leucotrichia melleopicta* can be recognized by the enlarged apex of the mesoventral process on sternum VII, the apical emargination of the ventral arm of the subgenital plate, and the basal fusion of the inferior appendages.

##### Description.

*Male*. Length of forewing 1.9–2.5 mm (n=9). Head unmodified, with 3 ocelli; antennae unmodified. Color in alcohol brown. *Genitalia*. Abdominal sternum VII with elongate mesoventral process with enlarged apex. Sternum VIII in ventral view with posterior margin concave with rounded mesal emargination. Segment IX anterolateral margin straight, posterolateral margin convex; in dorsal view anterior margin concave, posterior margin straight. Tergum × with dorsal sclerite slender; ventral sclerite semielliptic with tridentate posterior margin; membranous apex not well developed. Subgenital plate with dorsal arm not apparent; ventral arm arched mesally, apex slightly flared (Fig. 2A), in ventral view with rounded apical emargination. Inferior appendage broadest mesally, apex rounded, bearing single dorsal spine; in ventral view fused basally, subspatulate. Phallus with “windows” of median complex not apparent, basal loop composed of 2 separate filaments, apex bearing dorsal sclerite with crenulate margins and pair of elongate internal sclerites.

##### Material examined.

*Holotype male*: **MEXICO: Tabasco:** Teapa, collected in March, H. H. Smith, slide mounted (NHM). *Nontypes*: **MEXICO: Veracruz:** Laguna Escondido, 28.iii.1976, J. Bueno, 7 males, 2 females (in alcohol) (NMNH); **VENEZUELA: Aragua:** Río El Limón, fish hatchery, Maracay, 3-6.ii.1976, C.M. and O.S. Flint, Jr., 1 female (NMNH); Río El Limón, fish hatchery, Maracay, 19-20.v.1975, F.H. Weibezahn, 1 male (in alcohol) (NMNH).

##### Etymology.

Unknown.

#### Leucotrichia adela

Taxon classificationAnimaliaTrichopteraHydroptilidae

Wells & Wichard, 1989 †

Fig. 5

†adela Wells & Wichard, 1989: 42 [Type locality: Dominican Republic NMNH; male; in amber].

##### Diagnosis.

This species is known only from the male fossil holotype preserved in Dominican amber. While some characteristics of *Leucotrichia adela* were originally described as difficult to examine and understand, Wells and Wichard (1989) did remark that the species shared similarities in the shape and form of abdominal segment IX and the genitalia with those of *Leucotrichia chiriquiensis* and *Leucotrichia tubifex*. It seems appropriate to place it in the *melleopicta* species group, due to the presence of 3 ocelli, an unmodified head, and a mesoventral process on sternum VII.

##### Description.

Redescribed from Wells and Wichard (1989). *Male*. Length of forewing 1.4 mm (n=1). Head unmodified, with 3 ocelli; antennae unmodified. Tibial spur count 1, 3, 4. *Genitalia*. Genitalia obscured and difficult to interpret. Abdominal sternum VII with slender, elongate mesoventral process. Sternum VIII in ventral view with posterior margin concave. Posterior margin of segment IX concave in dorsal view. Membranous apex of tergum × rounded. Inferior appendage bract-like.

##### Material examined.

*Holotype male*: Fossil holotype deposited at NMNH, but unable to be located.

##### Etymology.

Unknown.

#### Leucotrichia alisensis

Taxon classificationAnimaliaTrichopteraHydroptilidae

Rueda Martín, 2011

Fig. 6

alisensis Rueda Martín, 2011: 4 [Type locality: Argentina, Tucamán, Parque Nacional Campo de Los Alisos, Río de las Pavas; IML; male metamorphotype; larva, pupa].

##### Diagnosis.

*Leucotrichia alisensis* is similar to *Leucotrichia yungarum*; in each, the ventral arm of the subgenital plate is acute in lateral view and the apex of the inferior appendage is digitate in ventral view. Additionally, the shape of the inferior appendage in lateral view is similar in both species, particularly the rounded basal area. *Leucotrichia alisensis* can be recognized by the V-shaped concave posterior margin of sternum VIII, which is U-shaped in *Leucotrichia yungarum*, and by the curved anterolateral projection which is absent in *Leucotrichia yungarum*.

##### Description.

Redescribed from Rueda Martín (2011). *Male*. Head unmodified, with 3 ocelli; antennae unmodified. *Genitalia*. Abdominal sternum VII with mesoventral process not apparent. Sternum VIII in ventral view with posterior margin concave with small mesal emargination. Segment IX anterolateral margin with curved ventrolateral projection, posterolateral margin irregular. Membranous apex of tergum × slender, extending posteriad, with mesodorsal projection. Subgenital plate with dorsal arm not apparent; ventral arm narrow, apex acute. Inferior appendage rounded basally, apex digitate; in ventral view basally subquadrate. Phallus apex with light dorsal sclerites.

##### Material examined.

*Holotype male*: Holotype deposited at IML, but could not be obtained.

##### Etymology.

Named for the National Park Campo de Los Alisos, Tucamán, Argentina, the location where the holotype was collected.

#### Leucotrichia angelinae

Taxon classificationAnimaliaTrichopteraHydroptilidae

Thomson & Holzenthal sp. n.

http://zoobank.org/A0D99288-A515-455E-AC82-967548EDE690

Fig. 7

##### Diagnosis.

*Leucotrichia angelinae* sp. n., is most similar to *Leucotrichia fulminea* sp. n. These species share several similarities in the mesoventral process of sternum VII, inferior appendage, subgenital plate, and phallus, as discussed under *Leucotrichia fulminea*. In ventral view, the inferior appendage of *Leucotrichia angelinae* has an oval shape, while the inner margin in *Leucotrichia fulminea* is concave. *Leucotrichia angelinae* can also be distinguished by the presence of a short dorsal arm on the subgenital plate, which is lacking in *Leucotrichia fulminea*.

##### Description.

*Male*. Length of forewing 3.6–4.4 (n=3). Head unmodified, with 3 ocelli; antennae unmodified. Dorsum of head dark brown with light green and dark brown setae; thorax dark brown with dark brown setae and mint green setae at edges, brown ventrally; leg segments with brown setae. Forewings covered with fine mottled light gray-green setae with dark brown setae along edges. *Genitalia*. Abdominal sternum VII with digitate mesoventral process. Sternum VIII in ventral view with posterior margin concave. Segment IX anterolateral margin convex, posterolateral margin irregular; in dorsal view anterior margin shallowly concave, posterior margin irregular. Tergum × with dorsal sclerite simple; ventral sclerite semielliptic with tridentate posterior margin; membranous apex with dorsal and ventral lobes. Subgenital plate with dorsal arm short, curved dorsad, tapering apically; ventral arm elliptic with basal projection, in ventral view obovate with 2 small apical projections. Inferior appendage apex rounded, without dorsal spine; in ventral view oval, with subquadrate basal projection on outer margin. Phallus apex bearing pair of slender mesodorsal sclerites and pair of pointed apicodorsal sclerites extending outwards.

*Holotype male*: **VENEZUELA: Mérida:** Cacuta, 10 km E Tabay, 22.ii.1976, C.M. and O.S. Flint (UMSP000142591) (NMNH). *Paratypes*: Same data as holotype, 1 male, 1 female (NMNH); 19 km N Mérida, 5600 ft, 9.ii.1978, blacklight, Andean mountain forest, J.B. Heppner, 1 male (UMSP).

##### Etymology.

Named in honor of Angel Fowler, friend and colleague, for all her help in the field.

#### Leucotrichia ayura

Taxon classificationAnimaliaTrichopteraHydroptilidae

Flint, 1991

Fig. 8

ayura Flint, 1991: 41 [Type locality: Colombia, Dpto. Antioquia, 12 km NW Medellín, road to San Pedro; NMNH; male].

##### Diagnosis.

*Leucotrichia ayura* is most similar to *Leucotrichia repanda* sp. n. Both species bear a small mesoventral process on sternum VII, an elongate sternum VIII with a concave posterolateral margin, an anterolaterally produced margin on segment IX, an inferior appendage curving slightly dorsad with a truncate apex, and a phallus apex with elongate dorsal sclerites. The elongate sclerites in *Leucotrichia ayura* are U-shaped in lateral view, making this species distinct from *Leucotrichia repanda*. The phallus apex of *Leucotrichia ayura* also bears a pair of small subapicodorsal spines that are absent in *Leucotrichia repanda*. Additionally, the inferior appendages of *Leucotrichia ayura* are separate, while they are fused in *Leucotrichia repanda*.

##### Description.

*Male*. Length of forewing 2.3–2.9 mm (n=8). Head unmodified, with 3 ocelli; antennae unmodified. Dorsum of head dark brown with bright yellow setae; thorax dark brown with bright yellow setae dorsally, brown ventrally; leg segments with dark brown setae. Forewings covered with fine bright yellow setae, apical 1/2 with brown setae. *Genitalia*. Abdominal sternum VII with small, digitate mesoventral process. Sternum VIII in ventral view with posterior margin concave. Segment IX anterolateral margin slightly produced mesally, posterolateral margin with shallow mesal depression; in dorsal view anterior margin concave, posterior margin concave. Tergum × with dorsal sclerite simple; ventral sclerite with rounded projection on posterior margin; membranous apex rounded. Subgenital plate with dorsal arm not apparent; ventral arm digitate with small apical projection, in ventral view with rounded apical emargination. Inferior appendage broadest basally, apex truncate, bearing single dorsal spine; in ventral view digitate, with basal projection on outer margin. Phallus apex bearing pair of elongate dorsal sclerites (U-shaped and with slightly enlarged dorsal apex in lateral view) and pair of small, dark, subapicodorsal spines.

##### Material examined.

*Holotype male*: COLOMBIA: Antioquia: 12 km NW Medellín, rd. to San Pedro, 13.ii.1983, O.S. Flint, Jr. (USNM 104529) (NMNH). *Paratypes*: Same data as holotype, 2 males, 1 female (NMNH); COLOMBIA: Antioquia: Quebrada Agua Mala, 34 km NW Medellín, 14.ii.1983, O.S. Flint, Jr., 2 males (NMNH); 12 km NW Medellín, rd. to San Pedro, 20.ii.1984, C.M. and O.S. Flint, Jr., 1 male (NMNH); Quebrada La Ayurá above Envigado, 21.ii.1984, C.M. and O.S. Flint, Jr., 1 male (NMNH); Quebrada La Ayurá, Mun. Envigado, elev. 1750 m, 29.iii-28.iv.1983, trap B, U. Matthias, 3 males, 2 females (in alcohol) (NMNH). *Nontypes*: COLOMBIA: Antioquia: 7 km E San Jerónimo, 23.ii.1984, C.M. and O.S. Flint, Jr., 1 female (NMNH).

##### Etymology.

Presumably named for Quebrada La Ayurá, the location where some of the paratypes were collected.

#### Leucotrichia bicornuta

Taxon classificationAnimaliaTrichopteraHydroptilidae

Thomson, 2012

Fig. 9

bicornuta Thomson, 2012: 4 [Type locality: Brazil, Río de Janeiro, Panedo, Río das Pedras, Três Bacias; DZRJ; male].

##### Diagnosis.

This species is similar to *Leucotrichia dianeae* sp. n., *Leucotrichia extraordinaria*, and *Leucotrichia tapantia* sp. n. In each of these species, the posterolateral margin of sternum VIII is produced, with the apex of the projection bearing either prominent setae or a peg-like seta. The shape of the phallus apex is also similar in all these species, with all bearing a pair of membranous apical lobes and lacking any external spines or sclerites. *Leucotrichia bicornuta* differs from the other 3 species in having fused inferior appendages and a distinct, irregularly shaped dorsal arm on the subgenital plate.

##### Description.

**Figure 9.**
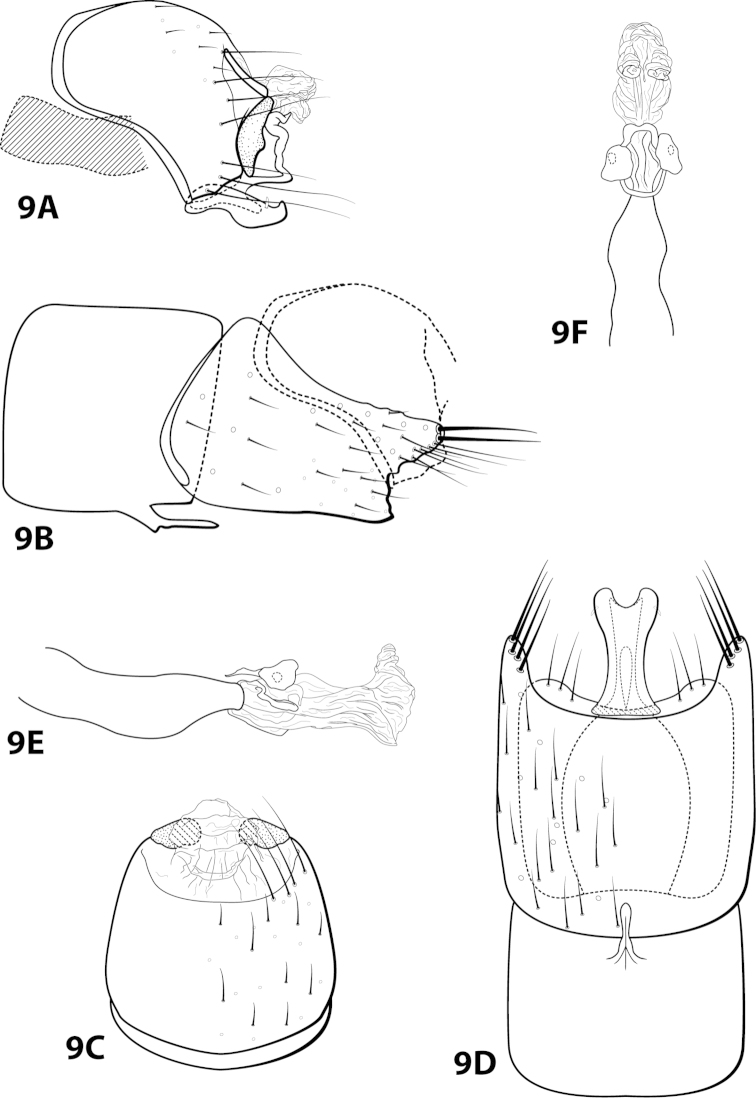
*Leucotrichia bicornuta* Thomson, 2012 (UMSP000014084). Male genitalia: **A** segment IX–X, lateral (base of phallus crosshatched) **B** segments VII–VIII and segment IX margin, lateral **C** segments IX–X, dorsal **D** segments VII–IX, ventral **E** phallus, lateral **F** phallus, dorsal.

**Figure 10.**
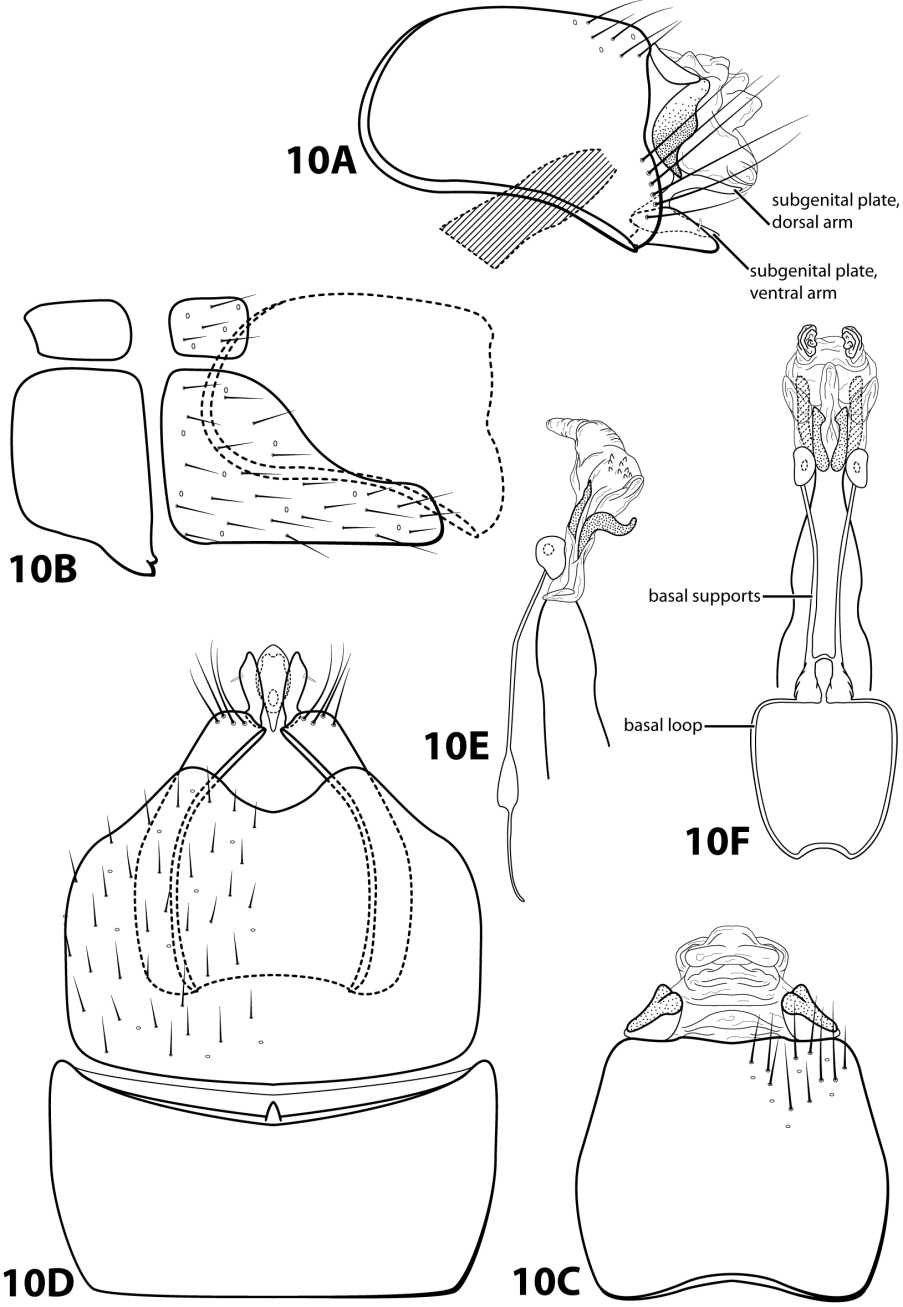
*Leucotrichia botosaneanui* Flint, 1996 (USNM105436). Male genitalia: **A** segments IX–X, lateral (base of phallus crosshatched) **B** segments VII–VIII and segment IX margin, lateral **C** segments IX–X, dorsal **D** segments VII–IX, ventral **E** phallus, lateral **F** phallus, dorsal.

*Male*. Length of forewing 2.5–3.4 mm (n=3). Head unmodified, with 3 ocelli; antennae unmodified. Color in alcohol brown. *Genitalia*. Abdominal sternum VII with digitate mesoventral process with small basal ridge. Sternum VIII posteromesal projection bearing long, prominent setae; in ventral view with posterior margin concave. Segment IX anterolateral margin broadly produced dorsolaterally, posterolateral margin irregular; in dorsal view anterior margin convex, posterior margin concave. Tergum × with dorsal sclerite slender; ventral sclerite semielliptic with tridentate posterior margin; membranous apex suborbicular. Subgenital plate with dorsal arm irregular, bent sharply dorsad (Fig. 9A); ventral arm short, digitate, with basal projection, laterally obscured from view by inferior appendage (Fig. 9A), in ventral view with rounded apical emargination. Inferior appendage arched mesally, apex hooked dorsally, bearing single dorsal spine; in ventral view entirely fused, apex emarginated. Phallus apex bearing paired apicodorsal lobes, no spines or sclerites apparent.

##### Material examined.

*Holotype male*: **BRAZIL: Río de Janeiro:** Panedo, Río das Pedras, Três Bacias, 22°24'32.2"S, 44°33'06.6"W, elev. 735 m, 6.iii.2008, Nessimian, Dumas, de Souza, and Braga (in alcohol) (UMSP000014084) (DZRJ). *Paratypes*: **BRAZIL: Río de Janeiro:** Itatiaia, Parque Nacional do Itatiaia, Río Camp Belo, 22°27'17.32"S, 44°36'37.47"W, elev. 705 m, 13.iv.2007, light, Santos, Dumas, Ferreira, Jr., and Nessimian, 2 males, 1 female (in alcohol) (DZRJ).

##### Etymology.

*Bi*, Latin for “double”; *cornutus*, Latin for “horned”, referring to the 2 apicodorsal lobes of the phallus.

#### Leucotrichia botosaneanui

Taxon classificationAnimaliaTrichopteraHydroptilidae

Flint, 1996

Fig. 10

botosaneanui Flint, 1996: 86 [Type locality: Tobago, big waterfall 4 km S Charlotteville, NMNH; male]. — Botosaneanu and Sakal 1992: 201 [biology, as *limpia*]. — Botosaneanu and Alkins-Koo 1993: 8 [larva, as *limpia*, according to Flint 1996: 86].

##### Diagnosis.

*Leucotrichia botosaneanui* is most similar to *Leucotrichia chiriquiensis*, *Leucotrichia hispida* sp. n., *Leucotrichia limpia*, and *Leucotrichia viridis*. In all these species, the phallus bears a similar combination of characteristics: elongate basal supports on the midlength complex; a pair of small, membranous, apical lobes; and a membranous “bulge” on the ventral surface. Additionally, the posterolateral margin of sternum VIII is produced in these species. *Leucotrichia botosaneanui* can be separated from the other 4 species by the small, double-pointed mesoventral process on sternum VII and the pair of lateral sinuate sclerites present ventrolaterally on the phallus apex.

##### Description.

*Male*. Length of forewing 2.0–2.3 mm (n=6). Head unmodified, with 3 ocelli; antennae unmodified. Dorsum of head dark brown with yellow and dark brown setae; thorax dark brown with dark brown and yellow setae dorsally, brown ventrally; leg segments with brown setae. Forewings covered with fine yellow setae, apical 1/4 with dark brown setae. *Genitalia*. Abdominal sternum VII with small, double-pointed mesoventral process. Sternum VIII in ventral view with posterior margin concave. Segment IX anterolateral margin broadly produced dorsolaterally; in dorsal view anterior margin shallowly concave, posterior margin straight. Tergum × with dorsal sclerite simple; ventral sclerite semielliptic with tridentate posterior margin; membranous apex. Subgenital plate with dorsal arm slender, digitate; ventral arm tapering apically, in ventral view with small apical emargination and slightly subapically constricted. Inferior appendage short, with small basal emargination, broadest mesally, bearing single dorsal spine; in ventral view subspatulate, apex slightly produced. Phallus with median complex bearing elongate basal supports, apex bearing pair of apicodorsal lobes, pair of dark dorsal sclerites, and pair of lateral sinuate sclerites.

##### Material examined.

*Holotype male*: **TOBAGO:** Charlotteville, 4 km S, big waterfall, elev. 125 m, 11°19'N, 60 °33'W, 10vi.1993, by net, O.S. Flint, Jr. and W.N. Mathis (USNM 105436) (NMNH). *Paratypes*: Same data as holotype, 3 males, 2 females (NMNH); same data as holotype, 1 male (in alcohol) (NMNH); **TRINIDAD:** streamlet below Maracas Waterfall, elev. 250 m, 10°44'N, 61°24'W, 18.vi.1993, UV light, N.E. Adams and W.N. Mathis, 1 female (NMNH); Lalaja Rd. streamlet, elev. 520 m, 10°43'N, 61°17'W, 26.vi.1993, UV light, N.E. Adams and W.N. Mathis, 1 male, 1 female (NMNH).

##### Etymology.

Named in honor of the late Dr. Lazare Botosaneanu, a naturalist and entomologist formerly of the Zoological Museum of the University of Amsterdam.

#### Leucotrichia brasiliana

Taxon classificationAnimaliaTrichopteraHydroptilidae

Sattler & Sykora, 1977

Fig. 11

brasiliana Sattler & Sykora, 1977: 239 [Type locality: Brazil, Amazonas Staat, bereich des Rio Marauía, bei Tapuruquara, oberer Rio Negro; type depository unknown; male; larva, pupa, case].

##### Diagnosis.

**Figure 11.**
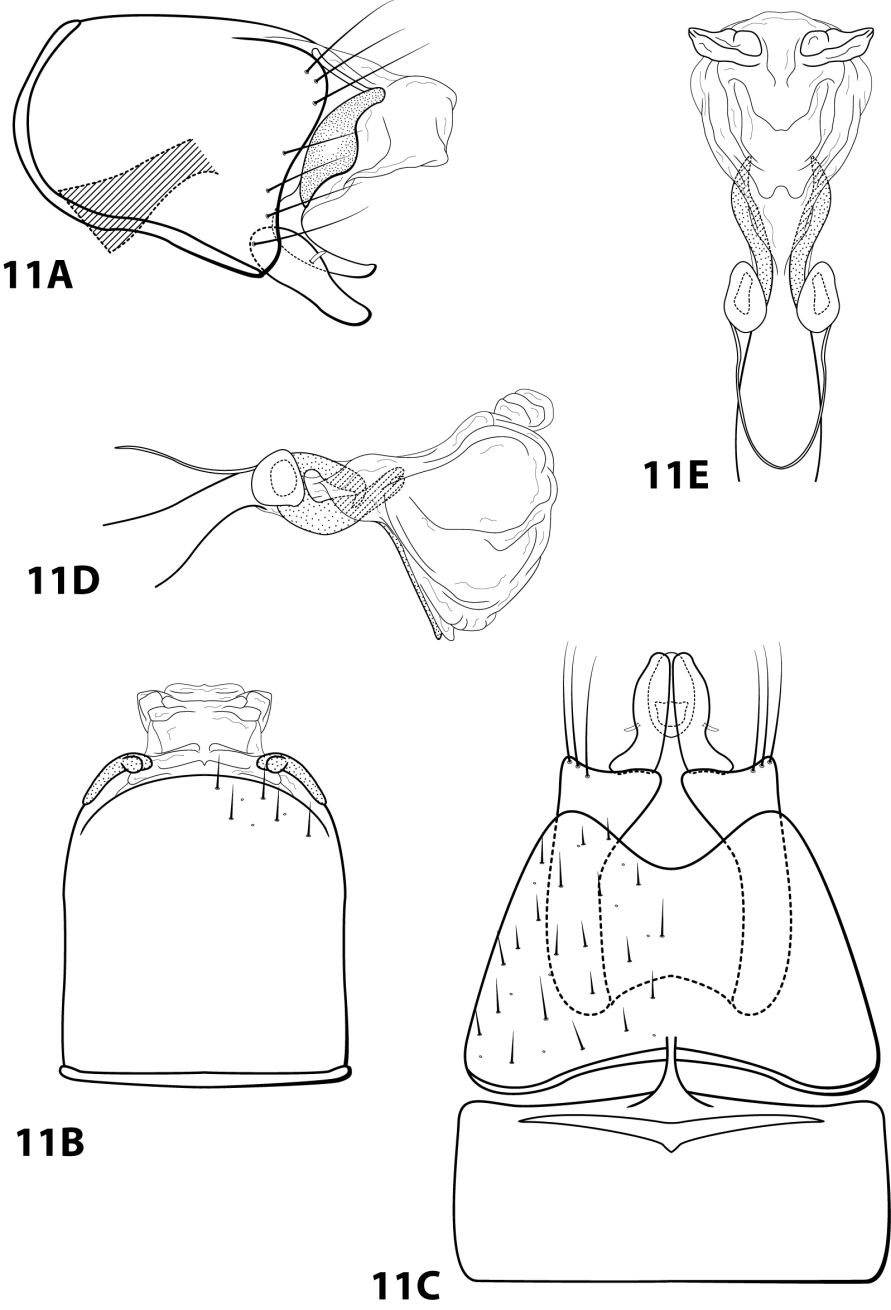
*Leucotrichia brasiliana* Sattler & Sykora, 1977 (UMSP000140138). Male genitalia: **A** segments IX–X, lateral (base of phallus crosshatched) **B** segments IX–X, dorsal **C** segments VII–IX, ventral **D** phallus, lateral **E** phallus, dorsal.

This species is most similar to *Leucotrichia sidneyi* sp. n. The phallus apex in both species bears a membranous “bulge” on the ventral surface (Fig. 11D), a pair of apical lobes, and lacks apical spines or sclerites. In *Leucotrichia brasiliana*, the phallus apex bears a pair of lateral sclerites that are U-shaped in lateral view and not present in *Leucotrichia sidneyi*. The membranous portion of the phallus apex of *Leucotrichia brasiliana* is truncate in both lateral and dorsal view, while it is small and rounded in *Leucotrichia sidneyi*.

##### Description.

Holotype unknown, topotypes pharate adults. *Male*. Length of forewing 1.3–1.7 (n=2). Head unmodified, but with mandibles (pharate adult), with 3 ocelli; antennae unmodified. Color in alcohol brown. *Genitalia*. Abdominal sternum VII with mesoventral process broken (indicated in Fig. 11C). Sternum VIII torn in all specimens and difficult to examine in lateral view; in ventral view with posterior margin concave. Segment IX anterolateral margin convex, posterolateral margin with shallow mesal depression; in dorsal view anterior margin straight, posterior margin broadly concave. Tergum × with dorsal sclerite slender, elongate; ventral sclerite large, semielliptic, with tridentate posterior margin; membranous apex truncate. Subgenital plate with dorsal arm not apparent; in ventral view ovate. Inferior appendage broadest basally, apex rounded, bearing single dorsal spine; in ventral view broadly rounded on outer margin. Phallus apex bearing pair of elongate sclerites (U-shaped in lateral view) and pair of apicodorsal lobes.

##### Material examined.

*Topotypes*: **BRAZIL: Amazonas:** from Sattler collection 23, Sattler, 2 larvae, 2 male metamorphotypes, 4 adult metamorphotypes of undetermined gender (NMNH).

##### Etymology.

Presumably named for the country of Brazil, where the species was collected.

#### Leucotrichia brochophora

Taxon classificationAnimaliaTrichopteraHydroptilidae

Flint, 1991

Fig. 12

brochophora Flint, 1991: 41 [Type locality: Colombia, Dpto. Antioquia, Quebrada Espadera, 7 km E Medellín, road to Sta. Elena; NMNH; male].

##### Diagnosis.

This species is most similar to *Leucotrichia lerma*, *Leucotrichia padera*, and *Leucotrichia rhomba* sp. n. All these species possess a phallus with no spines and few, if any, elongate sclerites on the membranous apex; a mild, broad protuberance on the posterolateral margin of sternum VIII; and anterolateral margin on segment IX produced. *Leucotrichia brochophora* can be recognized by the presence of basal supports on the basal loop of the phallus midlength complex, a small mesoventral process on sternum VI, and stout pegs on the posterolateral margin of segment IX, all of which are absent in the other 3 species.

##### Description.

**Figure 12.**
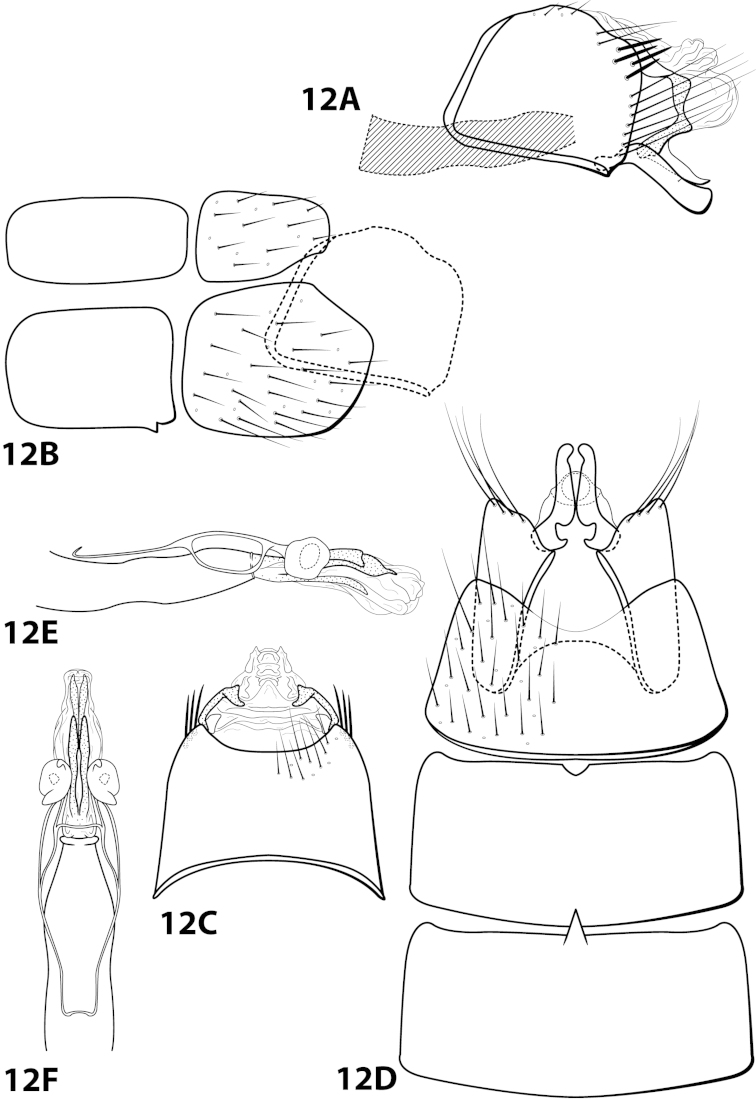
*Leucotrichia brochophora* Flint, 1991 (USNM104527). Male genitalia: **A** segments IX–X, lateral (base of phallus crosshatched) **B** segments VII–VIII and segment IX margin, lateral **C** segments IX–X, dorsal **D** segments VI–IX, ventral **E** phallus, lateral **F** phallus, dorsal.

*Male*. Length of forewing 5.2 mm (n=1). Head unmodified, with 3 ocelli; antennae unmodified. Dorsum of head cleared in holotype, brown; thorax brown with dark brown and yellow setae dorsally, brown ventrally; leg segments with light brown setae. Forewings covered with fine yellow setae, apical 1/3 with dark brown setae. *Genitalia*. Abdominal sternum VI with small pointed mesoventral process (Fig. 12D), obscured by setae in lateral view. Abdominal sternum VII with mesoventral process broken (indicated in Fig. 12D). Sternum VIII in ventral view with posterior margin concave. Segment IX anterolateral margin convex, posterolateral margin convex with stout peg-like setae dorsolaterally; in dorsal view anterior margin concave, posterior margin concave. Tergum × with dorsal sclerite simple; ventral sclerite lower half bent anteriad; membranous apex suborbicular. Subgenital plate with dorsal arm not apparent; ventral arm tapering apically, in ventral view apex broadly rounded. Inferior appendage with knoblike basal projection, apex truncate, bearing single dorsal spine; in ventral view with subbasal subtriangular emargination on inner margin, apex rounded. Phallus with median complex bearing basal supports; apex bearing 3 pairs of elongate sclerites: 1st pair dorsal and with shallow apical emargination, 2nd pair dorsal and with small apical projection, 3rd pair lateral and slender.

##### Material examined.

*Holotype male*: **COLOMBIA: Antioquia:** Quebrada Espadera, 7 km E Medellín, road to Sta. Elena, 24.ii.1983, O.S. Flint, Jr. (USNM 104527) (NMNH).

##### Etymology.

Unknown.

#### Leucotrichia chiriquiensis

Taxon classificationAnimaliaTrichopteraHydroptilidae

Flint, 1970

Figs 4A , 13

chiriquiensis Flint, 1970: 6 [Type locality: Panama, Chiriqui, Alto Lino above Boquete; NMNH; male; larva, case].

##### Diagnosis.

*Leucotrichia chiriquiensis* is most similar to *Leucotrichia botosaneanui*, *Leucotrichia hispida* sp. n., *Leucotrichia limpia*, and *Leucotrichia viridis*. These species share a similar combination of characteristics present in the phallus and the posterolateral margin of sternum VIII, as discussed under *Leucotrichia botoseaneanui*. This species differs from the other 4 in having a small set of dorsal hooks between the apical lobes of the phallus apex. It can most easily be separated from the others by the modifications of the antennae and head.

##### Description.

*Male*. Length of forewing 2.1–2.9 mm (n=31). Head (Fig. 4A) with short, black, contiguous setae anteriorly (which leave irregular shapes when removed) and a pair of internal structures filled with similar setae, with 3 ocelli; antennae with scape elongate, anterior face indented and filled with dense patch of setae. Dorsum of head dark brown with light yellow setae; thorax dark brown with yellow setae dorsally, brown ventrally; leg segments with brown setae. Forewings covered with fine yellow setae, apical 1/4 with patch of brown setae. *Genitalia*. Abdominal sternum VII with slender, elongate mesoventral process with apex enlarged, rugose in ventral view. Sternum VIII in ventral view with posterior margin concave. Segment IX anterolateral margin broadly produced dorsolaterally, posterolateral margin with shallow mesal depression; in dorsal view anterior margin concave, posterior margin broadly convex. Tergum × with dorsal sclerite slender, bent ventrad; ventral sclerite semielliptic with tridentate posterior margin; membranous apex suborbicular. Subgenital plate bent mesally; with dorsal arm not apparent; ventral arm tapering apically, curved ventrad, in ventral view base subtriangular and apex rounded. Inferior appendage dorsomesally “humped,” bearing single dorsal spine; in ventral view with small subapical emargination on inner margin. Phallus with median complex bearing elongate basal supports, apex bearing pair of elongate lateral sclerites and pair of dorsal hooks between pair of apical lobes.

##### Material examined.

*Holotype male (pharate)*: **PANAMA: Chiriqui:** Alto Line above Boquete, 16–17.vii.1967, O.S. Flint, Jr. and Ortiz, (in alcohol) (USNM 70896) (NMNH). *Paratypes*: Same data as holotype, 2 males, 1 female (pharates) (in alcohol) (NMNH). *Nontypes*: **COSTA RICA:** Cartago, Reserva Tapantí, Río Grande de Orosi, elev. 1650 m, 9.686N, 83.756W, 18–21.iii.1987, Holzenthal, Hamilton, and Heyn, 28 males; Cartago, Reserva Tapantí, Río Grande de Orosi, elev. 1650 m, 9.686°N, 83.756°W, 18-21.iii.1987, Holzenthal, Hamilton, and Heyn, 5 females (UMSP).

##### Etymology.

Presumably named for Chiriquí Province in Panama, where the holotype was collected.

#### Leucotrichia denticulata

Taxon classificationAnimaliaTrichopteraHydroptilidae

Thomson & Holzenthal sp. n.

http://zoobank.org/8C67C863-E335-42F9-B2D5-49C1B8DC9A63

Fig. 14

##### Diagnosis.

**Figure 14.**
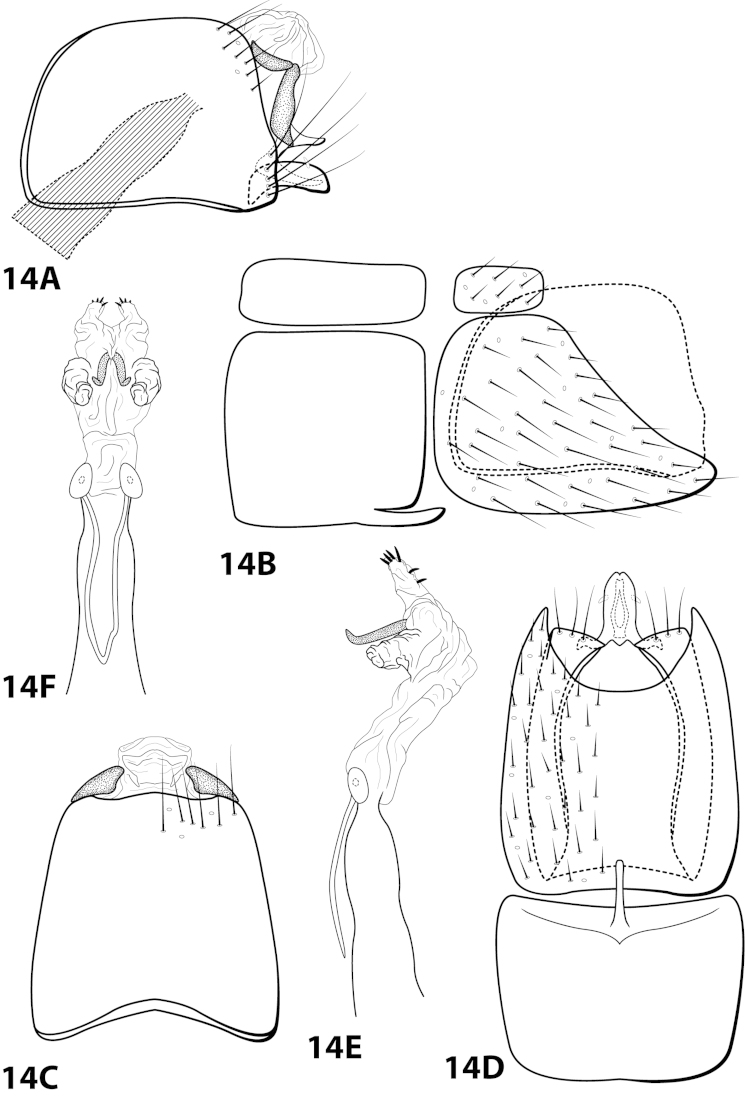
*Leucotrichia denticulata* sp. n. (UMSP000142916). Male genitalia: **A** segments IX–X, lateral (base of phallus crosshatched) **B** segments VII–VIII and segment IX margin, lateral **C** segments IX–X, dorsal **D** segments VII–IX, ventral **E** phallus, lateral **F** phallus, dorsal.

*Leucotrichia denticulata* sp. n., can be separated from all other species in teh genus by a suite of characteristics found on the phallus. The presence of the pair of mesolateral lobes, pair of large apically-hooked sclerites, and pair of apical lobes bearing peg-like setae distinguish the phallus from that of *Leucotrichia denticulata* (Figs 14E, F).

##### Description.

*Male*. Length of forewing 3.7–4.2 mm (n=2). Head unmodified, with 3 ocelli; antennae unmodified. Color in alcohol brown. *Genitalia*. Abdominal sternum VII with digitate mesoventral process. Sternum VIII in ventral view with posterior margin concave. Segment IX anterolateral margin convex, posterolateral margin irregular; in dorsal view anterior margin shallowly concave, posterior margin straight. Tergum × with dorsal sclerite simple; ventral sclerite semielliptic with crenulate posterior margin; membranous apex suborbicular. Subgenital plate with dorsal arm digitate; ventral arm rounded basally, tapering apically, in ventral view sublanceolate. Inferior appendage arched mesally, apex truncate, bearing single dorsal spine; in ventral view entirely fused, with basal projection on outer margin, apex emarginated. Phallus apex bearing pair of mesolateral lobes, pair of hooked mesodorsal sclerites, and pair of apical lobes with row of peg-like setae.

*Holotype male*: **MEXICO: Nuevo Leon:** Muncipio de Santiago, Arroyo San Juan on road to Laguna de Sanchez, 3.5 km W La Cienegra, 25°24'N, 100°17'W, 1400m, 13.v.1989, Harris and Contreras (in alcohol) (UMSP000142916) (UMSP). *Paratype*: Same data as holotype, 1 male (in alcohol) (UMSP).

##### Etymology.

*Denticulatus*, Latin for “with small teeth”, referring to the row of peg-like setae on the apical lobes of the phallus.

#### Leucotrichia dianeae

Taxon classificationAnimaliaTrichopteraHydroptilidae

Thomson & Holzenthal sp. n.

http://zoobank.org/FA7318B2-146B-4F39-81C4-EFED47096C09

Fig. 15

##### Diagnosis.

This species is similar to *Leucotrichia bicornuta*, *Leucotrichia extraordinaria*, and *Leucotrichia tapantia* sp. n. These species share a similar combination of characteristics present in the phallus and the posterolateral margin of sternum VIII, as discussed under *Leucotrichia bicornuta*. Of these species, *Leucotrichia dianeae* sp. n., is most similar to *Leucotrichia tapantia*, as discussed under *Leucotrichia tapantia*. The apical lobes of the phallus apex of *Leucotrichia dianeae* arise from the same apicomesal location on the apex, while those of *Leucotrichia tapantia* are separated from each other in more apicolateral locations. Additionally, the mesal projection on the posterior margin of sternum VIII is pointed in *Leucotrichia dianeae* and rounded in *Leucotrichia tapantia*. The rugose ventral surface of the apex of the mesoventral process on sternum VII separates *Leucotrichia dianeae* from all 3 of the other species.

##### Description.

**Figure 15.**
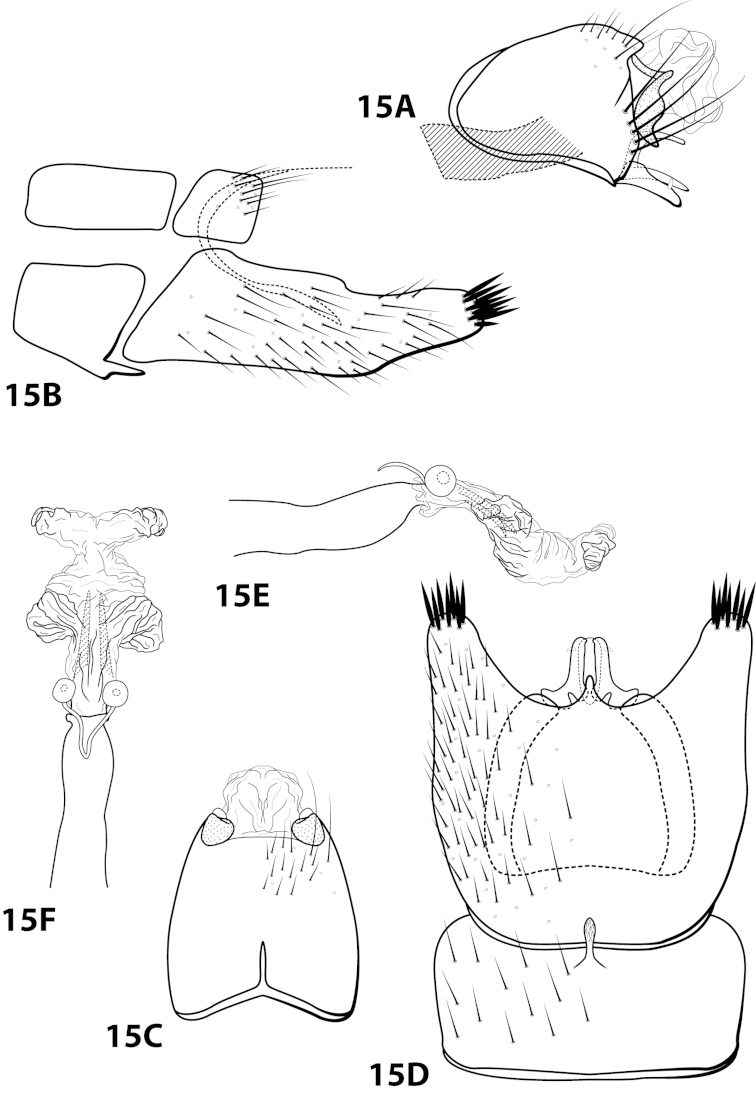
*Leucotrichia dianeae* sp. n. (UMSP000201649). Male genitalia: **A** segments IX–X, lateral (base of phallus crosshatched) **B** segments VII–VIII and segment IX margin, lateral **C** segments IX–X, dorsal **D** segments VII–IX, ventral **E** phallus, lateral **F** phallus, dorsal.

*Male*. Length of forewing 3.4–4.1 mm (n=10). Head unmodified, with 3 ocelli; antennae unmodified. Dorsum of head dark brown with yellow setae; thorax dark brown with dark brown and yellow setae dorsally, brown ventrally; leg segments with brown setae. Forewings covered with fine yellow setae with dark brown setae at edges and apex. *Genitalia*. Abdominal sternum VII with pointed, rugose mesoventral process with small basal ridge. Sternum VIII lateral projection elongate, extending dorsad, apex bearing tuft of prominent setae (Fig. 15B), in ventral view with posterior margin concave with small, pointed mesal projection (Fig. 15D). Segment IX anterolateral margin convex, posterolateral margin irregular; in dorsal view anterior margin concave, posterior margin straight. Tergum × with dorsal sclerite slender; ventral sclerite semielliptic with tridentate posterior margin; membranous apex subquadrate. Subgenital plate with dorsal arm digitate; ventral arm slender, arched mesally, apex acute, in ventral view oblong. Inferior appendage broadest basally, apex digitate, bearing single dorsal spine; in ventral view with basal projection on outer margin and emargination on inner margin. Phallus apex bearing pair of internal apodemes, pair of broad mesolateral membranous lobes, apex divided into pair of lateral lobes.

*Holotype male*: **COSTA RICA:** Cartago, Reserva Tapantí, waterfall, ca. 1km (road) NW tunnel, 9.69°N, 83.76°W, 2-3.viii.1990, el. 1600 m, Holzenthal, Blahnik, and Muñoz (UMSP000201649) (UMSP). *Paratypes*: same data as holotype, 9 males (UMSP); Cartago, Reserva Tapantí, waterfall, ca. 1km (road) NW tunnel, 9.69°N, 83.76°W, 2–3.viii.1990, el. 1600m, Holzenthal, Blahnik, and Muñoz, 1 male (in alcohol) (UMSP); Cartago, Reserva Tapantí, waterfall, ca. 1 km (road) NW tunnel, 9.69°N, 83.76°W, 10.vi.1988, el. 1600m, C.M. and O.S. Flint and R.W. Holzenthal, 1 male (NMNH).

##### Etymology.

Named in honor of R. E. Thomson’s mother, Diane Thomson, who has always been supportive of her daughter’s entomological inclinations.

#### Leucotrichia dinamica

Taxon classificationAnimaliaTrichopteraHydroptilidae

Bueno-Soria, 2010

Fig. 16

dinamica Bueno-Soria, 2010: 23 [Type locality: Mexico, Distrito Federal, Delgación Magdalena-Contreras, Parque “Los Dinamos”; CNIN; male].

##### Diagnosis.

**Figure 16.**
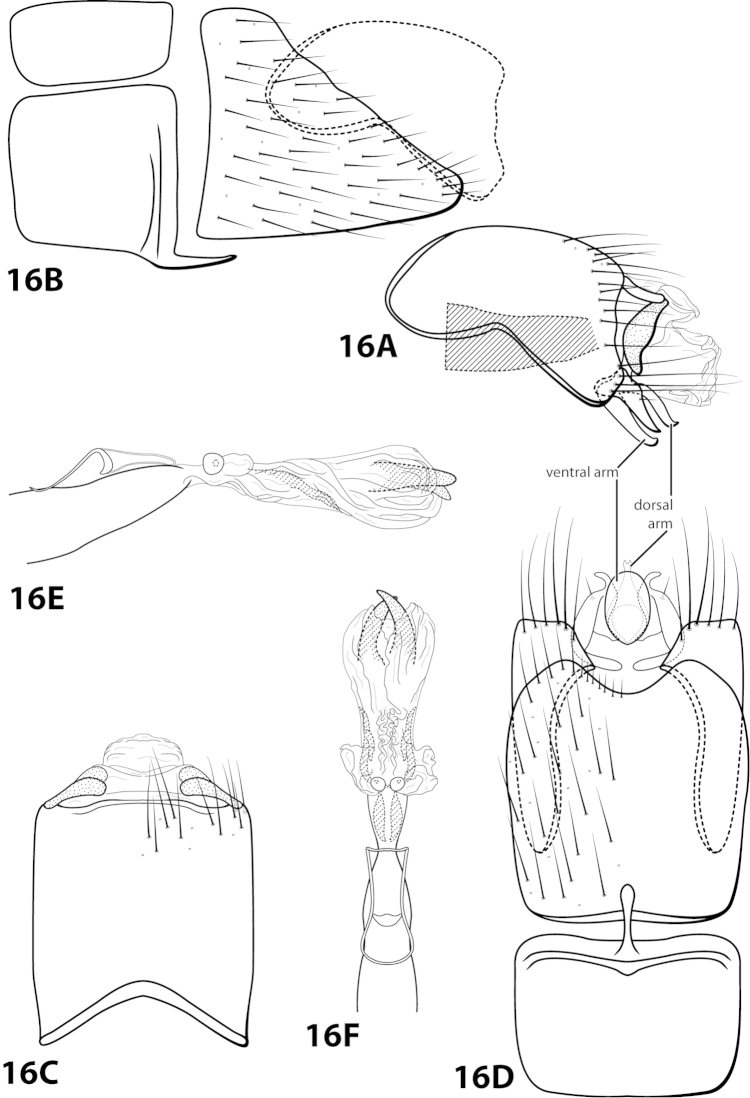
*Leucotrichia dinamica* Bueno-Soria, 2010 (UMSP000140694). Male genitalia: **A** segments IX–X, lateral (base of phallus crosshatched) **B** segments VII–VIII and segment IX margin, lateral **C** segments IX–X, dorsal **D** segments VII–IX, ventral **E** phallus, lateral **F** phallus, dorsal.

*Leucotrichia dinamica* is distinct from all other species in the genus due to the presence of the large scissor-like apical sclerites on the phallus apex (Fig. 16F). The semielliptic sclerite found on the basal loop of the phallus midlength complex is also unique to *Leucotrichia dinamica*.

##### Description.

*Male*. Length of forewing 5.1 mm (n=1). Head unmodified, with 3 ocelli; antennae unmodified. Color in alcohol brown. *Genitalia*. Abdominal sternum VII with acute mesoventral process with apex enlarged in ventral view. Sternum VIII in ventral view with posterior margin concave. Segment IX anterolateral margin produced dorsolaterally, posterolateral margin irregular; in dorsal view anterior margin concave, posterior margin straight. Tergum × with dorsal sclerite simple; ventral sclerite semielliptic with tridentate posterior margin; membranous apex truncate. Subgenital plate with dorsal arm slender, arched mesally, apex slightly hooked dorsad; in ventral view, ovate and produced apically with small emargination; ventral arm digitate, apex slightly hooked dorsad, in ventral view oval. Inferior appendage dorsomesally “humped,” apex acute, bearing single dorsal spine; in ventral view with elongate basal projection strongly bent inward, apex strongly curved outward. Phallus with basal loop with mesal semielliptic sclerite and removed from “windows,” apex bearing pair of elongate internal sclerites and pair of scissor-like apical sclerites.

##### Material examined.

*Holotype male*: **MEXICO: Distrito Federal:** Delegación Magdalena-Contreras, Parque “Los Dinamos,” elev. 3091 m, 29.vi.2007, M. Razo and R. Juarez (in alcohol) (UMSP000140694) (CNIN).

##### Etymology.

Named for Los Dinamos, the location where the holotype was collected.

#### Leucotrichia extraordinaria

Taxon classificationAnimaliaTrichopteraHydroptilidae

Bueno-Soria, Santiago-Fragoso & Barba-Álvarez, 2001

Fig. 17

extraordinaria Bueno-Soria, Santiago-Fragoso & Barba Álvarez, 2001: 145 [Type locality: Mexico, Tabasco, Municipio de Huimanguillo, Arroyo las Flores, Villa de Guadelupe 2^a^ sección Los Chimalapas, km 5 Ruta Malpasito-Carlos A. Madrazo, 17°22'05"N, 93°36'25"W; CNIN; male].

##### Diagnosis.

This species is similar to *Leucotrichia bicornuta*, *Leucotrichia dianeae*, and *Leucotrichia tapantia* sp. n. These species share a similar combination of characteristics present in the phallus and the posterolateral margin of sternum VIII, as discussed under *Leucotrichia bicornuta*. *Leucotrichia extraordinaria* can be easily recognized by the single peg-like seta that occurs on the apex of the posterolateral projection of sternum VIII; the other 3 species all bear long, prominent setae in this location. The posterolateral projection is also much narrower and more acute in *Leucotrichia extraordinaria* than in the other 3 species. Additionally, the concave posterior margin of sternum VIII, when viewed ventrally, is much more rounded in *Leucotrichia extraordinaria* than in *Leucotrichia bicornuta*. It also lacks the small mesal projection present in both *Leucotrichia tapantia* and *Leucotrichia dianeae*.

##### Description.

*Male*. Length of forewing 2.4 mm (n=1). Head unmodified, with 3 ocelli; antennae unmodified. Color in alcohol brown. *Genitalia*. Abdominal sternum VII with digitate mesoventral process. Sternum VIII posteroventral projection bearing single peg-like spine, in ventral view with posterior margin concave. Segment IX anterolateral margin convex, posterolateral margin irregular; in dorsal view anterior margin concave, posterior margin straight. Tergum × with dorsal sclerite simple; ventral sclerite broadest mesally, dorsal and ventral apices knoblike; membranous apex with 2 digitate projections and 1 large subtriangular projection extending posteriad. Subgenital plate with dorsal arm tapering apically, extending dorsad; ventral arm tapering apically, apex slightly hooked dorsad, in ventral view with small apical emargination. Inferior appendage dorsomesally “humped,” bearing single dorsal spine; in ventral view sinuate. Phallus apex bearing multiple pairs of elongate internal sclerites and pair of apical membranous lobes.

##### Material examined.

*Holotype male*: **MEXICO: Tabasco:** Municipio de Huimanguillo, Arroyo las Flores, Villa de Guadalupe 2^a^ sección Los Chimalapas, km 5 Ruta Malpasito-Carlos A. Madrazo, 17°22'05"N 93°36'25"W, 26.vi.1999, J. Bueno and R. Barba (in alcohol) (UMSP000140695) (CNIN).

##### Etymology.

*Extraordinaria*, Spanish indicating “uncommon”, referring to the shape of sternum VIII.

#### Leucotrichia fairchildi

Taxon classificationAnimaliaTrichopteraHydroptilidae

Flint, 1970

Figs 3A , 18

fairchildi Flint, 1970: 10 [Type locality: Panama, Cocle, El Valle; MCZ; male]; 1968: 38 [male, female, Grenada, but misidentified as *sarita*]. — Botosaneanu and Sakal 1992: 201 [biology]. — Flint and Sykora 1993: 54 [Grenada, but misidentified as *sarita*]. — Botosaneanu and Alkins-Koo 1993:7 [larva, case]. — Leucotrichiini, case 2 Botosaneanu and Alkins-Koo 1993: 14 [female]. — Flint 1996: 86 [to synonymy].

##### Diagnosis.

**Figure 18.**
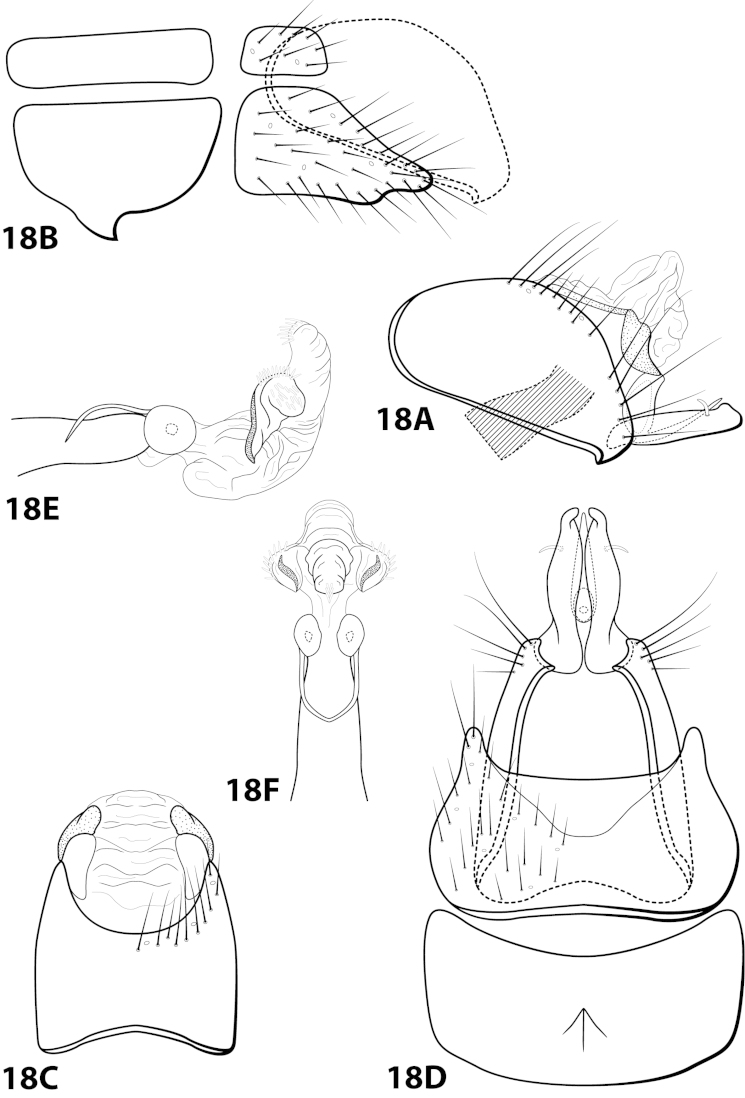
*Leucotrichia fairchildi* Flint, 1970 (UMSP000140357). Male genitalia: **A** segments IX–X, lateral (base of phallus crosshatched) **B** segments VII–VIII and segment IX margin, lateral **C** segments IX–X, dorsal **D** segments VII–IX, ventral **E** phallus, lateral **F** phallus, dorsal.

*Leucotrichia fairchildi* is similar to *Leucotrichia imitator*, *Leucotrichia pictipes*, and *Leucotrichia sarita*; the *pictipes* species group consists of these 4 species. In addition to the similarities discussed under “Species Relationships”, these species also possess similarities in the shape of the inferior appendage and sternum VII, when seen in lateral view, and in the presence of hollow sections of the subgenital plate (Fig. 18A). While the head modifications of *Leucotrichia fairchildi* (Fig. 3A) may be most similar to those of *Leucotrichia pictipes*, the broadened basal flagellum of the antennae and the setiferous protuberance on the dorsum of the head make *Leucotrichia fairchildi* easily distinguishable from *Leucotrichia pictipes* (Fig. 3C). And both the peglike setae present on the phallus apex (Figs 18E, F) and the small mesoventral process on sternum VII in place of prominent setae (Fig. 18B) are characteristics that separate *Leucotrichia fairchildi* from all 3 of the other species.

##### Description.

*Male*. Length of forewing 2.1–3.1 mm (n=53). Head with posterolateral wart large, eversible, with membranous lobe beneath; anteromesally with asymmetrical, setiferous protuberance, with 2 ocelli; antennae with scape enlarged, basal flagellum very broad. Dorsum of head dark brown with light yellow setae; thorax dark brown with light yellow setae dorsally, brown ventrally; leg segments with brown and yellow setae. Forewings covered with fine brown setae with bands of yellow setae basally and scattered patches of yellow setae apically. *Genitalia*. Abdominal sternum VII with short, pointed mesoventral process. Sternum VIII in ventral view with posterior margin concave. Segment IX anterolateral margin produced dorsolaterally, posterolateral margin convex; in dorsal view anterior margin concave, posterior margin concave. Tergum × with dorsal sclerite slender, elongate; ventral sclerite semielliptic with tridentate posterior margin; membranous apex amorphous. Subgenital plate bulbous, with hollow interior; with dorsal arm not apparent; ventral arm slender, acute, extending dorsad, in ventral view lanceolate. Inferior appendage straight, with small apicodorsal projection, bearing single dorsal spine; in ventral view apex hooked inward, broadly rounded on outer margin. Phallus apex trilobed, 2 lateral lobes with darkened dorsal margin, all 3 lobes bearing peglike setae on dorsal surface.

##### Material examined.

*Holotype male*: Holotype deposited at MCZ, but unable to be located. *Nontypes*: **COLOMBIA: Antioquia:** Quebrada La Jiménez, Municipio Sopetrán, elev. 780 m, trap C, 1983-1984, U. Matthias, 2 females (in alcohol) (NMNH); Quebrada La Jiménez, Municipio Sopetrán, elev. 780 m, trap C, 13.vii.1983, U. Matthias, 1 female (in alcohol) (NMNH); Quebrada La Jiménez, Municipio Sopetrán, elev. 780 m, trap C, 22.v.1983, U. Matthias, 3 males, 3 females (in alcohol) (NMNH); **Tolima:** Armero, near Guayabal, 2–10.ii.1977, #58 malaise trap, E.L. Peyton, 3 males (NMNH); Armero, near Guayabal, 2–10.ii.1977, malaise trap, E.L. Peyton, 1 male (in alcohol) (NMNH); **COSTA RICA: Guanacaste:** Parque Nacional Guanacaste, ca. 0.7 km N Est. Maritza, 10.96°N, 85.50°W, 31.viii.1990, el. 550 m, Huisman and Quesada, 3 males (UMSP); Parque Nacional Guanacaste, ca. 0.7 km N Est. Maritza, 10.96°N, 85.50°W, 31.viii.1990, el. 550 m, Huisman and Quesada, 6 females (UMSP); **ECUADOR:** Quevedo, 36 km NE, elev. 1100’, 21.vii.1976, black light, Jeffrey Cohen, 7 males (in alcohol) (NMNH); **EL SALVADOR:** 2 mi. N of Candelaria, 7.viii.1967, O.S. Flint, Jr., 1 male (NMNH); **GRENADA:** 2 mi. E of L. Grand Etang, 4-8.viii.1963, O.S. Flint, Jr., 7 males and 4 females (NMNH); 2 mi. E of L. Grand Etang, 4-8.viii.1963, O.S. Flint, Jr., 7 males, 10 females (in alcohol) (NMNH); St. Andrew, Great R., LaForce Bridges, 12°07.6'N, 61°39.8'W, 19.ix.1996, O.S. Flint, Jr., 2 females (in alcohol) (NMNH); St. John, Concord Valley, 12°07.0'N, 61°44.0'W, 14.ix.1996, O.S. Flint, Jr., 2 females (in alcohol) (NMNH); **PANAMA:** Barro Colorado Island, Snyder-Molino trail, marker 3, light trap III, 10-16.iii.1989, H. Wolda, 1 male (in alcohol) (NMNH); **TOBAGO:** Charlotteville, 5 km S, Hermitage R., 11°19'N, 60°34'W, 10-11.vii.1993, UV light, O.S. Flint, Jr. and N.E. Adams, 1 male (NMNH); Speyside, 1 km NW, Doctor Riv., 11°18'N, 60°32'W, 12.vi.1993, UV light, O.S. Flint, Jr. and N.E. Adams, 1 male (in alcohol) (NMNH); Speyside, 1 km NW, Doctor Riv., 11°18'N, 60°32'W, 12.vi.1993, by net, O.S. Flint, Jr., 4 males (NMNH); St. John, Speyside, 1 km NW, Doctor Riv., 11°18'N, 60°32'W, 12-13.vi.1993, W.N. Mathis, 1 male (NMNH); Parlatuvier West River, 11°18'N, 60°39'W, 14.vi.1993, by net, O.S. Flint, Jr., 1 male, 1 female (NMNH); **TRINIDAD:** Marianne R., 9 km S, Blanchisseuse, 10°46'N, 61°18'W, 25.vi.1993, by net, O.S. Flint, Jr., 4 males (NMNH); Marianne R., 9 km S, Blanchisseuse, 10°46'N, 61°18'W, 28.vi.1993, UV light, O.S. Flint, Jr. and N.E. Adams, 1 male (in alcohol) (NMNH); Paria River, stream, iii.1985, light trap, V. Jones, 7 males (in alcohol) (NMNH); Maracas Falls, elev. 270 m, 10°44'N, 61°24'W, 18.vi.1993, UV light, O.S. Flint, Jr., 1 female (in alcohol) (NMNH); **VENEZUELA: Sucre:** Península de Paria, Puerto Viejo, “Río el Pozo,” 11°43.073'N, 62°28.569'W, elev. 20 m, 3.iv.1995, Holzenthal, Flint, and Cressa, 1 female (NMNH).

##### Etymology.

Named in honor of G. Fairchild, the collector of the holotype specimen.

#### Leucotrichia forrota

Taxon classificationAnimaliaTrichopteraHydroptilidae

Oláh & Johanson, 2011

Fig. 19

forrota Oláh & Johanson, 2011: 160 [Type locality: Peru, San Martín Province, Río Huallaga tributary, small river passing Chazuta, NHRS; male].

##### Diagnosis.

This species is similar to *Leucotrichia inops* and *Leucotrichia riostoumae* sp. n. In all 3 species, the phallus apex is elongate, tubular, curving both ventrad and laterad, and bearing elongate sclerites or apodemes. *Leucotrichia forrota* can be easily distinguished from the other 2 species in having a slender, elongate, fused inferior appendage and a subgenital plate that is fused and continuous with both the ventral and dorsal sclerites of tergum X.

##### Description.

*Male*. Length of forewing 4.6–5.2 mm (n=62). Head with posterolateral warts pronounced, with 3 ocelli; antennae unmodified. Dorsum of head brown with yellow setae; thorax brown with yellow setae dorsally, brown ventrally; leg segments with brown setae. Forewings covered with fine brown setae with stripe of yellow setae running the length of basal 1/2 and scattered patches of yellow setae on apical 1/2. *Genitalia*. Abdominal sternum VII with rugose mesoventral process with apex enlarged in ventral view. Sternum VIII in ventral view with posterior margin concave with pointed mesal emargination. Segment IX anterolateral margin produced mesally, posterolateral margin straight; in dorsal view anterior margin concave, posterior margin concave. Tergum × with dorsal sclerite continuous with ventral sclerite; ventral sclerite with crenulate posterior margin; membranous apex truncate. Subgenital plate fused with ventral sclerite of tergum X; with dorsal arm not apparent; ventral arm slender, digitate, with truncate basal projection, in ventral view oblong with apex rounded. Inferior appendage slender, elongate, apex hooked dorsally, bearing single dorsal spine; in ventral view entirely fused, with basal projection on inner margin, with 2 small apicomesal projections. Phallus apex elongate, coiled, bearing multiple elongate internal sclerites.

##### Material examined.

*Holotype male*: **PERU: San Martín Province:** Río Huallaga tributary, small river passing Chazuta, 6°34.665'S 76°08.209'W, light, loc. 11, 10.i.2009, T. Malm and K.A. Johanson (in alcohol) (NHRS-KAJO 000000329) (NHRS). *Paratypes*: **ECUADOR: Napo:** Tena, 26.v.1977, blacklight, P.J. Spangler and D.R. Givens, 1 male (in alcohol) (NMNH); Tena, 25.v.1977, blacklight, P.J. Spangler and D.R. Givens, 1 male, 1 female (in alcohol) (NMNH); **Pastaza:** Puyo, 3 km N, blacklight, 30.v.1975, Cohen and Langley, 7 males (in alcohol) (NMNH); Puyo, 30.i.1976, blacklight, P.J. Spangler et al., 1 male (in alcohol) (NMNH); Puyo, 3km W, 15.vii.1976, blacklight, J. Cohen, 1 male (in alcohol) (NMNH); Puyo, 5.v.1977, blacklight, P.J. Spangler and D.R. Givens, 4 males (in alcohol) (NMNH); Puyo, 7.v.1977, blacklight, #17, P.J. Spangler and D.R. Givens, 4 males (in alcohol) (NMNH); Puyo, 11.v.1977, blacklight, P.J. Spangler and D.R. Givens, 2 males (in alcohol) (NMNH); Puyo, 13.v.1977, blacklight, P.J. Spangler and D.R. Givens, 2 males (in alcohol) (NMNH); Puyo, 1.5 km S, 14.v.1977, #43, P.J. Spangler and D.R. Givens, 1 male (in alcohol) (NMNH); Puyo, 16.v.1977, blacklight, #51, P.J. Spangler and D.R. Givens, 2 males (in alcohol) (NMNH); Puyo, 2 km N, 30.v.1975, blacklight, Langley and Cohen, 6 males (in alcohol) (NMNH); Puyo, 8-11.ii.1976, blacklight, P.J. Spangler et al., 1 male (in alcohol) (NMNH); Puyo, 15.v.1977, blacklight, #47, P.J. Spangler and D.R. Givens, 1 male, 1 female (in alcohol) (NMNH); Puyo, 30.i.1976, blacklight, P.J. Spangler et al., 2 males (in alcohol) (NMNH); Puyo, 21.v.1977, blacklight, #67, P.J. Spangler and D.R. Givens, 2 males, 1 female (in alcohol) (NMNH). *Nontypes*: **ECUADOR: Napo:** Tena, 4 km S, 26.v.1977, #81, P.J. Spangler and D.R. Givens, 3 males, 11 females (NMNH); Tena, 17 km SW, 28.v.1977, #88, P.J. Spangler and D.R. Givens, 4 males, 6 females (NMNH); Tena, 17 km SW, 26.v.1977, #80, P.J. Spangler and D.R. Givens, 1 male (NMNH); **Pastaza:** Puyo, 1.5 km S, 14.v.1977, #43, P.J. Spangler and D.R. Givens, 5 females (NMNH); Puyo, 9.v.1975, blacklight, #29, P.J. Spangler and D.R. Givens, 1 male (NMNH); Puyo, 3 km N, 30.v.1975, Cohen and Langley, 9 males, 6 females (NMNH); Puyo, 6.v.1977, blacklight, P.J. Spangler and D.R. Givens, 4 males (in alcohol) (NMNH); **PERU:** Cuzco, Pilcopata, elev. 600 m, premontane moist forest, 11-14.xii.1979, J.B. Heppner, 1 male, 1 female (in alcohol) (NMNH).

##### Etymology.

*Forrott*, Hungarian for “fused”, referring to the completely fused inferior appendage.

#### Leucotrichia fulminea

Taxon classificationAnimaliaTrichopteraHydroptilidae

Thomson & Holzenthal sp. n.

http://zoobank.org/BA5F712C-87B9-4EE7-B693-299A95B9844B

Fig. 20

##### Diagnosis.

*Leucotrichia fulminea* sp. n., is most similar to *Leucotrichia angelinae*. Both species possess a long, slender mesoventral process on sternum VII; a broad, rounded inferior appendage; a subgenital plate with a small apical emargination in the ventral arm when viewed ventrally; and a phallus bearing a pair of subapicodorsal sclerites that each point laterad. *Leucotrichia fulminea* can be distinguished by the rugose ventral surface of the mesoventral process and the 3 small setae on the inferior appendage, each of which is absent in *Leucotrichia angelinae*. Near the base of the subapicodorsal sclerite on the phallus there is also a bend that can be seen in the lateral view that is not present in *Leucotrichia fulminea*.

##### Description.

*Male*. Length of forewing 4.7–4.9 mm (n=7). Head unmodified, with 3 ocelli; antennae unmodified. Dorsum of head dark brown with light yellow and dark brown setae; thorax dark brown with dark brown and light yellow setae dorsally, brown ventrally; leg segments with brown setae. Forewings covered with fine light yellow setae mesally with dark brown setae along the edges. *Genitalia*. Abdominal sternum VII with elongate, rugose mesoventral process. Sternum VIII in ventral view with posterior margin concave. Segment IX anterolateral margin convex, posterolateral margin irregular; in dorsal view anterior margin shallowly concave, posterior margin irregular. Tergum × with dorsal sclerite slender, bent ventrad; ventral sclerite large, semielliptic, with tridentate posterior margin; membranous apex with dorsal and ventral lobes, rhomboid in dorsal view. Subgenital plate with dorsal arm not apparent; ventral arm rounded basally, arched mesally, tapering apically, in ventral view with rounded apical emargination. Inferior appendage apex rounded, bearing 3 small dorsal setae; in ventral view inner margin crenulate. Phallus apex bearing multiple internal apodemes and pair of pointed subapicodorsal sclerites extending outward.

*Holotype male*: **ECUADOR:** Cañar, Río Chauchas, 2910m, 3 km N Zhud, 17.ix.1990, O.S. Flint (UMSP000140611) (NMNH). *Paratypes*: same data as holotype, 5 males (NMNH); same data as holotype, 1 male (UMSP).

##### Etymology.

*Fulmineus*, Latin for “of lightning”, referring to the shape of the pointed dorsal sclerites on the phallus, which resemble lightning bolts when viewed laterally.

#### Leucotrichia gomezi

Taxon classificationAnimaliaTrichopteraHydroptilidae

Flint, 1970

Fig. 21

gomezi Flint, 1970: 7 [Type locality: Dominican Republic, La Palma, 12 km E. El Río; NMNH; male; larva, case].

##### Diagnosis.

This species is most similar to *Leucotrichia tubifex*; in both species, sternum VIII is subquadrate in lateral view and the mesoventral process of segment VII is simple and pointed. The shape of the inferior appendage in each species is similar, with a small mesodorsal spine on the dorsal margin when viewed laterally and apex digitate when viewed ventrally. *Leucotrichia gomezi* differs from *Leucotrichia tubifex* in a combination of traits present on the phallus: the presence of large apical lobes, the large dorsal sclerite, and the basal supports of the midlength complex basal loop, none of which are present in *Leucotrichia tubifex*. Additional similarities and differences are discussed under *Leucotrichia tubifex*.

##### Description.

**Figure 21.**
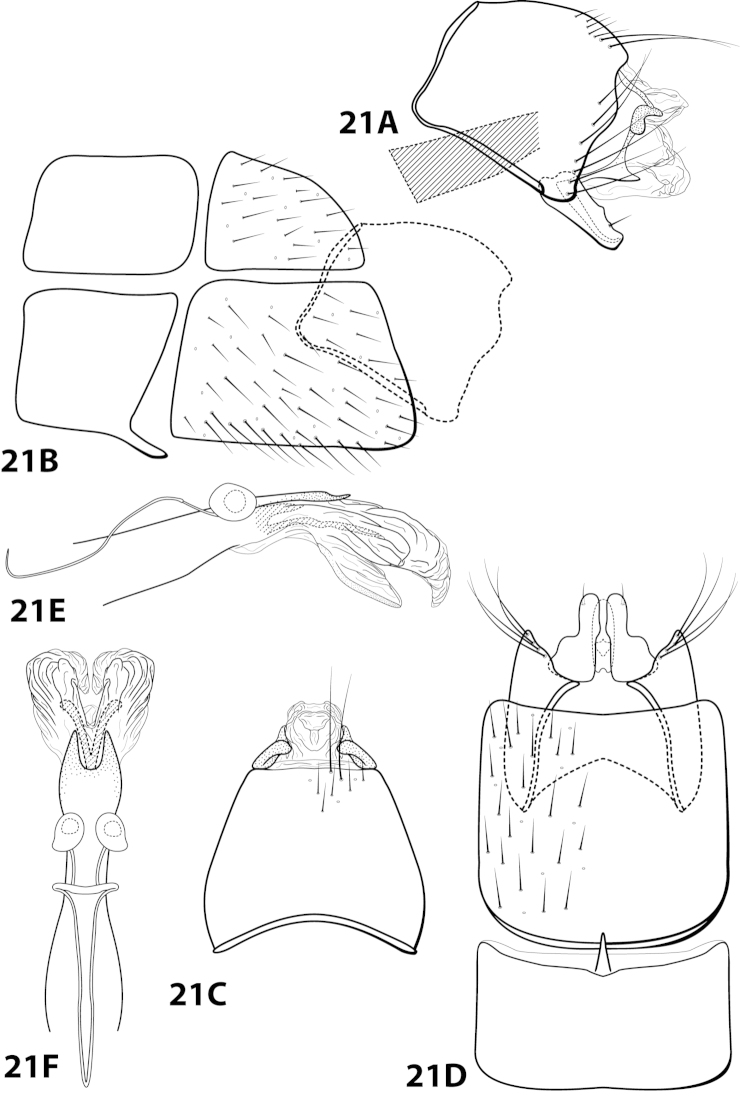
*Leucotrichia gomezi* Flint, 1970 (USNM70897). Male genitalia: **A** segments IX–X, lateral (base of phallus crosshatched) **B** segments VII–VIII and segment IX margin, lateral **C** segments IX–X, dorsal **D** segments VII–IX, ventral **E** phallus, lateral **F** phallus, dorsal.

*Male*. Length of forewing 4.3–5.0 mm (n=15). Head unmodified, with 3 ocelli; antennae unmodified. Dorsum of head brown with light brown setae; thorax brown with bronze setae dorsally, brown ventrally; leg segments with brown setae. Forewings covered with fine bronze setae. *Genitalia*. Abdominal sternum VII with acute mesoventral process. Sternum VIII in ventral view with posterior margin very shallowly concave. Segment IX anterolateral margin produced mesally, posterolateral margin irregular; in dorsal view anterior margin concave, posterior margin straight. Tergum × with dorsal sclerite slender, elongate; ventral sclerite small, reniform; membranous apex with small dorsal lobe and larger, subtriangular ventral lobe. Subgenital plate with dorsal arm triangular; ventral arm slender, apex slightly hooked dorsad, laterally obscured from view by inferior appendage, in ventral view oblong with small basal emargination and apex rounded. Inferior appendage broadest basally, with small dorsomesal spine, apex truncate, bearing single dorsal spine; in ventral view basally subquadrate, apically digitate. Phallus with median complex bearing short basal supports; apex bearing large, flat dorsal sclerite with large, rounded, apical emargination; paired elongate internal sclerites; 2 large, membranous dorsal lobes with apices curving ventrad; 1 smaller, mesoventral lobe (Fig. 21E).

##### Material examined.

*Holotype male*: **DOMINICAN REPUBLIC:** La Palma, 12 km E of El Río, 2-13.vi.1969, Flint and Gomez (USNM 70897) (NMNH). *Paratypes*: same data as holotype, 14 males, 1 female (NMNH); **DOMINICAN REPUBLIC:** Convento, 12 km S of Constanza, 6-13.vi.1969, Flint and Gomez, 2 females (NMNH).

##### Etymology.

Named after Gomez, one of the collectors of the holotype specimen.

#### Leucotrichia hispida

Taxon classificationAnimaliaTrichopteraHydroptilidae

Thomson & Holzenthal sp. n.

http://zoobank.org/6AAB8D7C-2B97-4AFB-BB36-180CDDCDA557

Fig. 22

##### Diagnosis.

*Leucotrichia hispida* sp. n., is most similar to *Leucotrichia botosaneanui*, *Leucotrichia chiriquiensis*, *Leucotrichia limpia*, and *Leucotrichia viridis*. These species share a similar combination of characteristics present in the phallus and the posterolateral margin of sternum VIII, as discussed under *Leucotrichia botoeaneanui*. *Leucotrichia hispida* is distinct from the other 4 species in having an extremely rugose apex on the mesoventral process of sternum VII, a prominent tuft of setae on the posteroventral projection of sternum VIII, and no external spines or sclerites on the phallus apex.

##### Description.

**Figure 22.**
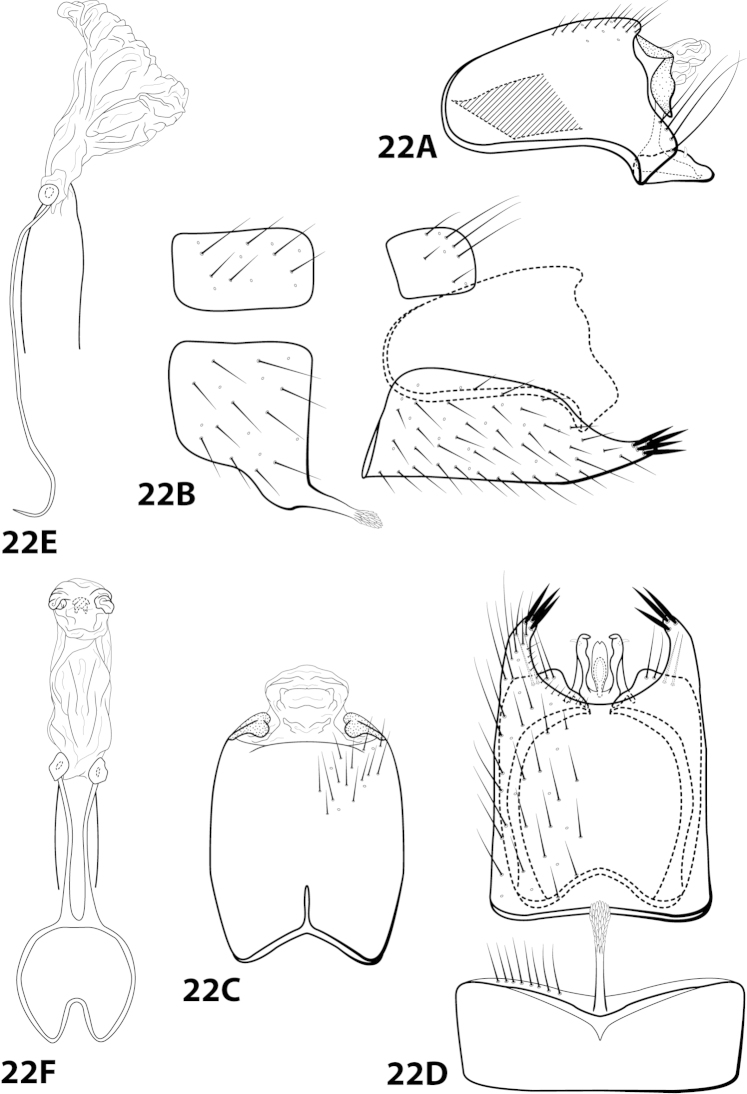
*Leucotrichia hispida* sp. n. (UMSP000140610). Male genitalia: **A** segments IX–X, lateral (base of phallus crosshatched) **B** segments VII–VIII and segment IX margin, lateral **C** segments IX–X, dorsal **D** segments VII–IX, ventral **E** phallus, lateral **F** phallus, dorsal.

*Male*. Length of forewing 4.2–5.0 mm (n=3). Head unmodified, with 3 ocelli; antennae unmodified. Dorsum of head dark brown with light yellow and dark brown setae; thorax dark brown with light yellow setae dorsally, brown ventrally; leg segments with brown setae. Forewings covered with fine dark brown setae with scattered patches of light yellow setae. *Genitalia*. Abdominal sternum VII with large, rugose mesoventral process and row of prominent setae (Fig. 22D). Sternum VIII posteroventral projection bearing stout, prominent setae, in ventral view with posterior margin concave. Segment IX anterolateral margin broadly produced dorsolaterally, posterolateral margin irregular; in dorsal view anterior margin concave, posterior margin straight. Tergum × with dorsal sclerite simple; ventral sclerite semielliptic with tridentate posterior margin; membranous apex small, suborbicular. Subgenital plate with dorsal arm not apparent; ventral arm broadest basally, apex slightly hooked dorsad, in ventral view oblong with small apical emargination. Inferior appendage broadest basally, apex digitate, bearing single dorsal spine; in ventral view with pair of digitate basal projections, inner margin crenulate. Phallus with median complex bearing elongate basal supports; apex bearing ventral “bulge” (Fig. 22E), pair of small apicodorsal lobes, and small sclerotized internal structure between apical lobes (Fig. 22F).

*Holotype male*: **COSTA RICA:** San José, Río Savegre, 9°33.9'N, 83°48'W, 2270 m, 7-8.viii.2001, D. and W.N. Mathis (UMSP000140610) (NMNH). *Paratypes*: same data as holotype, 1 male (NMNH), 1 male (UMSP).

##### Etymology.

*Hispidus*, Latin for “bristly”, referring to the extremely rugose apex of the mesoventral process of sternum VII.

#### Leucotrichia imitator

Taxon classificationAnimaliaTrichopteraHydroptilidae

Flint, 1970

Figs 4B , 23

imitator Flint, 1970: 8 [Type locality: Mexico, Vera Cruz, Plan del Río Ver, Rt. 140, km 368; NMNH; male; larva, case].

##### Diagnosis.

**Figure 23.**
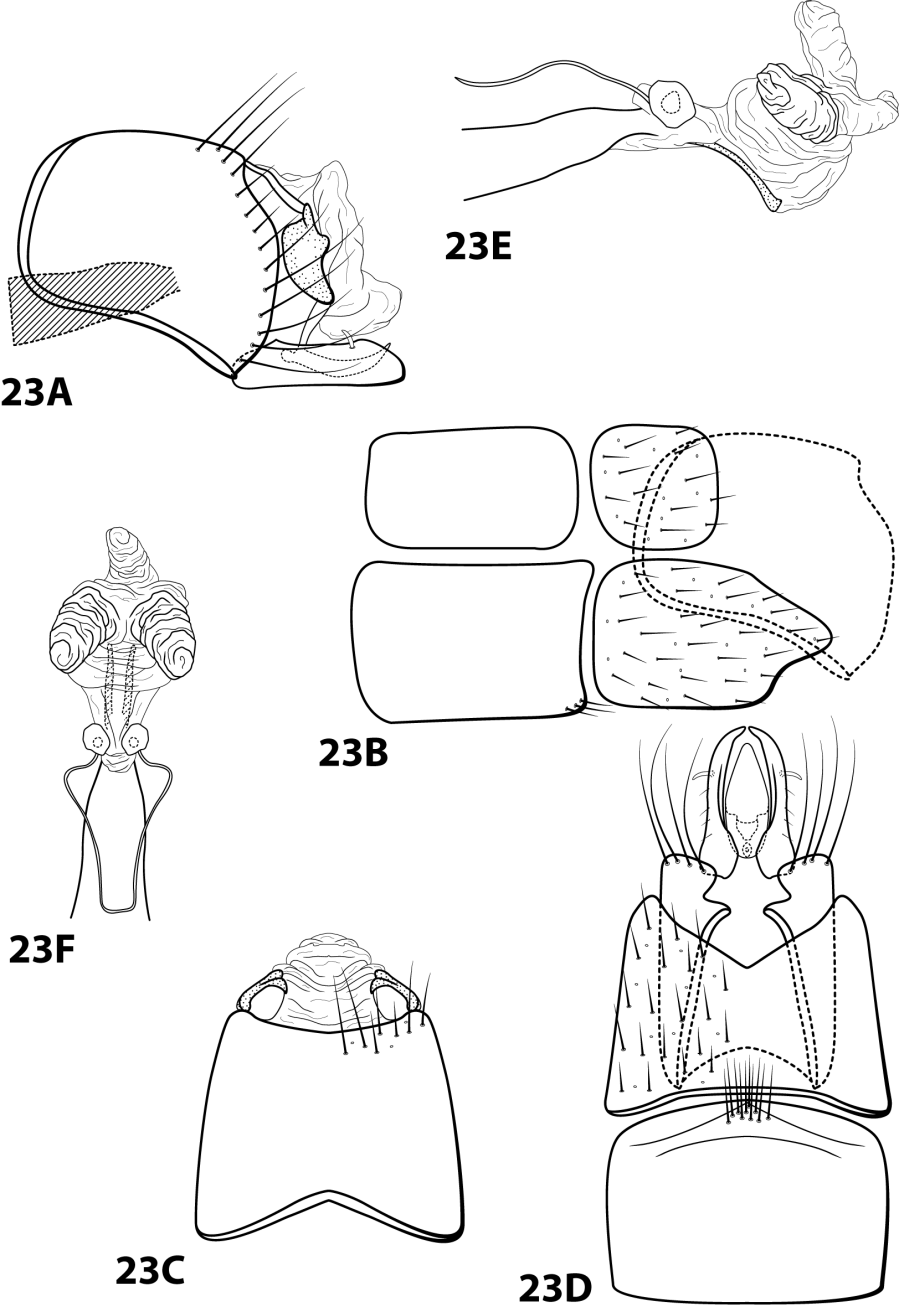
*Leucotrichia imitator* Flint, 1970 (USNM70898). Male genitalia: **A** segments IX–X, lateral (base of phallus crosshatched) **B** segments VII–VIII and segment IX margin, lateral **C** segments IX–X, dorsal **D** segments VII–IX, ventral **E** phallus, lateral **F** phallus, dorsal.

*Leucotrichia imitator* is similar to *Leucotrichia fairchildi*, *Leucotrichia pictipes*, and *Leucotrichia sarita*. *Leucotrichia imitator* is most similar to *Leucotrichia pictipes* in having a phallus with multiple membranous, asetiferous lobes arising from the apex (Fig. 23E, F). *Leucotrichia imitator* can be distinguished by having 3 lobes arising from the same apical location, while *Leucotrichia pictipes* has 2 pairs of symmetrically arranged lobes. *Leucotrichia imitator* is unique in that it has the reduced number of ocelli in male specimens, but does not also have some degree of head modifications (Fig. 4B).

##### Description.

*Male*. Length of forewing 3.1–3.9 mm (n=22). Head unmodified, with 2 ocelli; antennae unmodified. Dorsum of head brown with light brown setae; thorax dark brown with light brown setae dorsally, light brown ventrally; leg segments with light brown setae. Forewings covered with fine dark brown setae with scattered bands of light yellow setae. *Genitalia*. Abdominal sternum VII with mesoventral process replaced by tuft of dense, prominent setae. Sternum VIII in ventral view with posterior margin with V-shaped concavity. Segment IX anterolateral margin broadly produced dorsolaterally, posterolateral margin convex; in dorsal view anterior margin concave, posterior margin broadly concave. Tergum × with dorsal sclerite slender, elongate; ventral sclerite semielliptic with tridentate posterior margin; membranous apex not well developed. Subgenital plate with dorsal arm not apparent; ventral arm with small knoblike basal projection, tapering apically, curved dorsad, in ventral view lanceolate. Inferior appendage straight, with small pointed subdorsobasal projection, bearing single dorsal spine; in ventral view basally subquadrate, apex hooked inward. Phallus apex bearing pair of internal sclerites and 3 digitate apical lobes.

##### Material examined.

*Holotype male*: **MEXICO: Veracruz:** Plan del Río Ver, Rt. 140 km 368, 23.vii.1965, Flint and Ortiz (USNM 70898) (NMNH). *Paratypes*: Same data as holotype, 2 males (NMNH); **COSTA RICA: Puntarenas:** Río Seco, NW of Esparta, 23.vii.1967, O.S. Flint, Jr., 2 males (in alcohol) (NMNH); **GUATEMALA: El Progreso:** San Agustín Acasaguastlán, 11-21.viii.1965, Flint and Ortiz, 2 males, 1 female (NMNH); **Suchitepéquez:** Puente Ixtacapa, near San Antonio, 18-19.vi.1966, Flint and Ortiz, 1 male (NMNH). *Nontypes*: **COSTA RICA: Puntarenas:** Quebrada Portera nr Portera Grande, 5.vii.1992, T. Shepard, 13 males (in alcohol) (UMSP); **MEXICO: Sonora:** Yaqui River, Hwy 16, near Tonichi, 19.viii.1986, Baumann, Sargent, and Kondratieff, 1 male, 1 female (in alcohol) (NMNH).

##### Etymology.

Unknown.

#### Leucotrichia inflaticornis

Taxon classificationAnimaliaTrichopteraHydroptilidae

Botosaneanu, 1993

Figs 3B , 24

inflaticornis Botosaneanu, in Botosaneanu and Alkins-Koo 1993: 10 [Type locality: Trinidad, 2nd. order stream at “La Laja”, catchment of Rio Guanapo; ZMUA; male; larva, case]. — Botosaneanu and Sakal 1992: 201 [biology].

##### Diagnosis.

*Leucotrichia inflaticornis* can be separated from other species in the genus by the inflated basal segments of the antennae (Fig. 3B) for which it was named, and the lack of mesoventral process on sternum VII. Additionally, the phallus apex bears 4 pairs of distinct dark spines.

##### Description.

Holotype pharate adult, abdomen missing, genitalia redescribed from Botosaneanu and Alkins-Koo (1993). *Male*. Length of forewing 1.2 mm (n=1). Head unmodified, with 3 ocelli; antennae scape normal, next 5–6 basal flagellum broadened similar to that of *Leucotrichia pictipes*. Color in alcohol brown. *Genitalia*. Abdominal sternum VII with mesoventral process not apparent. Sternum VIII in ventral view with posterior margin with V-shaped concavity. In dorsal view anterior margin of segment IX convex, posterior margin concave. Membranous apex of tergum × suborbicular. Subgenital plate apically bilobed, with small pointed projection at base of each lobe. Inferior appendage apex truncate; in ventral view entirely fused. Phallus with median complex present but only slightly developed, apex bearing 4 pairs of symmetrically arranged stout, dark spines.

##### Material examined.

*Holotype male*: **TRINIDAD:** 2nd order stream at “La Laja”, catchment of Rio Guanapo (in alcohol) (UMSP000140327) (ZMUA).

##### Etymology.

Specific epithet refers to the inflated appearance of basal antennal segments.

#### Leucotrichia inops

Taxon classificationAnimaliaTrichopteraHydroptilidae

Flint, 1991

Fig. 25

inops Flint, 1991: 43 [Type locality: Colombia, Dpto. Antioquia, 12 km E Medellín, road to Sta. Elena; NMNH; male].

##### Diagnosis.

This species is similar to *Leucotrichia forrota* and *Leucotrichia riostoumae* sp. n. These species share a similar combination of characteristics present in the phallus, as discussed under *Leucotrichia forrota*. Of these species, *Leucotrichia inops* is most similar to *Leucotrichia riostoumae*. Each of these species possess a convex posterolateral margin on sternum VIII; an anterolateral projection on segment IX; an unfused inferior appendage that is basally subquadrate, apically hooked, and lacks a dorsal spine; and an apparent lack of midlength complex on the phallus. *Leucotrichia inops* can be distinguished by the small tuft of apical setae on the phallus and the shape of the membranous apex of tergum X, which is larger and extends further posteriad than that of *Leucotrichia riostoumae*. Also, in *Leucotrichia inops* the concave posterior margin of sternum VIII is V-shaped, while it is shallowly rounded in *Leucotrichia riostoumae*.

##### Description.

*Male*. Length of forewing 3.5–4.0 mm (n=3). Head unmodified, with 3 ocelli; antennae unmodified. Dorsum of head dark brown with light gray-green setae on anterior edge; thorax brown with dark brown and light gray-green setae dorsally, brown ventrally; leg segments with brown setae. Forewings covered with fine mottled light gray-green setae with dark brown setae on apical 1/3. *Genitalia*. Abdominal sternum VII with acute mesoventral process with small basal ridge, apex enlarged in ventral view. Sternum VIII in ventral view with posterior margin concave. Segment IX anterolateral margin with curved ventrolateral projection, posterolateral margin irregular; in dorsal view anterior margin concave, posterior margin concave. Tergum × with dorsal sclerite continuous with ventral sclerite; ventral sclerite semielliptic with crenulate posterior margin; membranous apex subtriangular. Subgenital plate with dorsal arm digitate; ventral arm acute, extending dorsad, in ventral view oblong. Inferior appendage basally subquadrate, apex hooked dorsally, without dorsal spine; in ventral view subtriangular, outer margin crenulate. Phallus with median complex not apparent; apex elongate, curved strongly both ventrad and posteriad; with very slender, elongate sclerites; apex bearing tuft of small spines.

##### Material examined.

*Holotype male*: **COLOMBIA: Antioquia:** 12 km E Medellín, rd. to Santa Elena, 6.ii.1983, O.S. Flint, Jr. (USNM 104530) (NMNH). *Paratypes*: **COLOMBIA: Antioquia:** Quebrada La Iguaná, 17 km NW Medellín, rd. to San Jerónimo, 14-15.ii.1983, O.S. Flint, Jr., 1 male (NMNH); Quebrada La Iguaná, 17km NW Medellín, rd. to San Jerónimo, 22.ii.1984, C.M. and O.S. Flint, Jr., 1 female (NMNH). *Nontypes*: **ECUADOR: Pichincha:** 2.3 km S Tandayapa, elev. 1800 m, 6.ix.1990, O.S. Flint, Jr., 1 male (NMNH).

##### Etymology.

Unknown.

#### Leucotrichia interrupta

Taxon classificationAnimaliaTrichopteraHydroptilidae

Flint, 1991

Fig. 26

interrupta Flint, 1991: 41 [Type locality: Colombia, Dpto. Antioquia, Quebrada Espadera, 7 km E Medellín, on road to Sta. Elena; NMNH; male].

##### Diagnosis.

*Leucotrichia interrupta* can be separated from all other species in the genus by the unique form of the phallus apex, which is trilobed and has the 2 lateral lobes appearing more bulbous than lobe-like. A second characteristic unique to *Leucotrichia interrupta* is the slender sclerite arising from the base of the “windows” of the midlength complex and following the anterior edge of each of the lateral lobes. The inferior appendage is also distinct in its large, subtriangular shape when viewed laterally.

##### Description.

**Figure 26.**
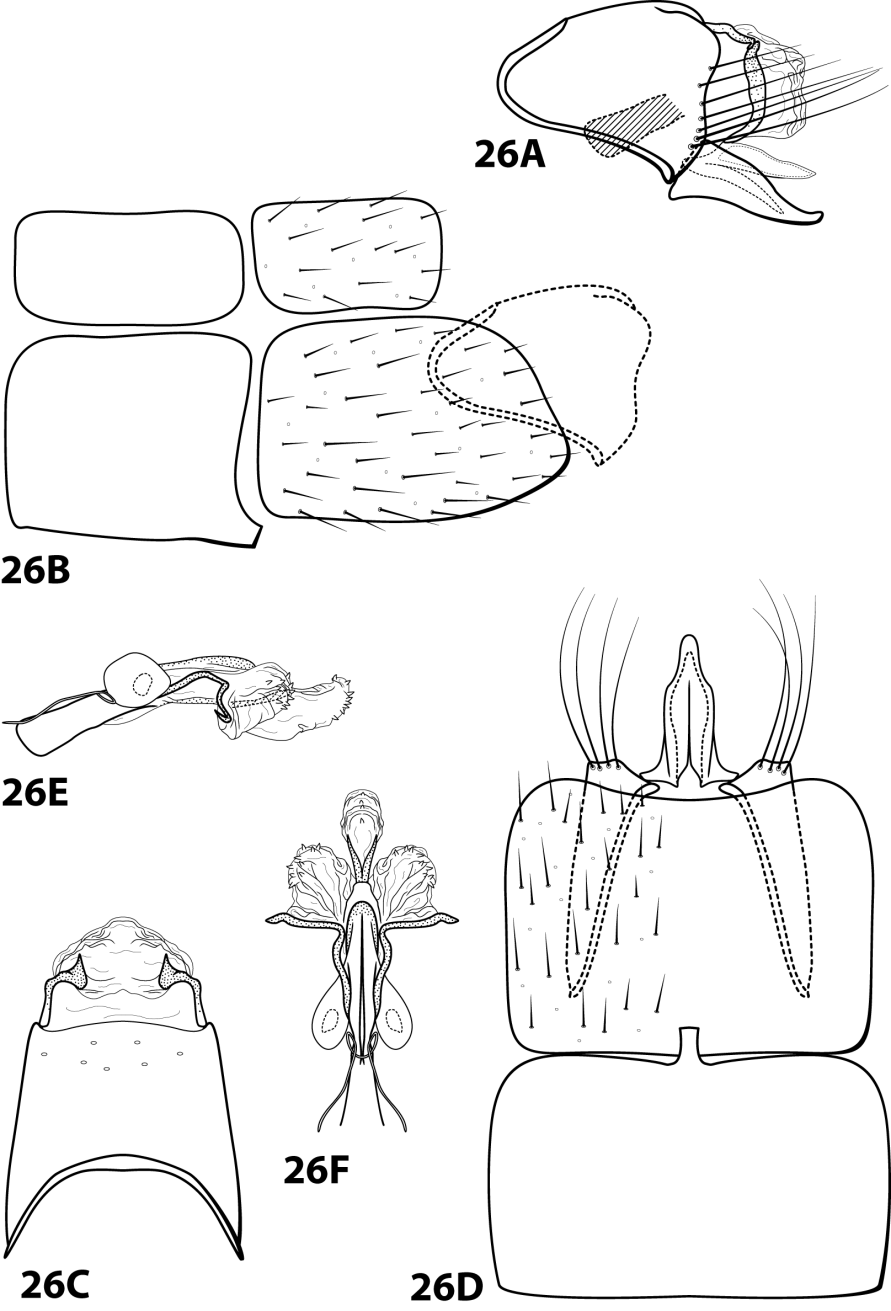
*Leucotrichia interrupta* Flint, 1991 (USNM104528). Male genitalia: **A** segments IX–X, lateral (base of phallus crosshatched) **B** segments VII–VIII and segment IX margin, lateral **C** segments IX–X, dorsal **D** segments VII–IX, ventral **E** phallus, lateral **F** phallus, dorsal.

*Male*. Length of forewing 3.5 mm (n=1). Head unmodified, with 3 ocelli; antennae unmodified. Dorsum of head brown with yellow setae; thorax dark brown with bright yellow setae dorsally, brown ventrally; leg segments with brown setae. Forewings covered with fine yellow setae, apical 1/4 with dark brown setae. *Genitalia*. Abdominal sternum VII with mesoventral process broken (indicated in Fig. 26D). Sternum VIII in ventral view with posterior margin very shallowly concave. Segment IX anterolateral margin produced mesally, posterolateral margin irregular; in dorsal view anterior margin concave, posterior margin straight. Tergum × with dorsal sclerite simple; ventral sclerite simple; membranous apex truncate. Subgenital plate with dorsal arm hollow, triangular (Fig. 26A); ventral arm slender, tapering apically, curved ventrad, in ventral view subovate. Inferior appendage subtriangular, without dorsal spine; in ventral view entirely fused, apically digitate. Phallus apex trilobed; lateral lobes bulbous and with slender, elongate, sinuate sclerite along outer edge; short spines present on dorsal surface of all 3 lobes.

##### Material examined.

*Holotype male*: **COLOMBIA: Antioquia:** Quebrada Espadera, 7 km E Medellín, rd. to Santa Elena, 24.ii.1983, O.S. Flint, Jr. (USNM 104528) (NMNH). *Paratypes*: Same data as holotype, 1 female (NMNH).

##### Etymology.

Unknown.

#### Leucotrichia kateae

Taxon classificationAnimaliaTrichopteraHydroptilidae

Thomson & Holzenthal sp. n.

http://zoobank.org/F72310F9-3255-4D60-9EB8-60D62393A2D5

Fig. 27

##### Diagnosis.

*Leucotrichia kateae* sp. n., is known only from the male holotype. This species is most similar to *Leucotrichia tritoven*. Similarities between these species in the inferior appendage and the phallus are discussed under *Leucotrichia tritoven*. *Leucotrichia kateae* can be easily recognized from *Leucotrichia tritoven* by the mesoventral process of sternum VII, which is noticeably much larger and has a rugose ventral surface. The inferior appendage of *Leucotrichia kateae* is longer than that of *Leucotrichia tritoven* when viewed ventrally, and has a crenulate margin that is lacking in *Leucotrichia tritoven*. *Leucotrichia kateae* also bears a pair of small, curved, pointed sclerites on the phallus that are lacking in *Leucotrichia tritoven*.

##### Description.

*Male*. Length of forewing 2.4 mm (n=1). Head missing. Thorax brown with light brown setae dorsally, brown ventrally; leg segments with brown setae. Forewings covered with fine light yellow setae, inner edge with longitudinal stripe of brown setae. *Genitalia*. Abdominal sternum VII with elongate, rugose mesoventral process. Sternum VIII in ventral view with posterior margin concave. Segment IX anterolateral margin slightly produced ventrolaterally, posterolateral margin irregular; in dorsal view anterior margin shallowly concave, posterior margin concave. Tergum × with dorsal sclerite slender; ventral sclerite with rounded projection on posterior margin; membranous apex not well developed. Subgenital plate with dorsal arm broadest basally, tapering apically; ventral arm slender, digitate, in ventral view sublanceolate. Inferior appendage dorsomesally “humped,” apex truncate, bearing single dorsal spine; in ventral view inner and outer margins crenulate. Phallus apex bearing multiple obscured internal structures; small pair of pointed, curved, mesal sclerites; pair of mildly sinuate, subapicolateral sclerites; pair of apicodorsal membranous lobes.

*Holotype male*: **VENEZUELA: Aragua:** 1 km E Estacíon Biológica Rancho Grande, 10.352°N67.680°W, el. 1100 m, 27.i.1994, Holzenthal, Cressa, and Rincón, 1 male (UMSP000201690) (UMSP).

##### Etymology.

Named in honor of R. E. Thomson’s sister, Kate Thomson, who had to put up with a sister who loves bugs.

#### Leucotrichia laposka

Taxon classificationAnimaliaTrichopteraHydroptilidae

Oláh & Johanson, 2011

Fig. 28

laposka Oláh & Johanson, 2011: 162 [Type locality: Peru, San Martín Province, creek crossing road Juan Guerra-Chazuta, 14 km (rd.) E Colombia Bridge, NHRS; male].

##### Diagnosis.

*Leucotrichia laposka* can be easily separated from all other species in the genus by the apparent absence of the entire subgenital plate. The trilobed apex of the phallus is also proportionately much larger in this species than in any other in the genus. The rows of elongate spines along the mesal lobe are also unique to *Leucotrichia laposka* and, when viewed laterally, suggest the appearance of the spur of a cowboy boot.

##### Description.

*Male*. Length of forewing 2.4–2.8 mm (n=5). Head with posterolateral warts pronounced, originally described as “hinged” but everscibility was not apparent, with 3 ocelli; antennae unmodified. Color in alcohol brown. *Genitalia*. Abdominal sternum VII with slender, acute mesoventral process. Sternum VIII in ventral view with posterior margin concave. Segment IX anterolateral margin convex, posterolateral margin irregular with small knob-like structure mesally; in dorsal view anterior margin concave, posterior margin concave. Tergum × with dorsal sclerite bent ventrad, continuous with ventral sclerite; ventral sclerite bent dorsad; membranous apex with large, membranous, apicoventrally hooked projection. Subgenital plate not apparent. Inferior appendage flat, elongate, dorsal margin crenulate, without dorsal spine but sparsely setose; in ventral view fused basally, subquadrate. Phallus apex large, trilobed; mesal lobe with rows of large, elongate spines.

##### Material examined.

*Holotype male*: **PERU: San Martín Province:** creek crossing road Juan Guerra-Chazuta, 14 km (rd.) E Colombia Bridge, 6°35.594'S 76°13.172'W, light, loc. 09, 9.i.2009, T. Malm and K.A. Johanson (in alcohol) (NHRS-KAJO 000000328) (NHRS). *Paratypes*: **PERU: San Martín Province:** La Catarata de Ahuashiyascu, 6°27.544'S 76°18.192'W, light, loc. 07, 7.i.2009, T. Malm and K.A. Johanson, 2 males (in alcohol) (NHRS), 2 males (in alcohol) (OPC).

##### Etymology.

*Laposka*, Hungarian for diminutive form of “flat”, referring to the broadened inferior appendage.

#### Leucotrichia lerma

Taxon classificationAnimaliaTrichopteraHydroptilidae

Angrisano & Burgos, 2002

Fig. 29

lerma Angrisano & Burgos, 2002: 106 [Type locality: Argentina, Salta, Río Lesser, 18 km NW Salta; IML; male].

##### Diagnosis.

This species is most similar to *Leucotrichia brochophora*, *Leucotrichia padera*, and *Leucotrichia rhomba* sp. n. These species share a similar form in the apex of the phallus, sternum VIII, and segment IX, as discussed under *Leucotrichia brochophora*. *Leucotrichia lerma* differs from these species in the hooked appearance of the lateral sclerite of tergum × and the subtriangular appearance of the inferior appendage in ventral view.

##### Description.

Redescribed from Angrisano and Burgos (2002). *Male*. Length of forewing 2.9 mm (n=1). Head unmodified, with 3 ocelli; antennae unmodified. *Genitalia*. Abdominal sternum VII with slender, elongate mesoventral process. Sternum VIII in ventral view with posterior margin concave with row of prominent setae. Segment IX anterolateral margin produced mesally, posterolateral margin convex. Tergum × with lateral sclerite with apicoventral hook on posterior margin. Membranous apex of tergum × amorphous. Subgenital plate with dorsal arm not apparent; ventral arm tapering apically, apex slightly hooked dorsad. Inferior appendage digitate, apex hooked dorsally, bearing single dorsal spine; in ventral view subtriangular. Phallus apex bearing pair of ventral sclerites.

##### Material examined.

*Holotype male*: Holotype deposited at IML, but could not be obtained.

##### Etymology.

Named for Valle de Lerma, the location where the holotype was collected.

#### Leucotrichia limpia

Taxon classificationAnimaliaTrichopteraHydroptilidae

Ross, 1944

Fig. 30

limpia Ross, 1944: 273 [Type locality: United States, Texas, Fort Davis, Limpia Creek; INHS; male.] — Flint 1970: 6 [male]; 1996: 86 [correction of errors in 1970 paper].

##### Diagnosis.

**Figure 30.**
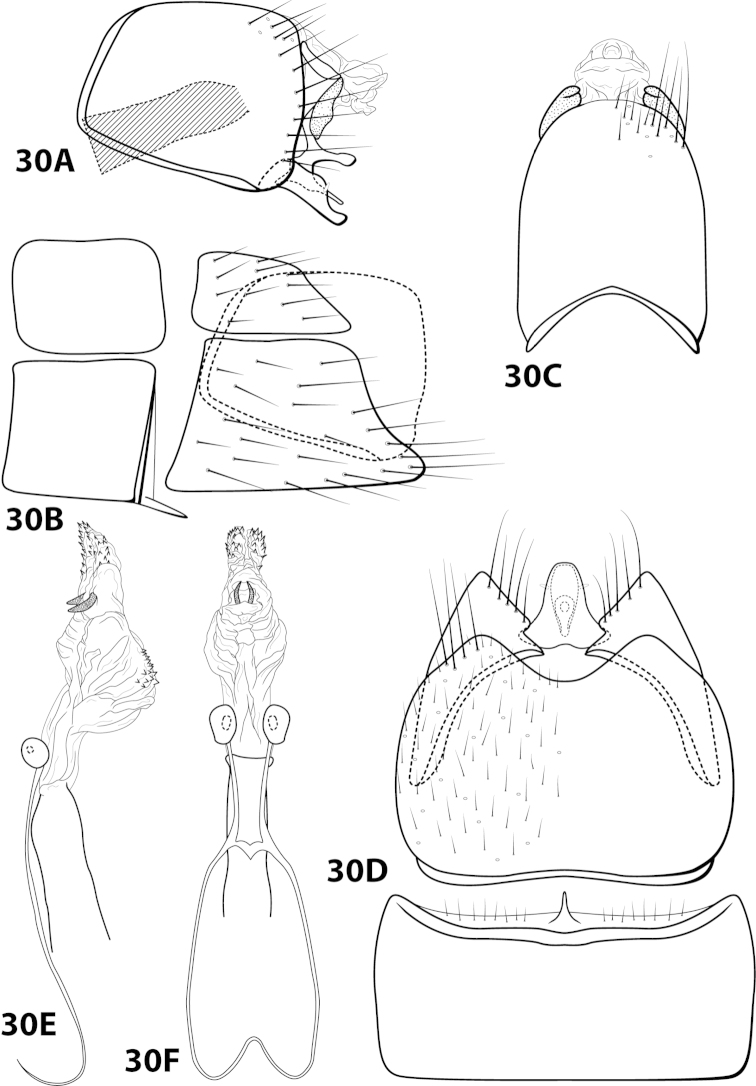
*Leucotrichia limpia* Ross, 1944 (INHS22335). Male genitalia: **A** segments IX–X, lateral (base of phallus crosshatched) **B** segments VII–VIII and segment IX margin, lateral **C** segments IX–X, dorsal **D** segments VII–IX, ventral **E** phallus, lateral **F** phallus, dorsal.

*Leucotrichia limpia* is most similar to *Leucotrichia botosaneanui*, *Leucotrichia chiriquiensis*, *Leucotrichia hispida*, and *Leucotrichia viridis*. These species share a similar combination of characteristics present in the phallus and the posterolateral margin of sternum VIII, as discussed under *Leucotrichia botoeaneanui*. The mesodorsal spines and short spines on the apical lobes of the phallus apex (Figs 30E, F) and the small single-pointed mesoventral process of sternum VII separate *Leucotrichia limpia* from the other 4 species in this group.

##### Description.

*Male*. Length of forewing 3.0–4.6 mm (n=13). Head unmodified, with 3 ocelli; antennae unmodified. Dorsum of head dark brown with light yellow setae; thorax dark brown with light yellow setae dorsally, brown ventrally; leg segments with brown setae. Forewings covered with fine dark brown setae with scattered patches of light yellow setae. *Genitalia*. Abdominal sternum VII with short, acute mesoventral process. Sternum VIII in ventral view with posterior margin concave. Segment IX anterolateral margin convex, posterolateral margin straight; in dorsal view anterior margin concave, posterior margin convex. Tergum × with dorsal sclerite simple; ventral sclerite semielliptic with tridentate posterior margin; membranous apex small, suborbicular. Subgenital plate with dorsal arm digitate, with slight mesal constriction; ventral arm broadest basally, tapering apically, in ventral view obovate. Inferior appendage basally subquadrate, apex truncate, bearing single dorsal spine; in ventral view entirely fused, subtriangular. Phallus with median complex bearing basal supports; apex bearing pair of stout spines dorsally, many short spines mesoventrally, and 2 membranous apical lobes bearing many short spines dorsally.

##### Material examined.

*Holotype male*: **USA: Texas:** Fort Davis, along Limpia Creek, 19.iv.1939, H.H. and J.A. Ross (INHS Trichoptera #22335) (INHS). *Allotype female*: Same data as for holotype (INHS). *Nontypes*: **COSTA RICA: Puntarenas:** roadside seep, route 2 just W km 234, 8.976°N, 83.299°W, el. 100m, 20.ii.1986, Holzenthal, Morse, and Fasth, 1 male, 1 female (UMSP); **MEXICO: Chiapas:** Chorreadero, Chiapa de Corzo, 11.viii.1967, O.S. Flint, Jr., 1 male, 2 females (NMNH); **Oaxaca:** Tamazulapan, 7-8.vi.1967, Flint and Ortiz, 1 male (NMNH); **San Luis Potosí:** Rancho Quemada, Rt. 85 km 353, 4-6.viii.1966, O.S. Flint, Jr., 1 male (NMNH); **USA: Arizona:** Yavapai Co., spring outfall at Bubbling Ponds Fish Hatchery, Page Springs, 23.iv.1993, S.R. Moulton and K.W. Stewart, 1 male, 1 female (in alcohol) (NMNH); Coconino Co., West Fork, 16 mi SW Flagstaff, elev. 6500’, 5.viii.1961, R.W. Hodges, 1 female (NMNH); Tucson, 2033 E. Helen St., 12.x.2010, R.B. Nagle, 1 male (NMNH); Tucson, 2033 E. Helen St., 12.x.2010, R.B. Nagle, 1 female (NMNH); **Texas:** Val Verde Co., Devils River, Dolan Creek., 29° 53.4'N, 100°59.6'W, 3.vi.1967, C.M. and O.S. Flint, Jr., 1 male (NMNH); Brewster Co., Big Bend National Park, Windows Creek, 5-10.iv.1993, J. Gelhaus, 1 female (NMNH); Val Verde Co., Devils River, Dolan Falls area, elev. 360 m, 17.v.1993, at light along Devils River, J. Gelhaus #589, Nelson, and Koenig, 2 males, 3 females (in alcohol) (NMNH); Val Verde, Co., Devils River, Dolan Falls, 29°53.0'N, 100°59.6'W, 2.vi.1997, C.M. and O.S. Flint, Jr., 1 male, 5 females (in alcohol) (NMNH); Brewster Co., Big Bend National Park, Chisos Mountains, spring fed creek in Oak Canyon (Window Trail), elev. 1425 m, 103°20'N, 29°17'W, malaise trap, 5-11.iv.1993, J. Gelhaus #559 and D. Koenig, 2 males, 2 females (in alcohol) (NMNH); Val Verde Co., Devils River, Dolan Creek, 29°53.4'N, 100°59.6'W, 3.vi.1997, C.M. and O.S. Flint, Jr., 2 females (NMNH); Brewster Co., Big Bend National Park, Windows Creek, 5-10.iv.1993, J. Gelhaus, 2 females (NMNH); Val Verde Co., San Felipe Springs, Del Rio, 29°22.1'N, 100°53.1'W, 1.vi.1997, C.M. and O.S. Flint, Jr., 9 females (NMNH).

##### Etymology.

Named for Limpia Creek, the location where the holotype was collected.

#### Leucotrichia mutica

Taxon classificationAnimaliaTrichopteraHydroptilidae

Flint, 1991

Fig. 31

mutica Flint, 1991: 39 [Type locality: Colombia, Dpto. Antioquia, Quebrada Honda, Marsella, 12km SW Fredonia; NMNH; male].

##### Diagnosis.

This species is similar to *Leucotrichia melleopicta*. These species share similarities in the phallus and the shape of the inferior appendage, as discussed under *Leucotrichia melleopicta*. *Leucotrichia mutica* can be distinguished from *Leucotrichia melleopicta* by the basal ridge on the mesoventral process of sternum VII, the ovate shape of the ventral arm of the subgenital plate in ventral view, and the dorsal sclerite on the phallus, which is much smaller than that of *Leucotrichia melleopicta*.

##### Description.

*Male*. Length of forewing 3.0 mm (n=1). Head unmodified, with 3 ocelli; antennae unmodified. Dorsum of head cleared, color brown; thorax brown with light yellow setae dorsally, brown ventrally; leg segments with golden brown setae. Forewings covered with fine yellow setae, apical 1/3 with dark brown setae. *Genitalia*. Abdominal sternum VII with acute mesoventral process with small basal ridge, apex rounded in ventral view. Sternum VIII in ventral view with posterior margin concave. Segment IX anterolateral margin convex, posterolateral margin straight; in dorsal view anterior margin sharply concave, posterior margin broadly concave. Tergum × with dorsal sclerite simple; ventral sclerite semielliptic with tridentate posterior margin; membranous apex truncate in dorsal view. Subgenital plate with dorsal arm not apparent; ventral arm broadest mesally, apex acute, laterally obscured from view by inferior appendage, in ventral view ovate. Inferior appendage dorsomesally “humped,” apex rounded, bearing single dorsal spine; in ventral view digitate. Phallus with basal half broken and missing; median complex not apparent; apex bearing single dorsal sclerite and pair of lateral sclerites; dorsal sclerite ovate, with apical emargination; lateral sclerites slender, sinuate.

##### Material examined.

*Holotype male*: **COLOMBIA: Antioquia:** Quebrada Honda, Marsella, 12 km SW Fredonia, elev. 1450 m, 22.ii.1983, O.S. Flint, Jr. (USNM 04525) (NMNH).

##### Etymology.

Unknown.

#### Leucotrichia padera

Taxon classificationAnimaliaTrichopteraHydroptilidae

Flint, 1991

Fig. 32

padera Flint, 1991: 41 [Type locality: Colombia, Dpto. Antioquia, Quebrada Espadera, 7 km E Medellín, road to Sta. Elena; NMNH; male].

##### Diagnosis.

**Figure 32.**
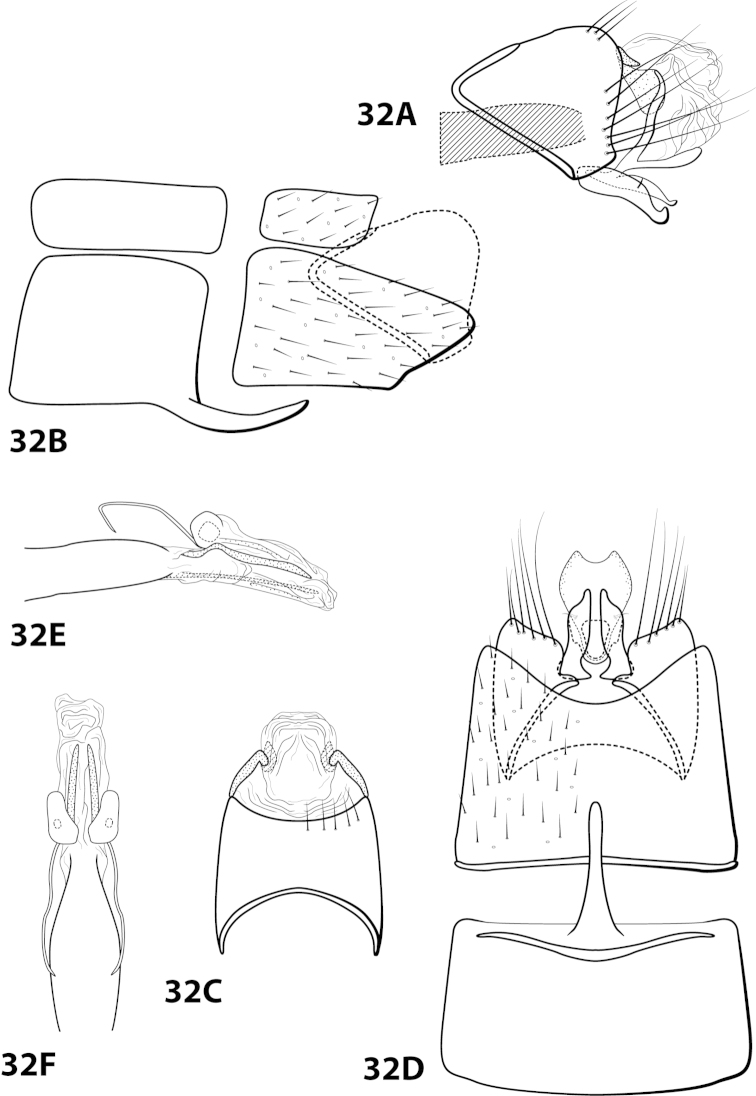
*Leucotrichia padera* Flint, 1991 (USNM104526). Male genitalia: **A** segments IX–X, lateral (base of phallus crosshatched) **B** segments VII–VIII and segment IX margin, lateral **C** segments IX–X, dorsal **D** segments VII–IX, ventral **E** phallus, lateral **F** phallus, dorsal.

This species is most similar to *Leucotrichia brochophora*, *Leucotrichia lerma*, and *Leucotrichia rhomba* sp. n. All four species share a similar form of the apex of the phallus, sternum VIII, and segment IX, as discussed under *Leucotrichia brochophora*. *Leucotrichia padera* differs from these species by the presence of the dorsal arm of the subgenital plate (Fig. 32A), which is absent in the other 3 species. It can also be recognized by the shape of the ventral arm of the subgenital plate in ventral view, which appears large and obovate with a rounded apical emargination (Fig. 32D).

##### Description.

*Male*. Length of forewing 3.0 mm (n=1). Head unmodified, with 3 ocelli; antennae unmodified. Dorsum of head cleared, brown; thorax brown with light yellow setae dorsally, light brown ventrally; leg segments with brown setae. Forewings covered with fine brown setae with 2 broad patches of light yellow setae, apex with dark brown setae. *Genitalia*. Abdominal sternum VII with elongate mesoventral process curving dorsad. Sternum VIII in ventral view with posterior margin concave. Segment IX anterolateral margin convex, posterolateral margin straight; in dorsal view anterior margin concave, posterior margin concave. Tergum × with dorsal sclerite small, reniform; ventral sclerite subdeltoid; membranous apex suborbicular. Subgenital plate elongate, mildly sinuate, extending dorsally along posterior edge of tergum × ventral sclerite; with dorsal arm digitate, apex enlarged; ventral arm with basal projection, broadest mesally, apex slightly hooked dorsad, in ventral view obovate with rounded apical emargination. Inferior appendage broadest basally, apex hooked dorsally, bearing single dorsal spine; in ventral view with basal projection on inner margin, apically digitate. Phallus apex bearing pair of elongate dorsolateral sclerites and internal ventral apodeme.

##### Material examined.

*Holotype male*: **COLOMBIA: Antioquia:** Quebrada Espadera, 7 km E Medellín, road to Santa Elena, 6.iii.1984, C.M. and O.S. Flint, Jr. (USNM 104526) (NMNH).

##### Etymology.

Presumably named for Quebrada Espadera, the location where the holotype was collected.

#### Leucotrichia pectinata

Taxon classificationAnimaliaTrichopteraHydroptilidae

Thomson & Holzenthal sp. n.

http://zoobank.org/F42E1742-A755-4F1D-BE7D-9718ECA0396C

Fig. 33

##### Diagnosis.

**Figure 33.**
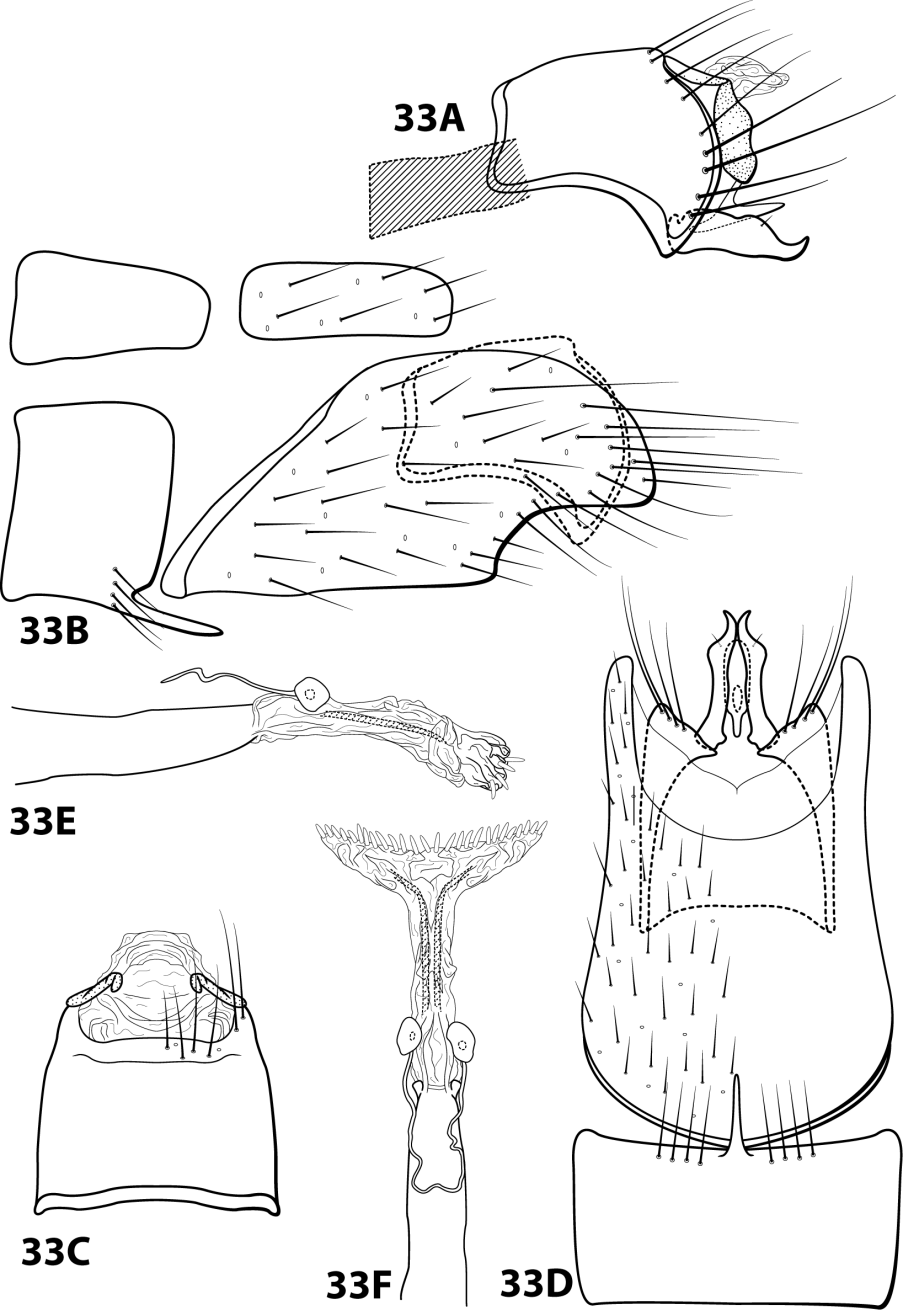
*Leucotrichia pectinata* sp. n. (UMSP000140619). Male genitalia: **A** segments IX–X, lateral (base of phallus crosshatched) **B** segments VII–VIII and segment IX margin, lateral **C** segments IX–X, dorsal **D** segments VII–IX, ventral **E** phallus, lateral **F** phallus, dorsal.

*Leucotrichia pectinata* sp. n., can be separated from all other species in the genus by several unique characteristics. The most striking characteristic is the apex of the phallus, which is broadened into a fan-shape and bears a row of peg-like setae on the posterior margin (Fig. 33F). In lateral view, sternum VIII bears a large posterodorsal projection (Fig. 33B) in contrast to the more common posteroventral projection. Sternum VII bears a row of prominent setae in addition to the mesoventral process (Fig. 33D).

##### Description.

*Male*. Length of forewing 4.5–4.7 (n=2). Head unmodified, with 3 ocelli; antennae unmodified. Dorsum of head dark brown with light yellow and dark brown setae; thorax dark brown with dark brown and light yellow setae dorsally, brown ventrally; leg segments with brown setae. Forewings covered with fine dark brown setae with 2 large patches of light yellow setae, 1st on apical 1/2 and 2nd on basal 1/2. *Genitalia*. Abdominal sternum VII with slender mesoventral process with row of prominent setae basally. Sternum VIII with posterodorsal projection, in ventral view with posterior margin concave. Segment IX anterolateral margin broadly produced dorsolaterally, posterolateral margin convex; in dorsal view anterior margin straight, posterior margin concave. Tergum × with dorsal sclerite simple; ventral sclerite semielliptic with crenulate posterior margin; membranous apex not well developed. Subgenital plate with dorsal arm not apparent; ventral arm with basal projection, tapering apically, in ventral view oblong with digitate basal projection. Inferior appendage with small basal emargination, apex hooked dorsally, bearing single dorsal spine; in ventral view outer margin crenulate. Phallus apex fan-shaped, with posterior margin bearing row of peg-like setae.

*Holotype male*: **ECUADOR:** Tungurahua, 13 km E Baños, 1550 m, 15.ix.1990, O.S. Flint, (UMSP000140619) (NMNH). *Paratype*: **ECUADOR:** Tungurahua, Baños (39 km E), 25.i.1976, 4200 ft, blacklight, Spangler et al., 1 male (NMNH).

##### Etymology.

*Pectinatus*, Latin for “comblike, toothed”, referring to the row of peg-like setae along the broad apical margin of the phallus.

#### Leucotrichia pictipes

Taxon classificationAnimaliaTrichopteraHydroptilidae

(Banks, 1911)

Figs 3C , 34

pictipes (Banks, 1911: 359) [Type locality: United States, New York, Johnstown, Hales Creek; MCZ; male; in *Orthotrichia*]. — Ross 1938: 10 [as *Stactobia pictipes* (Banks)]. — Ross 1944: 120 [to *Leucotrichia*]. — Flint 1970: 10 [male, distribution]. —Nielsen 1948: 11 [misidentified as *Ithytrichia confusa* Morton].

##### Diagnosis.

*Leucotrichia pictipes* is similar to *Leucotrichia fairchildi*, *Leucotrichia imitator*, and *Leucotrichia sarita*. *Leucotrichia pictipes* bears patches of scales, both on the dorsum of the head and the eversible membranous lobe beneath the posterolateral wart, which are absent in all 3 of the other species. The genitalia of *Leucotrichia pictipes* are similar to that *Leucotrichia imitator*, in having multiple membranous lobes arising from the apex of the phallus. While *Leucotrichia imitator* has 3 lobes, *Leucotrichia pictipes* can be distinguished by having 2 sets of symmetrically arranged lobes.

##### Description.

**Figure 34.**
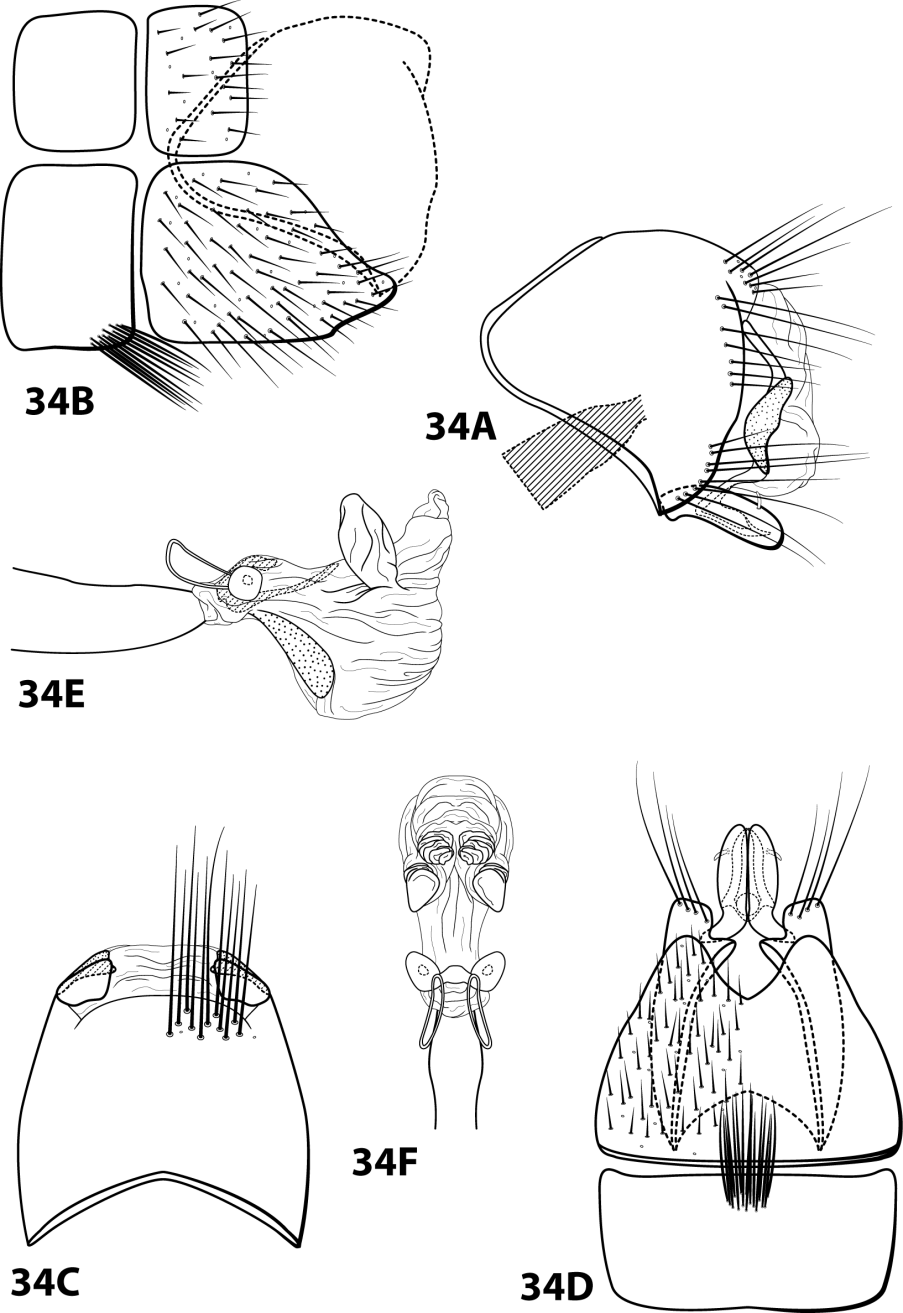
*Leucotrichia pictipes* (Banks, 1911) (MCZ11597). Male genitalia: **A** segments IX–X, lateral (base of phallus crosshatched): **B** segments VII–VIII and segment IX margin, lateral **C** segments IX–X, dorsal **D** segments VII–IX, ventral **E** phallus, lateral **F** phallus, dorsal.

*Male*. Length of forewing 2.5–4.0 (n=88). Head with patches of scales dorsally; posterolateral warts large, eversible, with scaled membranous lobes revealed when everted, with 2 ocelli; antennae with scape slightly enlarged, pedicel subtriangular, basal 3 flagellomeres narrow and compact. Dorsum of head brown with yellow setae; thorax brown with yellow setae dorsally, brown ventrally; leg segments with brown setae. Forewings covered with fine brown setae with transverse stripe of yellow setae on basal 1/2 and small scattered patches of yellow setae on distal 1/3. *Genitalia*. Abdominal sternum VII with mesoventral process replaced by tuft of dense, prominent setae. Sternum VIII in ventral view with posterior margin concave. Segment IX anterolateral margin convex, posterolateral margin convex; in dorsal view anterior margin concave, posterior margin broadly concave. Tergum × with dorsal sclerite slender; ventral sclerite semielliptic with tridentate posterior margin; membranous apex not well developed. Subgenital plate with dorsal arm not apparent; ventral arm hollow, apex acute (Fig. 34A), in ventral view base with crenulate margin, apex rounded. Inferior appendage digitate, bearing single dorsal spine; in ventral view apex rounded. Phallus apex bearing U-shaped internal apodeme, broad ventral sclerite, and 2 pairs of membranous apicodorsal lobes.

##### Material examined.

*Holotype male*: **USA: NEW YORK:** Johnstown, Hales Creek, collected in June, Alexander (MCZ11597) (MCZ). *Nontypes*: **MEXICO: Chihuahua:** Riito, Hwy 16, 10 mi E Yepachi, 28.vi.1987, Kondratieff and Baumann, 1 male, 1 female (in alcohol) (NMNH); **USA: California:** Tulare Co., 3 Rivers, 13.viii.1975, J.L. Cross, 19 males, 8 females (in alcohol) (NMNH); American River, Sacramento, 18.vi.1985, C.M. and O.S. Flint, Jr., 1 female (in alcohol) (NMNH); **Colorado:** Grand Co., Colorado River near Hot Sulphur Springs, 4.viii.1973, R.W. Baumann and W.P. Stark, 1 male, 2 females (in alcohol) (NMNH); Fremont Co., Cañon City, 27-29.v.1987, G.F. and J.F. Hevel, 2 males, 2 females (in alcohol) (NMNH); **Maryland:** Frederick Co., Small Creek, Hwy 77, 30.vi.1972, Baumann and Cross, 1 male (in alcohol) (NMNH); **Montana:** Madison Co., Madison River at Norris, 21.vi.1978, Groemhild, 4 males, 3 females (in alcohol) (NMNH); **New Mexico:** Sandoval Co., Valles Caldera National Preserve, Lower San Antonio Creek, NW border of Preserve, 35°57.8‘N106°36.9'W, 2.vii.2008, Parmenter et al., 17 males, 11 females (in alcohol) (NMNH); Sandoval Co., Caldera National Preserve, along E Fork Jemez River, “Dagobah,” 35°50.2'N, 106°30.1'W, 30.vi.2008, Parmenter et al., 1 male (in alcohol) (NMNH); **Oregon:** Douglas Co., S Umpqua River, 5 mi below junction of Myrtle Creek, Stn. M., 3.vi.1976, M.J. Stansburg, 2 males, 1 female (in alcohol) (NMNH); **Pennsylvania:** Warren Co., Spring Creek, 1.5 mi S Spring Creek village, 41°51.3'N, 79°32.4'W, 28.viii.1998, O.S. Flint, Jr., 1 male (in alcohol) (NMNH); Warren Co., Lainard Bush, Spring Creek, 41°52'N, 79°32'W, 4.vi.2000, L. Bush, 1 male (in alcohol) (NMNH); **Virginia:** Bath Co., Jackson River, Rt. 603, 2 mi S Rt. 687, 11.ix.1979, C.M. and O.S. Flint, Jr., 1 male, 1 female (in alcohol) (NMNH); Farquier Co., Broad Thoroughfare Gap, 27.v.1961, O.S. Flint, Jr., 2 males, 3 females (in alcohol) (NMNH); Bath Co., Jackson River at Rt. 603 bridge, 24.v.1974, B. Strickler, 1 male (in alcohol) (NMNH); Fauquier Co., Broad Run, Thoroughfare Gap, 15.vii1974, O.S. Flint, Jr., 1 male, 2 females (in alcohol) (NMNH); Fauquier Co., Broad Run, Thoroughfare Gap, 10.v.1974, O.S. Flint, Jr., 25 males, 20 females (in alcohol) (NMNH); **West Virginia:** Pendleton Co., Smoke Hole Camp, 28-29.viii.1963, R. and O. Flint, 2 males, 2 females (in alcohol) (NMNH); **Wisconsin:** St. Croix County, Apple River at Somerset on Highway 64, 22.v.1993, R.J. Blahnik, 1 male (UMSP); St. Croix County, Apple River at Somerset on Highway 64, 22.v.1993, R.J. Blahnik, 1 female (UMSP); Douglas Co., Upper St. Croix River near Gordon, 26.v.1994, R.J. Blahnik, 1 male (UMSP); Douglas Co., Upper St. Croix River near Gordon, 26.v.1994, R.J. Blahnik, 1 female (UMSP); **Wyoming:** Teton Co., Crawfish Creek, tributary to Lewis River, 12.v.1992, G. Roemhild, 2 males, 1 female (in alcohol) (NMNH); Yellowstone National Park, Madison Junction, 19.viii.1962, P. and P. Spangler, 1 male (in alcohol) (NMNH).

##### Etymology.

Unknown.

#### Leucotrichia procera

Taxon classificationAnimaliaTrichopteraHydroptilidae

Thomson & Holzenthal sp. n.

http://zoobank.org/7740ED61-07A6-4C92-B109-ACF0A8872D69

Figs 4C , 35

##### Diagnosis.

**Figure 35.**
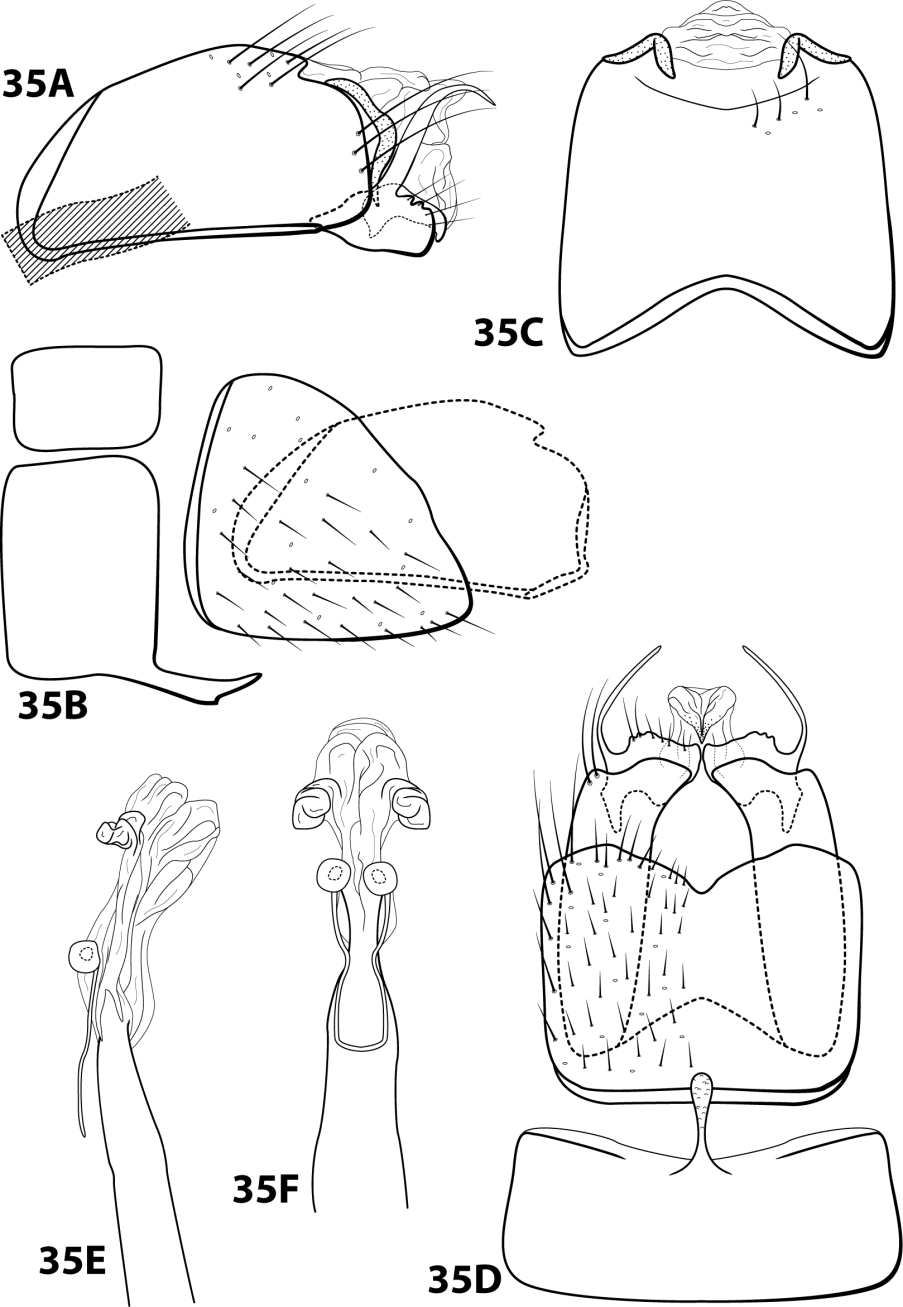
*Leucotrichia procera* sp. n. (UMSP000047406). Male genitalia: **A** segments IX–X, lateral (base of phallus crosshatched) **B** segments VII–VIII and segment IX margin, lateral **C** segments IX–X, dorsal **D** segments VII–IX, ventral **E** phallus, lateral **F** phallus, dorsal.

This species is known only from the male holotype. *Leucotrichia procera* can be separated from all other species in the genus by the unique projection on the dorsal margin of the inferior appendage, which extends to be almost parallel with the dorsal surface of tergum × (Fig. 35A). The phallus is also very simple, lacking any of the spines or sclerites commonly found in other species in the genus. Additionally, the scape of the antennae is enlarged and there is a patch of scales in place of setae on the dorsum of the head (Fig. 4C).

##### Description.

*Male*. Length of forewing 3.4 mm (n=1). Head with patches of scales dorsally, with 3 ocelli; antennae with enlarged scape, remaining flagellum missing. Dorsum of head brown with light yellow and dark brown setae; thorax dark brown with light yellow setae dorsally, dark brown ventrally; leg segments with dark brown setae. Forewings covered with fine dark brown setae with scattered patches of light yellow setae. *Genitalia*. Abdominal sternum VII with acute mesoventral process with small basal ridge, apex enlarged, rugose in ventral view. Sternum VIII in ventral view with posterior margin shallowly V-shaped. Segment IX anterolateral margin convex, posterolateral margin convex; in dorsal view anterior margin concave, posterior margin broadly concave. Tergum × with dorsal sclerite continuous with ventral sclerite; ventral sclerite sinuate; membranous apex not well developed. Subgenital plate with dorsal arm not apparent; ventral arm with both basal projection and apex pointing ventrad, in ventral view with apical spine very prominent. Inferior appendage with setose dorsal margin and prominent, elongate, curved dorsal projection; in ventral view with basal projection on outer margin, apex of dorsal projection curving inward. Phallus apex bearing pair of small subapicodorsal lobes, no sclerites or spines.

*Holotype male*: **BRAZIL: Minas Gerais:** Córrego da Serra de Ouro, Fino, Vale do Tropeiro, 20°12.371'S, 43°38.581'W, el. 1000 m, 8.x.2000, Paprocki, Salgado, and Isaac (UMSP000047406) (MZUSP).

##### Etymology.

*Procerus*, Latin for “tall, slender, long”, referring to the slender, elongate projection of the inferior appendage.

#### Leucotrichia repanda

Taxon classificationAnimaliaTrichopteraHydroptilidae

Thomson & Holzenthal sp. n.

http://zoobank.org/AC64E31D-22DD-46F4-9C6A-83BD69E1D4D8

Fig. 36

##### Diagnosis.

This species is most similar to *Leucotrichia ayura*. These species share similarities in the mesoventral process of sternum VII, the shape of sternum VIII, the shape of the inferior appendage, and sclerites observed on the phallus apex, as discussed under *Leucotrichia ayura*. *Leucotrichia repanda* sp. n., can be easily distinguished from *Leucotrichia ayura* by the presence of a pair of sharply bent dorsal sclerites, a pair of curved lateral sclerites, and an apical tuft of setae on the phallus apex, all of which are absent in *Leucotrichia ayura*. The presence of 3 apicodorsal setae on the inferior appendage also separates *Leucotrichia repanda* from *Leucotrichia ayura*, which lacks these setae.

##### Description.

*Male*. Length of forewing 1.9–2.4 mm (n=13). Head unmodified, with 3 ocelli; antennae unmodified. Dorsum of head dark brown with yellow and brown setae; thorax dark brown with yellow setae dorsally, brown ventrally; leg segments with brown setae. Forewings covered with fine dark brown setae with large patch of bright yellow setae on basal 1/3. *Genitalia*. Abdominal sternum VII with short mesoventral process. Sternum VIII in ventral view with posterior margin concave. Segment IX anterolateral margin produced mesally, posterolateral margin convex; in dorsal view anterior margin concave, posterior margin concave. Tergum × with dorsal sclerite elongate, with crenulate dorsal margin; ventral sclerite slender, simple; membranous apex not well developed. Subgenital plate with dorsal arm not apparent; ventral arm broadest basally, apex slightly hooked dorsad, in ventral view appearing “winged”. Inferior appendage apex rounded, bearing single mesodorsal seta and 3 apicodorsal setae; in ventral view entirely fused, apex emarginated. Phallus apex bearing apical setal tuft and 3 pairs of sclerites: 1st pair dorsal, sharply bent dorsad; 2nd pair dorsal, elongate; 3rd pair lateral, broad, curved anteriad.

*Holotype male*: **VENEZUELA: Sucre:** Península de Paria, Santa Isabel, Río Sta. Isabel, 10°44.294'N, 62°38.954'W, el. 20 m, 4.iv.1995, Holzenthal, Flint, and Cressa (UMSP000201685) (UMSP). *Paratypes*: same data as holotype, 3 males, 2 females (UMSP); same data as holotype, 1 male, 1 female (MIZA); **VENEZUELA: Aragua:** Est. Exp. Cataurito, ca. 32 km E Villa de Cura, 1100 m, 1.ii.1983, O.S. Flint, Jr., 2 males, 1 female (in alcohol) (NMNH); Est. Exp. Cataurito, 28.i.1983, O.S. Flint, Jr., 1 male (NMNH); Est. Exp. Cataurito, 1.ii.1983, O.S. Flint, Jr., 7 males (NMNH).

##### Etymology.

*Repandus*, Latin for “bent backward, turned up”, referring to the bent shape of the 1st pair of dorsal sclerites on the phallus.

#### Leucotrichia rhomba

Taxon classificationAnimaliaTrichopteraHydroptilidae

Thomson & Holzenthal sp. n.

http://zoobank.org/E685F3A9-DCC7-4F17-8D8F-9DEEBE111025

Fig. 37

##### Diagnosis.

**Figure 37.**
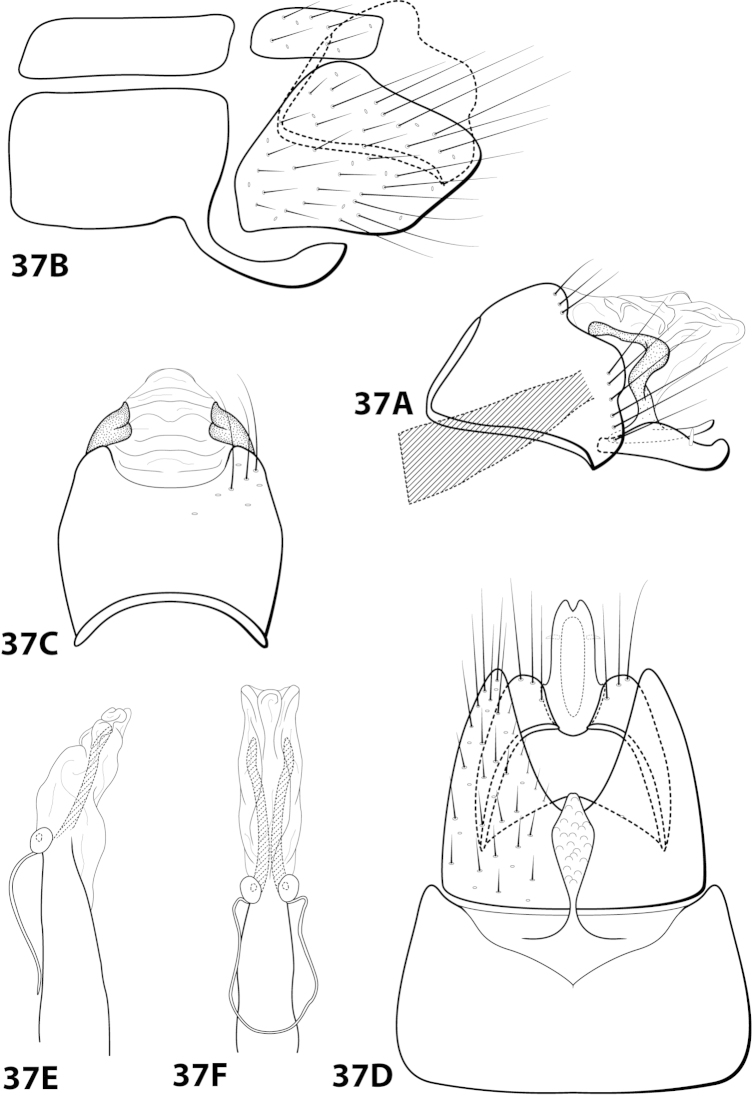
*Leucotrichia rhomba* sp. n. (UMSP000201350). Male genitalia: **A** segments IX–X, lateral (base of phallus crosshatched) **B** segments VII–VIII and segment IX margin, lateral **C** segments IX–X, dorsal **D** segments VII–IX, ventral **E** phallus, lateral **F** phallus, dorsal.

*Leucotrichia rhomba* sp. n., is known only from the male holotype. This species is most similar to *Leucotrichia brochophora*, *Leucotrichia lerma*, and *Leucotrichia padera*. These species share a similar form in the apex of the phallus, sternum VIII, and segment IX, as discussed under *Leucotrichia brochophora*. *Leucotrichia rhomba* can be separated from the other 3 species by the size and shape of the mesoventral process of sternum VII (Fig. 37B, D). The apex of the process is enlarged and, in ventral view, rhomboid and rugose. The posterior margin of sternum VIII is also much more deeply concave in *Leucotrichia rhomba* than in any of the other 3 species.

##### Description.

*Male*. Length of forewing 2.4 mm (n=1). Head unmodified, with 3 ocelli; antennae unmodified. Dorsum of head brown with yellow and dark brown setae; thorax brown with light brown setae dorsally, brown ventrally; leg segments with brown setae. Forewings covered with fine brown setae with light yellow patch extending from basal 3/4 along the length of outer edge. *Genitalia*. Abdominal sternum VII with elongate mesoventral process with apex enlarged; in ventral view apex rhomboid, rugose. Sternum VIII in ventral view with posterior margin deeply concave. Segment IX anterolateral margin produced mesally, posterolateral margin irregular; in dorsal view anterior margin concave, posterior margin concave. Tergum × with dorsal sclerite continuous with ventral sclerite; ventral sclerite with rounded projection on posterior margin; membranous apex subtriangular. Subgenital plate fused with ventral sclerite of tergum X; with dorsal arm not apparent; ventral arm digitate with basal projection, in ventral view oblong. Inferior appendage broadest mesally, apex hooked dorsally, bearing single dorsal spine; in ventral view entirely fused, oval, basally enlarged, apex with shallow emargination. Phallus apex not well expanded, bearing pair of internal apodemes.

*Holotype male*: **COSTA RICA: Puntarenas:** Río Jaba at rock quarry, 1.4 km (air) W Las Cruces, 8.79°N, 82.97°W, 9.viii.1990, el. 1150 m, Holzenthal, Blahnik, and Muñoz, 1 male (UMSP000201350) (UMSP).

##### Etymology.

*Rhombus*, Latin for “rhomboid”, a quadrilateral of which only the opposite sides and angles are equal, referring to the shape of the enlarged apex of the mesoventral process of sternum VII.

#### Leucotrichia riostoumae

Taxon classificationAnimaliaTrichopteraHydroptilidae

Thomson & Holzenthal sp. n.

http://zoobank.org/60CCD9CC-E241-45E9-8880-3162973C0527

Fig. 38

##### Diagnosis.

This species is similar to *Leucotrichia forrota* and *Leucotrichia inops*. These species share a similar combination of characteristics present in the phallus, as discussed under *Leucotrichia forrota*. Of these species, *Leucotrichia riostoumae* sp. n., is most similar to *Leucotrichia inops*, as discussed under *Leucotrichia inops*. *Leucotrichia riostoumae* can be distinguished by the rugose ventral surface of the mesoventral process of sternum VII, which is lacking in *Leucotrichia inops*. The membranous ridges along the apex of the phallus, which give it a star-like appearance when viewed dorsally, are also a feature unique to *Leucotrichia riostoumae*.

##### Description.

*Male*. Length of forewing 3.6–4.2 mm (n=11). Head unmodified, with 3 ocelli; antennae unmodified. Dorsum of head dark brown with yellow and dark brown setae; thorax dark brown with bright yellow setae dorsally, dark brown ventrally; leg segments with dark brown setae. Forewings covered with fine dark brown setae with large patch of yellow setae on basal 2/3. *Genitalia*. Abdominal sternum VII with digitate, rugose mesoventral process with small basal ridge. Sternum VIII in ventral view with posterior margin concave. Segment IX anterolateral margin produced dorsolaterally, posterolateral margin convex; in dorsal view anterior margin concave, posterior margin broadly convex. Tergum × with dorsal sclerite simple; ventral sclerite with rounded projection on posterior margin; membranous apex small. Subgenital plate with dorsal arm digitate, broadest basally; ventral arm slender, digitate, in ventral view triangular. Inferior appendage basally subquadrate, apex rounded, without dorsal spine; in ventral view apically digitate. Phallus with basal loop not apparent; apex curving ventrad and laterad, with series of membranous spines appearing stellate in dorsal view.

*Holotype male*: **ECUADOR: Imbabura:** Reserva los Cedros, Río de la Plata, 00.32495°N, 78.78084°W, el. 1587 m, 18.x.2011, Holzenthal, Ríos, Encalada, and Acosta, (UMSP000140832) (UMSP). *Paratypes*: same data as holotype, 5 males (UMSP); same data as holotype, 3 males (MZUTI); same data as holotype, 2 males (NMNH).

##### Etymology.

Named in honor of Dr. Blanca Ríos-Touma, an aquatic ecologist and colleague who helped collect the holotype specimen.

#### Leucotrichia sarita

Taxon classificationAnimaliaTrichopteraHydroptilidae

Ross, 1944

Figs 4D , 39

sarita Ross, 1944: 274 [Type locality: United States, Texas, Balmorhea, along stone irrigation flume; INHS; male]. — Flint 1970: 9 [male, larva, case, distribution].

##### Diagnosis.

*Leucotrichia sarita* is similar to *Leucotrichia fairchildi*, *Leucotrichia imitator*, and *Leucotrichia pictipes*. *Leucotrichia sarita* is distinct from *Leucotrichia imitator* and possesses a more modified head, due to the presence of eversible posterolateral warts (Fig. 4D). It does not have any of the further head modifications found on either *Leucotrichia fairchildi* or *Leucotrichia pictipes*. The presence of the membranous lobes on the phallus terminating in sclerotized spines also distinguishes *Leucotrichia sarita* from the other 3 species of the *pictipes* species group.

##### Description.

**Figure 39.**
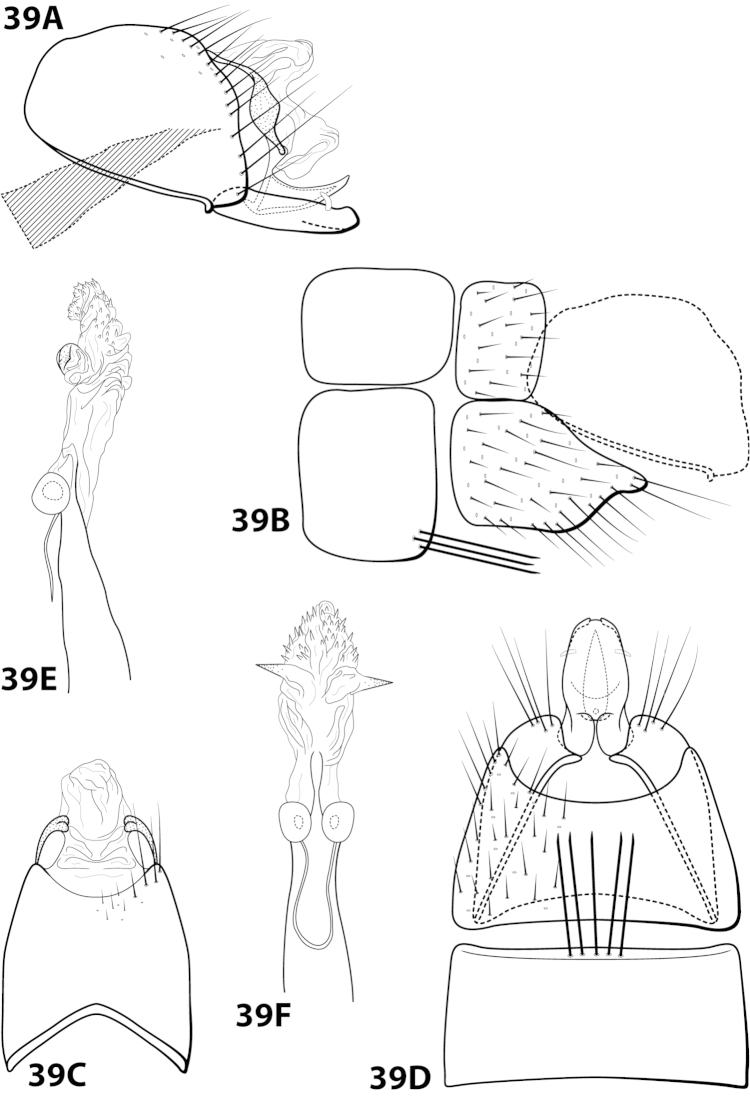
*Leucotrichia sarita* Ross, 1944 (INHS22339). Male genitalia: **A** segments IX–X, lateral (base of phallus crosshatched) **B** segments VII–VIII and segment IX margin, lateral **C** segments IX–X, dorsal **D** segments VII–IX, ventral **E** phallus, lateral **F** phallus, dorsal.

*Male*. Length of forewing 2.1–3.0 mm (n=306). Head with posterolateral wart large, eversible, with membranous lobe beneath, with 2 ocelli; antennae unmodified. Dorsum of head brown with yellow setae; thorax brown with dark brown and yellow setae dorsally, brown ventrally; leg segments with brown setae. Forewings covered with fine dark brown setae with stripe of yellow setae running the length of basal 1/3 and scattered patches of yellow setae near apex. *Genitalia*. Abdominal sternum VII with mesoventral process absent, in its place a few dark, prominent setae. Sternum VIII in ventral view with posterior margin concave. Segment IX anterolateral margin convex, posterolateral margin irregular; in dorsal view anterior margin concave, posterior margin concave. Tergum × with dorsal sclerite slender; ventral sclerite semielliptic with crenulate posterior margin; membranous apex with dorsal and ventral lobes. Subgenital plate with dorsal arm not apparent; ventral arm hollow, subtriangular, with basal projection, curved dorsad, in ventral view lanceolate. Inferior appendage straight, digitate, bearing single dorsal spine; in ventral view broadly fused, apex rounded, with small apical “lip” (Fig. 39D). Phallus apex bearing pair of mesolateral lobes ending in sclerotized spine and numerous small apical spines on dorsal and lateral surface.

##### Material examined.

*Holotype male*: **USA: Texas:** Balmorhea, along stone irrigation flume, 19.iv.1939, H.H. and J.A. Ross (in alcohol) (INHS Trichoptera #22339) (INHS). *Nontypes*: **COSTA RICA: Alajuela:** Río Agrio, ca. 3.5 km NE Bajos del Toro, 10.243°N, 84.279°W, 20.viii.1990, el. 1290m, Holzenthal et al., 5 males, 9 females (UMSP); **Cartago:** Reserva Tapanti, Quebrada Palmitos and falls, 9.72°N, 83.78°W, 23.viii.1990, el. 1400 m, Holzenthal and Huisman, 1 male (UMSP); **Guanacaste:** Parque Nacional Guanacaste, El Hacha, Queb. Alcornoque, 11.009N 85.577W, 26. vii.1987, el. 250m, Holzenthal, Morse, and Clausen, 1 male (in alcohol) (UMSP); **Las Canas:** 13.vii.1965, P.J. Spangler, 1 female (NMNH); Río Seco, NW of Esparta, 23.vii.1967, O.S. Flint, Jr., 1 male (in alcohol) (NMNH); 13.vii.1965, P.J. Spangler, 4 males, 20 females (in alcohol) (NMNH); **Puntarenas:** Río Jaba at rock quarry, 1.4km (air) W Las Cruces, 8.79°N, 82.97°W, 9.viii.1990, el. 1150m, Holzenthal, Blahnik, and Muñoz, 1 male (UMSP); **GUATEMALA: Retalhuleu:** Puente El Niño, 16.vii.1966, Flint and Ortiz, 1 female (NMNH); **San Marcos:** Puente Ixben, 15.vii.1966, Flint and Ortiz, 3 males (NMNH); **Suchitepéquez:** Cuyotenango, 10-20.vi.1966, Flint and Ortiz, 1 female (NMNH); **MEXICO: Chiapas:** Rt. 35, 4 km N Arriaga, 9.xii.1975, C.M. and O.S. Flint, Jr., 1 male (in alcohol) (NMNH); near Pijijiapan, 5.vii.1965, P.J. Spangler, 1 male (in alcohol) (NMNH); **Guerrero:** Acahuizoltla, 10.xu.1982, J. Bueno and E. Borrera, 1 male (in alcohol) (NMNH); **Michoacán:** San Lorenzo, Rt. 15 km 206, 14-15.vii.1966, Flint and Ortiz, 4 males, 2 females (NMNH); same, 3 males, 2 females (in alcohol) (NMNH); **Morelos:** Xochitepec, 12-14.vii.1965, Flint and Ortiz, 8 males, 3 females (NMNH); near Xochitepec, Rt. 95 km 91, 1.viii.1965, O.S. Flint, Jr., 7 males, 9 females (in alcohol) (NMNH); **Oaxaca:** Isthmus Tehuantepec, Jaltepec, 21.v.1964, F.S. Blanton, 1 male (in alcohol) (NMNH); Tehuantepec, 23.vii.1964, P.J. Spangler, 6 females (in alcohol) (NMNH); **Veracruz:** near El Lencero, Rt. 140 km 437, 22.vii.1965, Flint and Ortiz, 5 males (NMNH); Plan del Río Ver, Rt. 140 km 368, 23.vii.1965, Flint and Ortiz, 2 males, 7 females (NMNH); Fortín de las Flores, ?.vi.1964, light trap, F.S. Blantoni, 1 male (in alcohol) (NMNH); Río Tacolapan, Rt. 180 km 551, 25-26.vii.1966, Flint and Ortiz, 1 male (in alcohol) (NMNH); Cultlahuac, 10-12.viii.1964, P.J. Spangler, 2 males, 5 females (in alcohol) (NMNH); **USA: California:** Truckee, 8.viii.1915, H.G. Dyar, 1 female (NMNH); **Maryland:** Washington Co., C and O Canal, Harpers Ferry Vicinity, 7.v.1972, G.F. and S. Hevel, 1 female (NMNH); Montgomery Co., Potomac River, Cardero Creek recreation area, 5.ix.1976, J. Heppner, 1 male (NMNH); **Nevada:** Reno, 2.viii.1916, H.G. Dyar, 2 males, 5 females (NMNH); Reno, 3.viii.1916, H.G. Dyar, 54 males, 3 females (NMNH); **New Mexico:** Jemez Springs, 4.vii.1953, W.W. Wirth, 1 male (NMNH); **Oregon:** Klamath Lake, 27.vii.????, Dyar and Caudell, 3 males, 2 females (NMNH); **Texas:** Val Verde Co., Devils River, Dolan Falls area, elev. 360 m, 17-20.v.1993, malaise trap with UV light at spring along Dolan Creek, Gelhaus #589, Nelson, and Koenig, 8 males, 7 females (in alcohol) (NMNH); Val Verde Co., San Felipe Springs, Del Rio, 29°22.1'N, 100°53.1'W, 1.vi.1997, C.M. and O.S. Flint, Jr., 169 males, 88 females (NMNH); **Virginia:** Highland Co., Locust Springs, beaver ponds, 12.ix.1979, C.M. and O.S. Flint, Jr., 1 female (NMNH); Fauquier Co., Broad Run, Thoroughfare Gap, 10.v.1974, O.S. Flint, Jr., 3 males, 6 females (NMNH); Madison, Criglersville, 1.6 km NW, 38°28.4'N, 78°19.9'W, elev. 185 m, 19.v.2005, W.N. and D. Mathis, 1 male (NMNH).

##### Etymology.

Unknown.

#### Leucotrichia sidneyi

Taxon classificationAnimaliaTrichopteraHydroptilidae

Thomson & Holzenthal sp. n.

http://zoobank.org/D93C915E-5A67-41B6-9154-31101EE2FE35

Fig. 40

##### Diagnosis.

*Leucotrichia sidneyi* sp. n., is most similar to *Leucotrichia brasiliana*. These species share similarities in a suite of characters observed on the phallus, as discussed under *Leucotrichia brasiliana*. *Leucotrichia sidneyi* can be separated by the absence of a dorsal spine on the inferior appendage, pairs of separate dorsal and ventral sclerites on the phallus apex, and a much shallower concave posterior margin on sternum VIII than *Leucotrichia brasiliana*. Additionally, the inferior appendages are fused in *Leucotrichia sidneyi* and separate in *Leucotrichia brasiliana*.

##### Description.

*Male*. Length of forewing 2.9–3.0 mm (n=6). Head unmodified, with 3 ocelli; antennae unmodified. Dorsum of head brown with yellow setae; thorax dark brown with golden yellow setae dorsally, brown ventrally; leg segments with dark brown setae. Forewings covered with fine mottled green-yellow setae with dark brown setae on edges. *Genitalia*. Abdominal sternum VII with elongate mesoventral process with apex enlarged, rugose in ventral view. Sternum VIII in ventral view with posterior margin concave or concave with pointed mesal emargination. Segment IX anterolateral margin convex, posterolateral margin convex; in dorsal view anterior margin convex, posterior margin concave. Tergum × with dorsal sclerite slender; ventral sclerite with rounded projection on posterior margin; membranous apex suborbicular. Subgenital plate with dorsal arm not apparent; ventral arm rounded basally, apex truncate, in ventral view oblong with base slightly enlarged and apex broadly rounded. Inferior appendage broadest mesally, apex rounded, without dorsal spine; in ventral view entirely fused, basally enlarged, apex truncate. Phallus apex bearing pair of dorsal sclerites, ventral sclerite, and membranous ventral “hump”.

*Holotype male*: **VENEZUELA: T. F. A.:** Camp IV, 0°58'N, 65°57'W, Cerro d. l. Neblina, 760m, 15-18.iii.1984, O.S. Flint, Jr. (UMSP000140465) (NMNH). *Paratypes*: same data as holotype, 3 males, 11 females (NMNH); same data as holotype, 2 males, 2 females (UMSP).

##### Etymology.

Named in honor of R. E. Thomson’s father, Sid Thomson, a fly fisherman and the only other member of the family who can recognize a caddisfly.

#### Leucotrichia tapantia

Taxon classificationAnimaliaTrichopteraHydroptilidae

Thomson & Holzenthal sp. n.

http://zoobank.org/1F32FD62-1214-46DE-B2BB-3B0C6CEFE3EA

Fig. 41

##### Diagnosis.

This species is similar to *Leucotrichia bicornuta*, *Leucotrichia dianeae*, and *Leucotrichia extraordinaria*. These species share a similar combination of characteristics present in the phallus and the posterolateral margin of sternum VIII, as discussed under *Leucotrichia bicornuta*. Of these species, *Leucotrichia tapantia* sp. n., is most similar to *Leucotrichia dianeae*. Each has a large tuft of prominent setae at the apex of the posterolateral projection of sternum VIII; a small, mesal projection on the posterior margin of sternum VIII, and larger, more prominent apical lobes on the phallus apex than either *Leucotrichia bicornuta* or *Leucotrichia extraordinaria*. Characteristics that can be used to distinguish *Leucotrichia tapantia* from *Leucotrichia dianeae* include a much longer basal loop of the phallus midlength complex and the absence of a dorsal arm on the subgenital plate. The prominent apical spine of the ventral arm on the subgenital plate, when viewed ventrally, and the presence of 2 small dorsal setae, in addition to the single dorsal spine on the inferior appendage separate *Leucotrichia tapantia* from all 3 of the other species.

##### Description.

**Figure 41.**
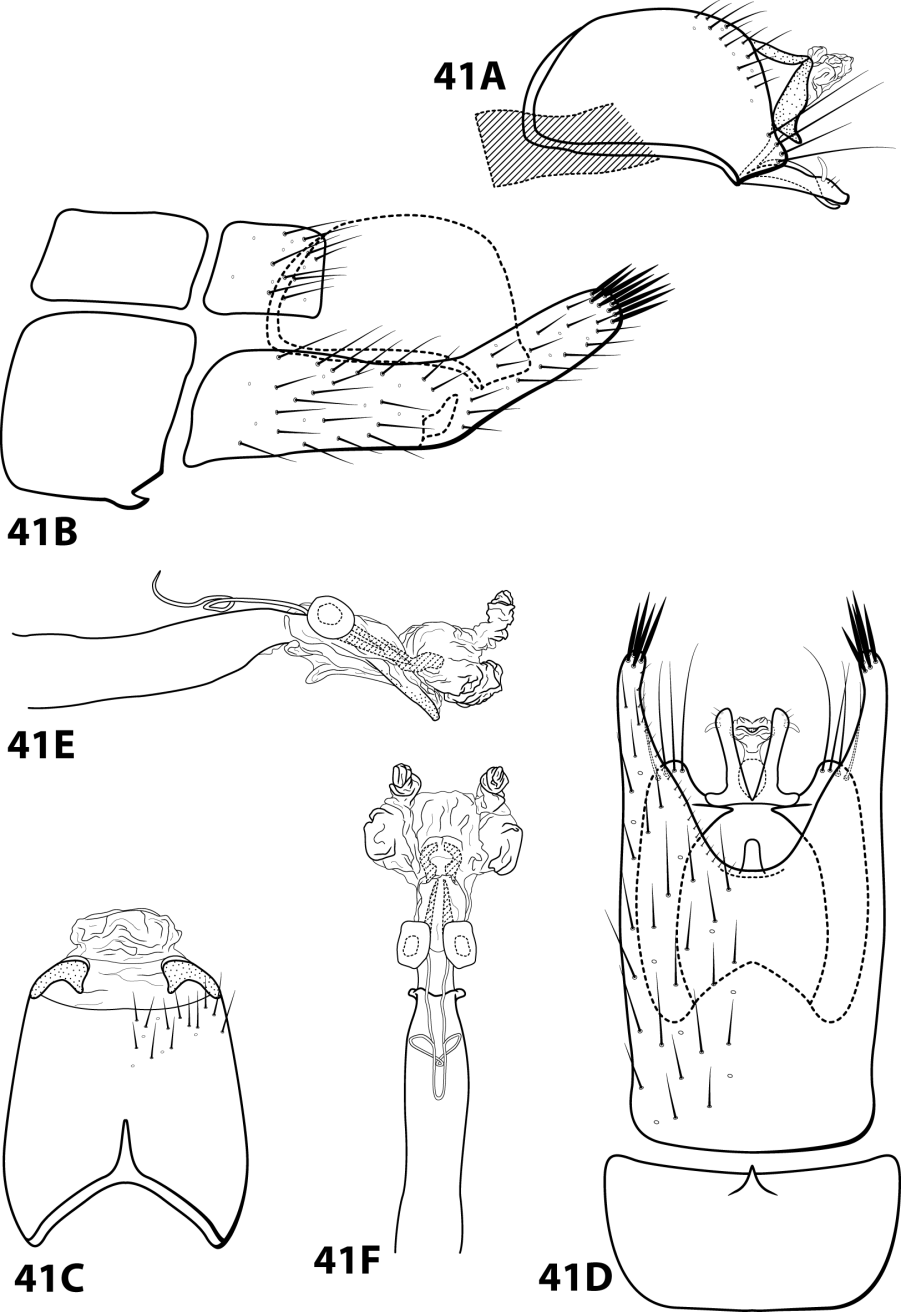
*Leucotrichia tapantia* sp. n. (UMSP000201359). Male genitalia: **A** segments IX–X, lateral (base of phallus crosshatched) **B** segments VII–VIII and segment IX margin, lateral **C** segments IX–X, dorsal **D** segments VII–IX, ventral **E** phallus, lateral **F** phallus, dorsal.

*Male*. Length of forewing 3.4–3.9 mm (n=19). Head unmodified, with 3 ocelli; antennae unmodified. Dorsum of head dark brown with yellow setae; thorax dark brown with yellow setae dorsally, brown ventrally; leg segments with brown setae. Forewings covered with fine dark brown setae with broad streak of yellow setae down outer edge. *Genitalia*. Abdominal sternum VII with short, pointed mesoventral process. Sternum VIII lateral projection elongate, extending dorsad, apex bearing tuft of prominent setae (Fig. 41B), in ventral view with posterior margin concave with digitate mesal projection (Fig. 41D). Segment IX anterolateral margin convex, posterolateral margin convex; in dorsal view anterior margin concave, posterior margin straight. Tergum × with dorsal sclerite slender; ventral sclerite semielliptic with crenulate posterior margin; membranous apex small. Subgenital plate with dorsal arm not apparent; ventral arm slender, sinuate, broadest mesally, in ventral view with apical spine very prominent. Inferior appendage straight, bearing 1 large dorsal spine and 2 small setae; in ventral view fused basally, digitate. Phallus apex bearing internal apodemes and pair of prominent apicodorsal lobes.

*Holotype male*: **COSTA RICA: Cartago:** Reserva Tapantí, waterfall, ca. 1km (road) NW tunnel, 9.69°N, 83.76°W, 2-3.viii.1990, el. 1600m, Holzenthal, Blahnik, Muñoz (UMSP000201359) (UMSP). *Paratypes*: same data as holotype, 46 males (UMSP); same data as holotype, except 10.vi.1988, C.M. and O.S. Flint, R.W. Holzenthal, 13 males (NMNH).

##### Etymology.

Named for Reserva Tapantí, the location where the holotype was collected. The name was suggested by Dr. Steve Harris, an aquatic biologist and Trichoptera taxonomist at Clarion University, who first recognized the species as new.

#### Leucotrichia termitiformis

Taxon classificationAnimaliaTrichopteraHydroptilidae

Botosaneanu, 1993

Fig. 42

termitiformis Botosaneanu, in Botosaneanu and Alkins-Koo 1993: 13 [Type locality: Trinidad, stream below Maracas waterfall; ZMUA; male; larva]. — Botosaneanu and Sakal 1992: 201 [biology].

##### Diagnosis.

*Leucotrichia termitiformis* can be recognized by the shape of the inferior appendage, which is somewhat similar to *Leucotrichia tubifex*. In lateral view, the inferior appendage is large and truncate; in ventral view, it is large, fused, and slightly spatulate. The inferior appendage of *Leucotrichia tubifex* differs in having a small mesodorsal spine and in being separate and smaller in size. Additionally, sternum VIII lacks peg-like setae in *Leucotrichia termitiformis* and is more laterally elongate than that of *Leucotrichia tubifex*.

##### Description.

Phallus of holotype missing, redescribed from Botosaneanu and Alkins-Koo (1993). *Male*. Length of forewing 3.9 mm (n=1). Head unmodified, with 3 ocelli; antennae unmodified. Color in alcohol brown. *Genitalia*. Abdominal sternum VII with digitate mesoventral process. Sternum VIII in ventral view with posterior margin concave. Segment IX anterolateral margin convex, posterolateral margin convex; in dorsal view anterior margin shallowly concave, posterior margin concave. Tergum × with dorsal sclerite continuous with ventral sclerite; ventral sclerite sinuate; membranous apex not well developed. Subgenital plate fused with ventral sclerite of tergum X; with dorsal arm slender, tapering apically; ventral arm slender, with basal projection, apex slightly hooked dorsad, in ventral view oblong. Inferior appendage large, apex truncate, bearing single dorsal spine; in ventral view entirely fused, with basal projection on outer margin, apex emarginated. Phallus with median complex bearing 2 separate basal filaments; apex bearing dark, U-shaped, dorsomesal sclerite and pair of apicolateral sclerites.

##### Material examined.

*Holotype male*: **TRINIDAD:** stream below Maracas waterfall (in alcohol) (UMSP000140326) (ZMUA).

##### Etymology.

Specific epithet refers to the typical “termitiform” shape of the larval abdominal segments.

#### Leucotrichia tritoven

Taxon classificationAnimaliaTrichopteraHydroptilidae

Flint, 1996

Fig. 43

tritoven Flint, 1996: 89 [Type locality: Trinidad, streamlet, Lalaja Road, 10°43'N, 61°17'W; NMNH; male].

##### Diagnosis.

**Figure 43.**
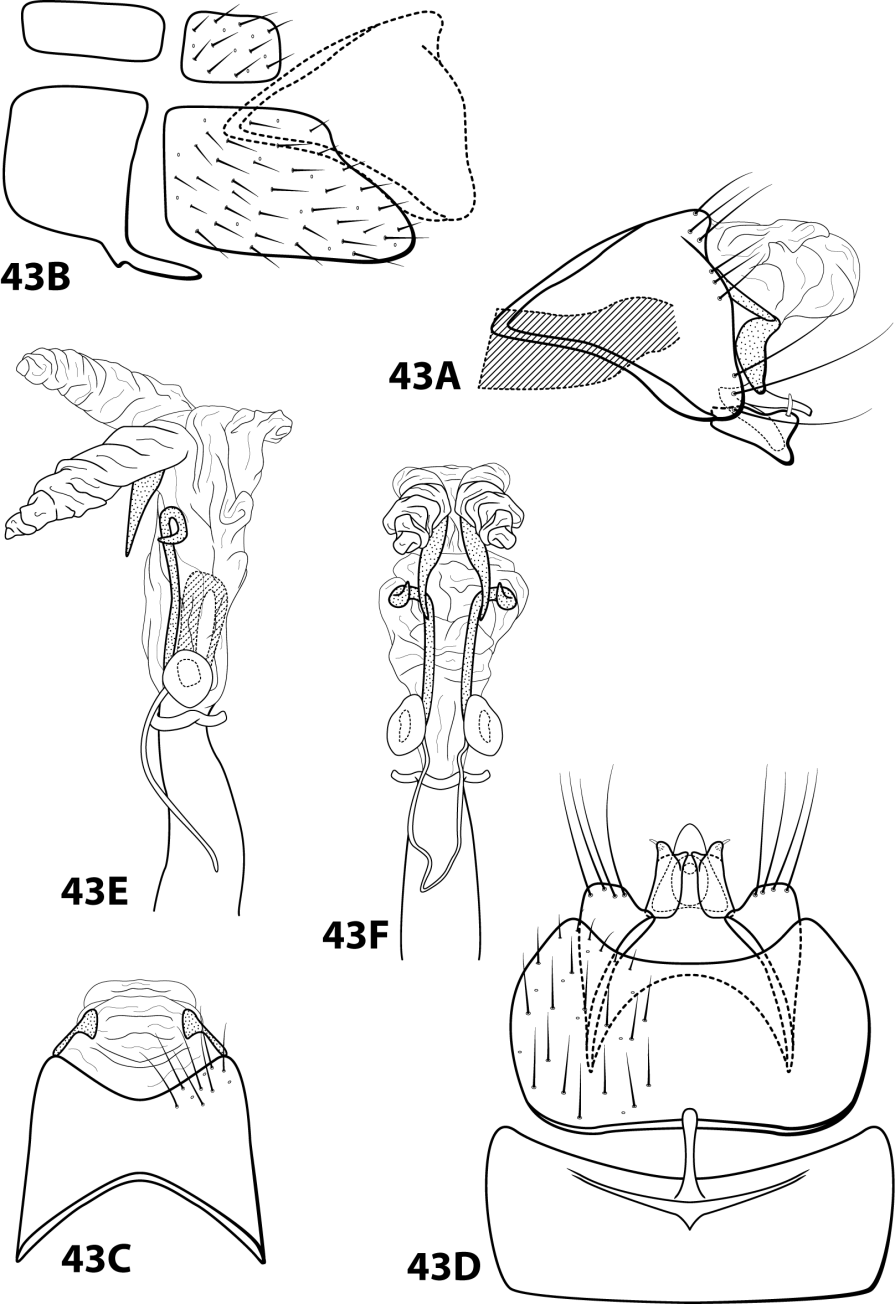
*Leucotrichia tritoven* Flint, 1996 (USNM105437). Male genitalia: **A** segments IX–X, lateral (base of phallus crosshatched) **B** segments VII–VIII and segment IX margin, lateral **C** segments IX–X, dorsal **D** segments VII–IX, ventral **E** phallus, lateral **F** phallus, dorsal.

*Leucotrichia tritoven* is most similar to *Leucotrichia kateae*. In both of these species, the dorsal spine of the inferior appendage is located on a dorsomesal “hump” (Fig. 43A). There is also a pair of membranous lobes arising from the apex of the phallus in each of these species, from the base of which a pair of large, pointed sclerites also arises. A pair of elongate, apically curling sclerites on the phallus distinguishes *Leucotrichia tritoven* from *Leucotrichia kateae*, which lacks these elongate sclerites. Additionally, the dorsal arm of the subgenital plate in *Leucotrichia tritoven* is digitate, whereas in *Leucotrichia kateae* it is apically acute.

##### Description.

*Male*. Length of forewing 1.5–2.0 mm (n=24). Head unmodified, with 3 ocelli; antennae unmodified. Dorsum of head brown with light yellow setae; thorax brown with light yellow setae dorsally, brown ventrally; leg segments with brown setae. Forewings covered with fine yellow setae with dark brown setae at edges and apex. *Genitalia*. Abdominal sternum VII with digitate mesoventral process with small basal ridge. Sternum VIII in ventral view with posterior margin concave. Segment IX anterolateral margin produced mesally, posterolateral margin irregular; in dorsal view anterior margin concave, posterior margin concave. Tergum × with dorsal sclerite slender, elongate; ventral sclerite semielliptic; membranous apex suborbicular. Subgenital plate with dorsal arm digitate, apex truncate; ventral arm digitate, laterally obscured from view by inferior appendage, in ventral view triangular. Inferior appendage dorsomesally “humped,” apex rounded, bearing single dorsal spine; in ventral view with mitten-like “thumb” on inner margin (Fig. 43D). Phallus apex bearing U-shaped internal apodeme, pair of apical membranous lobes, and 2 pairs of elongate sclerites: 1st pair lateral, elongate, apically curled, 2nd pair acute, apicodorsal sclerites extending anteriad.

##### Material examined.

*Holotype male*: **TRINIDAD:** Lalaja Road, streamlet, 10°43'N, 61°17'W, elev. 520 m, 26.vi.1993, by net, O.S. Flint, Jr. and W.N. Mathis (USNM 105437) (NMNH). *Paratypes*: Same data as holotype, 3 males, 1 female (NMNH); **TRINIDAD:** Lalaja Road, Guanapo River, elev. 480 m, 10°43'N, 61°17'W, 26.vi.1993, by net, O.S. Flint, Jr. and W.N. Mathis, 2 males, 1 female (in alcohol) (NMNH); **TOBAGO:** Charlotteville, 4 km S, big waterfall, 11°19'N, 60°33'W, elev 125 m, 10.vi.1993, by net, O.S. Flint, Jr. and W.N. Mathis, 1 male (in alcohol) (NMNH); Roxborough, 6.5 km N, B1/5, elev. 390 m, 11°17'N, 60°35'W, 14.vi.1993, by net, O.S. Flint, Jr. and W.N. Mathis, 6 males (NMNH); **VENEZUELA:** Sucre Parque Nacional, Península de Paria, Uquire, Río La Viuda, 10°42.830'N, 61°57.661'W, elev. 15 m, 30.iii-1.iv.1995, Holzenthal, Flint, and Cressa, 10 males, 3 females (NMNH). *Nontypes*: **GUYANA:** Dubulay Ranch, Warniabo Creek, 5°39.8'N, 57°53.4'W, 14-19.iv.1995, O.S. Flint, Jr., malaise trap night collection, 1 male (in alcohol) (NMNH).

##### Etymology.

Unknown.

#### Leucotrichia tubifex

Taxon classificationAnimaliaTrichopteraHydroptilidae

Flint, 1964

Fig. 44

tubifex Flint, 1964: 44 [Type locality: Puerto Rico, Maricao, at fish hatchery; NMNH; male; female, larva, pupa, case]; 1970: 7 [male, larva, case, distribution]. — Botosaneanu 1991: 116 [distribution]. — Botosaneanu and Bolland 1997: 71 [parasitized by mite, genus *Leptus*].

##### Diagnosis.

*Leucotrichia tubifex* can be recognized by the bulbous apex of the phallus. This species is similar to *Leucotrichia gomezi* in the subquadrate appearance of sternum VIII when viewed laterally and differs in bearing small peg-like setae on a small posteroventral projection not present in *Leucotrichia gomezi*. The posterior margin of sternum VIII is convex and U-shaped in *Leucotrichia tubifex*, while it is straight and without emargination in *Leucotrichia tubifex*. Additional similarities and differences are discussed under *Leucotrichia gomezi*.

##### Description.

*Male*. Length of forewing 3.6–5.2 (n=24). Head unmodified, with 3 ocelli; antennae unmodified. Dorsum of head brown with yellow setae; thorax brown with yellow setae dorsally, light brown ventrally; leg segments with light brown setae. Forewings covered with fine golden yellow and tan setae. *Genitalia*. Abdominal sternum VII with short, pointed mesoventral process. Sternum VIII posteroventral projection bearing small peg-like setae, in ventral view with posterior margin concave. Segment IX anterolateral margin convex, posterolateral margin straight; in dorsal view anterior margin shallowly concave, posterior margin concave. Tergum × with dorsal sclerite continuous with ventral sclerite; ventral sclerite small with rounded emargination on posterior margin; membranous apex extending down to base of subgenital plate. Subgenital plate fused with ventral sclerite of tergum X; with dorsal arm not apparent; ventral arm rounded basally, curved dorsad, apex acute, in ventral view subovate with rounded apical emargination. Inferior appendage with small dorsomesal spine, apex truncate, bearing single dorsal spine; in ventral view with subquadrate basal projection on outer margin, with 2 small apicomesal projections. Phallus apex with 1 dorsal, 1 ventral, and 1 pair of lateral elongate sclerites, apex bulbous.

##### Material examined.

*Holotype male*: **PUERTO RICO:** Maricao Fish Hatchery, 23.xii.1962, P and P Spangler (in alcohol) (USNM 66885) (NMNH). *Nontypes*: **DOMINICAN REPUBLIC: La Estrelleta Province:** 4 km SE Río Limpio,elev. ca. 760 m, 24–25.v.1973, D. and M. Davis, 1 male (NMNH); **La Vega:** Jarabacoa, 1–2 km S, 19°06.9'N, 70°37.0'W, elev. 520 m, 8–21.v.1995, W.N. Mathis, 1 male, 2 females (NMNH); Jarabacoa, 3–4.vi.1969, Flint and Gomez, 1 male (in alcohol) (NMNH); Salto Guasara, 9.5 km W Jarabacoa, elev. 680 m, 10°04.4'N, 70°42.1'W, 9.v.1995, O.S. Flint, Jr., 1 male (NMNH); Río Baiguate, 1–2 km S Jarabacoa, elev. 520 m, 19°06.9'N, 70°37.0'W, 809.v.1995, O.S. Flint, Jr., 3 males, 5 females (NMNH); Jarabacoa, 13.xi.1984, P. and P. Spangler and R. Faitoute, 1 male (in alcohol) (NMNH); **Pedernales:** Pedernales, 21 km N, elev. 270 m, 18°09.3'N, 71°45.6'W, Río Mulito, 18.iii.1999, W.N. Mathis, 1 female (NMNH); **JAMAICA: St. Andrews Parish:** Chestervale, Yallahs River, 24–25.vii.1962, Farr, O., and R. Flint, 1 male, 2 females (NMNH); Chestervale, Yallahs River, 17.vii.1963, Flint and Farr, 5 males, 1 female (NMNH); St. Andrew Parish, Newcastle, M.P.16.5, 18.vii.1963, Flint and Farr, 1 male (NMNH); Yallahs River, Hill Gap, elev 920 m, 18°05.1'N, 76°41.1'W, 26.iv.2000, O.S. Flint, Jr., 3 males (NMNH); Mavis Bank, Yallahs River, 4.3 km SE, 18°1.4'N, 76°38.1'W, elev. 480 m, 22–23.iv.2000, W.N. Mathis, 1 female (NMNH); **PUERTO RICO: Jayuya:** 2 km E Río Saliente, 18°12.8'N, 66°33.9'W, 22.ix.1995, D. and W.N. Mathis, 2 males (NMNH); **Ponce:** Real Anon, at Río Inabon, 18°7'N, 66°34'W, 30.vi.2008, W.E. Steiner, J.M. Swearingen, O.H. Garrido, and A.R. Perez-Asso, at black light in gap of mixed montane rain forest, 3 males (in alcohol) (NMNH).

##### Etymology.

Unknown.

#### Leucotrichia viridis

Taxon classificationAnimaliaTrichopteraHydroptilidae

Flint, 1967

Fig. 45

viridis Flint, 1967: 10 [Type locality: Guatemala, Izabal, Las Escobas near Matias de Galvez; NMNH; male]; 1970: 5 [male, distribution].

##### Diagnosis.

**Figure 45.**
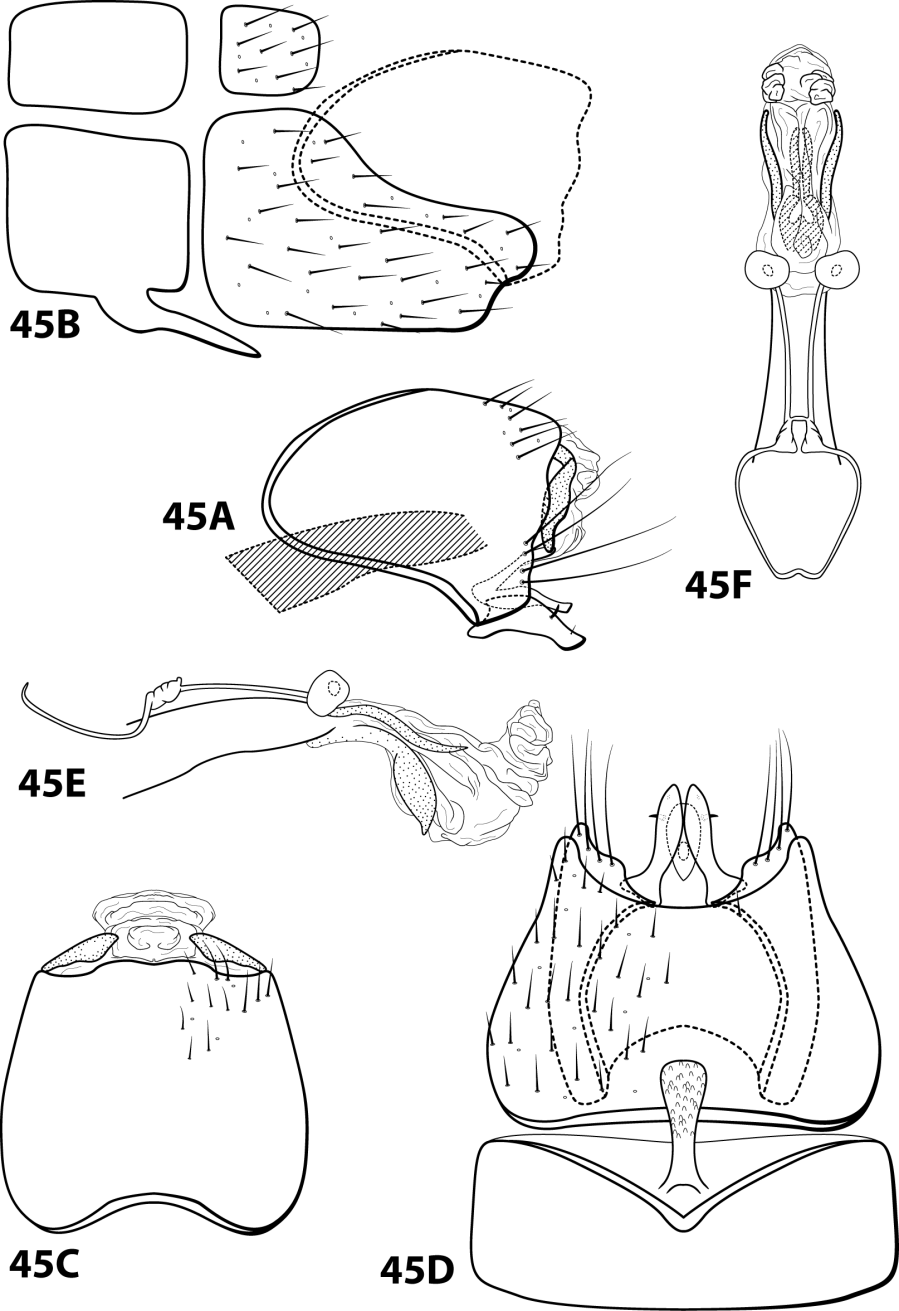
*Leucotrichia viridis* Flint, 1967 (USNM69586). Male genitalia: **A** segments IX–X, lateral (base of phallus crosshatched) **B** segments VII–VIII and segment IX margin, lateral **C** segments IX–X, dorsal **D** segments VII–IX, ventral **E** phallus, lateral **F** phallus, dorsal.

*Leucotrichia viridis* is most similar to *Leucotrichia botosaneanui*, *Leucotrichia chiriquiensis*, *Leucotrichia hispida*, and *Leucotrichia limpia*. These species share a similar combination of characteristics present in the phallus and the posterolateral margin of sternum VIII, as discussed under *Leucotrichia botoseaneanui*. *Leucotrichia viridis* is distinct from the others in having 2 dorsal spines on the inferior appendage (Fig. 45A), while the others bear only a single spine. In ventral view, the mesoventral process of sternum VII is much wider than that of the other species and the apex is much more enlarged (Fig. 45D). The pair of ventral, ovate sclerites on the phallus apex of *Leucotrichia viridis* distinguishes it from the other 4 species (Fig. 45E).

##### Description.

*Male*. Length of forewing 2.3–3.1 mm, 1 individual 1.6 mm (n=68). Head unmodified, with 3 ocelli; antennae unmodified. Dorsum of head brown with light yellow setae; thorax brown with yellow setae dorsally, brown ventrally; leg segments with brown setae. Forewings covered with fine mottled yellow and brown setae, apex with brown setae. *Genitalia*. Abdominal sternum VII with large mesoventral process with apex enlarged, rugose in ventral view. Sternum VIII in ventral view with posterior margin concave. Segment IX anterolateral margin broadly produced dorsolaterally, posterolateral margin irregular; in dorsal view anterior margin concave, posterior margin straight. Tergum × with dorsal sclerite small, simple; ventral sclerite semielliptic with tridentate posterior margin; membranous apex not well developed. Subgenital plate with dorsal arm not apparent; ventral arm arched mesally apex truncate, in ventral view obovate. Inferior appendage broadest basally, arched mesally, bearing 1 large and 1 small dorsal spine; in ventral view with broad mesal constriction. Phallus with median complex bearing elongate basal supports; apex bearing pair of small, membranous lobes and 2 pairs of sclerites: 1st pair dorsolateral and sinuate, 2nd pair ventral and ovate.

##### Material examined.

*Holotype male*: **GUATEMALA: Izabal:** Las Escobas near Matias de Galvez, 14-16.viii.1965, Flint and Ortiz (USNM 69586) (NMNH). *Paratypes*: Same data as holotype, 14 males, 3 females (in alcohol); same data as holotype, 33 males (in alcohol) (NMNH). *Nontypes*: **EL SALVADOR:** Lake Ilopango, 5.viii.1967, O.S. Flint, Jr., 1 female (NMNH); **MEXICO: Puebla:** Patla, 16.iv.1975, J. Bueno-Soria, 1 male, 1 female (in alcohol) (NMNH); **Veracruz:** Fortín de las Flores, 17.v.1964, Blanton et al., 11 males (in alcohol) (NMNH); Veracruz Río, 3 km N Chocaman, 2.v.1981, C.M. and O.S. Flint, Jr., 7 males, 1 female (NMNH); **PANAMA: Chiriqui:** David, Rovira, 13.vii.1964, elev. 2200’, A. Broce, 1 male, 1 female (in alcohol) (NMNH).

##### Etymology.

Unknown.

#### Leucotrichia yungarum

Taxon classificationAnimaliaTrichopteraHydroptilidae

Angrisano & Burgos, 2002

Figs 4E , 46

yungarum Angrisano & Burgos, 2002: 105 [Type locality: Argentina, Salta, Finca Jakúlica, IML; male].

##### Diagnosis.

**Figure 46.**
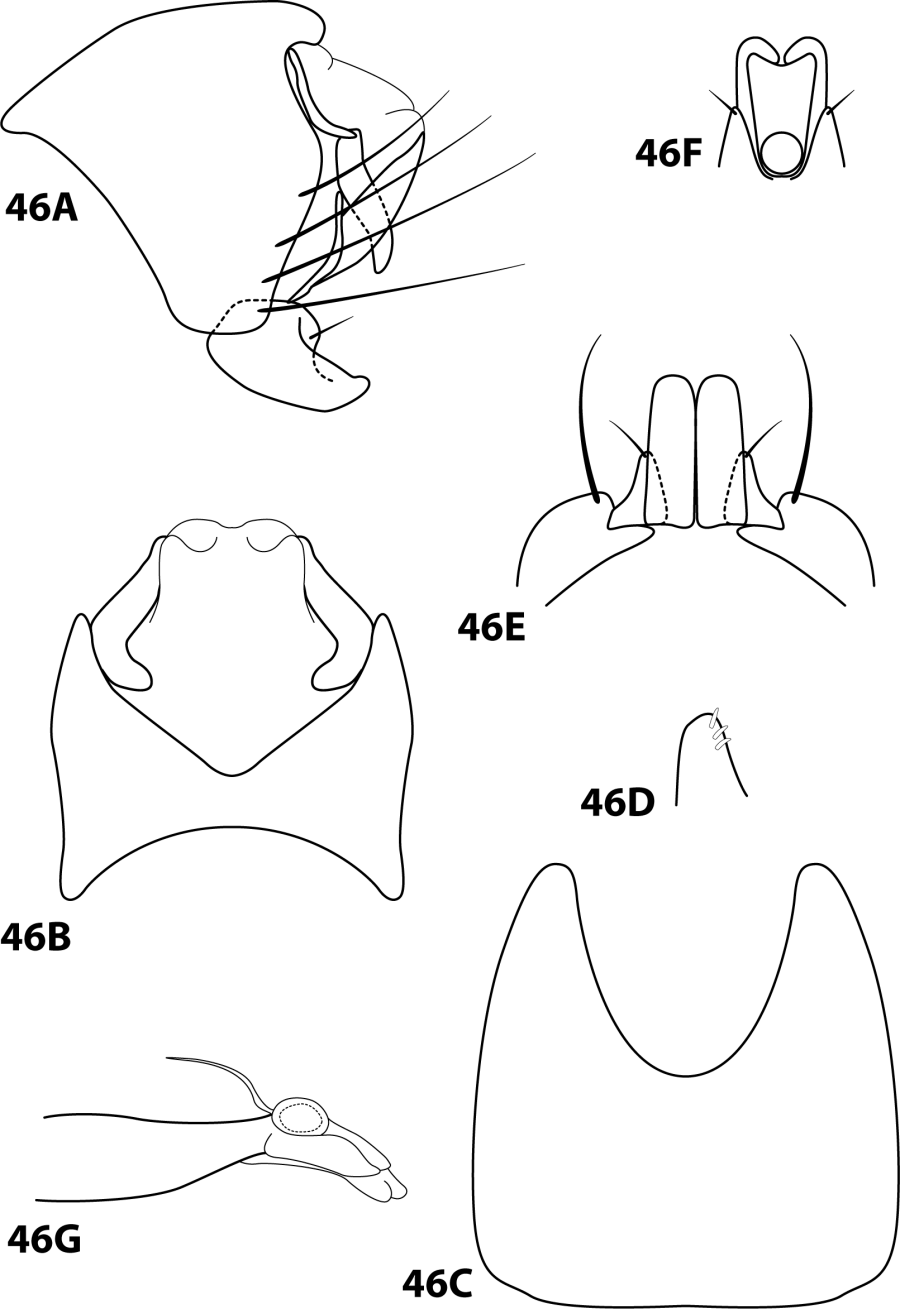
*Leucotrichia yungarum* Angrisano & Burgos, 2002 (redrawn from Angrisano and Burgos 2002). Male genitalia: **A** segments IX–X, lateral **B** segment IX, dorsal **C** segment VIII, ventral **D** apex of segment VIII, dorsal **E** apex of segment IX and inferior appendage, ventral **F** inferior appendage and subgenital plate, dorsal **G** phallus, lateral.

*Leucotrichia yungarum* is similar to *Leucotrichia alisensis*; the 2 species share a similar shape in both the inferior appendage and the ventral arm of the subgenital plate when viewed laterally. In ventral view, the apex of the inferior appendage is digitate in both species. In *Leucotrichia yungarum*, the concave posterior margin of sternum VIII is U-shaped and without any emargination (Fig. 46C), while the same margin in *Leucotrichia alisensis* is V-shaped and with a small mesal emargination. *Leucotrichia yungarum* also differs from *Leucotrichia alisensis* in its lack of anterolateral projection in segment IX. Additionally, *Leucotrichia yungarum* bears an additional pair of posterolateral warts on the head that are not present in *Leucotrichia alisensis* (Fig. 4E).

##### Description.

Redescribed from Angrisano and Burgos (2002). *Male*. Length of forewing 3.0 mm (n=1). Head with 2 pairs of posterolateral warts, with 3 ocelli (only 2 given in illustration); antennae unmodified. *Genitalia*. Abdominal sternum VII with short mesoventral process (not illustrated). Sternum VIII posteroventral projection bearing small peg-like setae, in ventral view with posterior margin concave. Segment IX anterolateral margin produced mesally, posterolateral margin convex; in dorsal view anterior margin concave, posterior margin concave. Tergum × with dorsal sclerite slender, elongate; ventral sclerite simple; membranous apex not well developed. Subgenital plate with dorsal arm not apparent; ventral arm extending dorsad, apex acute. Inferior appendage broadest basally, bearing single dorsal spine; in ventral view with lateral margins parallel. Phallus apex relatively short, without sclerites or spines.

##### Material examined.

*Holotype male*: Holotype deposited at IML, but could not be obtained.

##### Etymology.

Named for the phytogeographical region “Yungas”, cloud forest, of northeast Argentina, where the holotype was collected.

### Key to the males of *Leucotrichia*

All extant species are included. In most cases, it should be possible to identify species by simple visual comparisons to illustrations and by referring to the species diagnoses and descriptions. The following key is intended to help highlight features of the male head and genitalia that are most useful in identifying species and should be used in partnership with the illustrations and descriptions provided.

**Table:** 

1	Ocelli 2	*pictipes* group... **2**
–	Ocelli 3	*melleopicta* group... **5**
2(1)	Head modified, with eversible posterolateral warts (Figs 3A, C, 4D)	**3**
–	Head unmodified	***Leucotrichia imitator***
3(2)	Antennae without modifications; head without further modifications	***Leucotrichia sarita***
–	Antennae with modified basal segments; head with either setiferous protuberance on dorsum or patches of scales on dorsum and on membranous lobes beneath posterolateral warts or with (Fig. 3A, C)	**4**
4(3)	Patches of scales present on dorsum of head and on membranous lobes beneath posterolateral warts; setiferous protuberance on dorsum of head absent (Fig. 3C)	***Leucotrichia pictipes***
–	Patches of scales absent from dorsum of head and from membranous lobes beneath posterolateral warts; setiferous protuberance on dorsum of head present (Fig. 3A)	***Leucotrichia fairchildi***
5(1)	Head with 1 additional pair of posterolateral warts, 2 pairs in total (Fig. 4E)	***Leucotrichia yungarum***
–	Head with 1 pair of posterolateral warts	**6**
6(5)	Antennae with modified basal segments, either broadened or elongated (Figs 3B, 4A, C)	**7**
–	Antennae without modifications	**9**
7(6)	Scape not elongate, following 5–6 antennal segments broadened (Fig. 3B)	***Leucotrichia inflaticornis***
–	Scape elongate, following 5–6 antennal segments not broadened (Fig. 4A, C)	**8**
8(7)	Head with short, black setae anteriorly; without scales (Fig. 4A)	***Leucotrichia chiriquiensis***
–	Head without short, black setae; with patches of scales dorsally (Fig. 4C)	***Leucotrichia procera***
9(6)	Phallus slender and tubular, curving strongly ventrad (Figs 19E, 25E, 38E)	**10**
–	Phallus not slender and tubular or curving strongly ventrad	**12**
10(9)	Phallus apex with a series of membranous ridges, appearing stellate in dorsal view (Fig. 38F)	***Leucotrichia riostoumae***
–	Phallus without series of membranous ridges, not appearing stellate	**11**
11(10)	Inferior appendages fused in ventral view, digitate in lateral view (Figs 25A, D)	***Leucotrichia forrota***
–	Inferior appendages separate in ventral view, basally subquadrate in lateral view (Fig. 19A, D)	***Leucotrichia inops***
12(9)	Phallus median complex with basal supports (see Fig. 10F)	**13**
–	Phallus median complex without basal supports	**18**
13(12)	With small, pointed mesoventral process on abdominal segment VI (Fig. 12D)	***Leucotrichia brochophora***
–	Without mesoventral process on abdominal segment VI	**14**
14(13)	Phallus apex with pair of sinuate sclerites ventrolaterally (Fig. 10E)	***Leucotrichia botosaneanui***
–	Phallus apex without pair of sinuate sclerites ventrolaterally	**15**
15(14)	Mesoventral process on abdominal segment VII short, acute, apex pointed in ventral view (Figs 21D, 30D)	**16**
–	Mesoventral process on abdominal segment VII large, elongate, apex rounded in ventral view (Figs 22D, 45D)	**17**
16(15)	Phallus apex with pair of stout dorsal spines, 2 membranous apical lobes (Fig. 30E, F)	***Leucotrichia limpia***
–	Phallus apex without pair of dorsal spines, 2 membranous dorsal lobes and 1 mesoventral lobe (Fig. 21E, F)	***Leucotrichia gomezi***
17(15)	Posteroventral projection bearing cluster of stout, prominent setae (Fig. 22B)	***Leucotrichia hispida***
–	Posteroventral projection not bearing cluster of stout, prominent setae	***Leucotrichia viridis***
18(12)	Posterior margin projection of sternum VIII bearing prominent or peg-like seta(e) (see Figs 9B, 17B)	**19**
–	Posterior margin projection of sternum VIII without prominent or peg-like setae	**23**
19(18)	Posterior margin projection of sternum VIII bearing cluster of prominent setae (see Figs 9B, 15B)	**20**
–	Posterior margin projection of sternum VIII bearing peg-like seta(e) (Figs 17B, 44B)	**22**
20(19)	Inferior appendages fused in ventral view	***Leucotrichia bicornuta***
–	Inferior appendages separate in ventral view	**1**
21(20)	Sternum VIII posterior margin with small, pointed mesal projection in ventral view; inferior appendage bearing single dorsal spine (Fig. 15A, D)	***Leucotrichia dianeae***
–	Sternum VIII posterior margin with digitate mesal projection in ventral view; inferior appendage bearing 1 large dorsal spine and 2 small setae (Figs 41A, D)	***Leucotrichia tapantia***
22(19)	Phallus apex with pair of apical membranous lobes (Fig. 17F)	***Leucotrichia extraordinaria* Bueno-Soria et al., 2001**
–	Phallus apex bulbous, without apical lobes (Fig. 44F)	***Leucotrichia tubifex***
23(18)	Abdominal sternum VII with both mesoventral process and row of prominent setae; sternum VIII with posterodorsal projection (Figs 33B, D)	***Leucotrichia pectinata***
–	Abdominal sternum VII with only mesoventral process; sternum VIII without posterodorsal projection	**24**
24(23)	Abdominal sternum VII with mesoventral process apex rhomboid in ventral view (Fig. 37D)	***Leucotrichia rhomba***
–	Abdominal sternum VII with mesoventral process apex not rhomboid in ventral view	**25**
25(24)	Segment IX with anterolateral margin with curved ventrolateral projection (Fig. 6A)	***Leucotrichia alisensis***
–	Segment IX with anterolateral margin not with curved ventrolateral projection	**26**
26(25)	Phallus apex bearing a large pair of scissors-like apical sclerites (Fig. 16F)	***Leucotrichia dinamica***
–	Phallus apex not bearing large scissors-like apical sclerites	**27**
27(26)	Inferior appendages partially or entirely fused in ventral view (see Figs 26D, 28D, 42D)	**28**
–	Inferior appendages separate in ventral view	**34**
28(27)	Inferior appendages fused basally in ventral view	**29**
–	Inferior appendages entirely fused in ventral view	**30**
29(28)	Phallus trilobed, with rows of large spines; subgenital plate absent (Fig. 28A, F)	***Leucotrichia laposka***
–	Phallus not trilobed, without large spines; subgenital plate present	***Leucotrichia melleopicta***
30(28)	Inferior appendages with dorsal spines or setae	**31**
–	Inferior appendages without dorsal spine or setae	**33**
31(30)	Inferior appendages bearing single mesodorsal seta and 3 apicodorsal setae (Fig. 36A)	***Leucotrichia repanda***
–	Inferior appendages either bearing less than 3 apicodorsal setae or, if bearing 3 apicodorsal setae, without single mesodorsal seta	**32**
32(31)	Phallus apex with peg-like setae (Fig. 14E, F)	***Leucotrichia denticulata***
–	Phallus apex without peg-like setae	***Leucotrichia termitiformis***
33(30)	Phallus apex trilobed, short spines present on dorsal surface	***Leucotrichia interrupta***
–	Phallus apex not trilobed, short spines not present	***Leucotrichia sidneyi***
34(27)	Phallus apex with pair of apical membranous lobes (see Fig. 11E)	**35**
–	Phallus apex without apical membranous lobes	**37**
35(34)	Inferior appendages with dorsomesal “hump” in lateral view (Figs 27A, 43A)	**36**
–	Inferior appendages without dorsomesal “hump” in lateral view (Fig. 11A)	***Leucotrichia brasiliana***
36(35)	Inferior appendages with mitten-like “thumb” in ventral view (Fig. 43D)	***Leucotrichia tritoven***
–	Inferior appendages without mitten-like “thumb”	***Leucotrichia kateae***
37(34)	Inferior appendages apically hooked (Figs 29A, 32A)	**38**
–	Inferior appendages not apically hooked	**39**
38(37)	Subgenital plate with dorsal arm present (Fig. 32A)	***Leucotrichia padera***
–	Subgenital plate with dorsal arm not apparent (Fig. 29A)	***Leucotrichia lerma***
39(37)	Inferior appendages with dorsal spines or setae	**40**
–	Inferior appendages without dorsal spine or setae	***Leucotrichia angelinae***
40(39)	Inferior appendages with single dorsal spine	**41**
–	Inferior appendages with 3 dorsal setae (Fig. 20A)	***Leucotrichia fulminea***
41(40)	Posterior margin of abdominal segment VIII with ventrolateral projection (Fig. 31B)	***Leucotrichia mutica***
–	Posterior margin of abdominal segment VIII without ventrolateral projection (Fig. 8B)	***Leucotrichia ayura***

## Supplementary Material

XML Treatment for Leucotrichia

XML Treatment for Leucotrichia melleopicta

XML Treatment for Leucotrichia adela

XML Treatment for Leucotrichia alisensis

XML Treatment for Leucotrichia angelinae

XML Treatment for Leucotrichia ayura

XML Treatment for Leucotrichia bicornuta

XML Treatment for Leucotrichia botosaneanui

XML Treatment for Leucotrichia brasiliana

XML Treatment for Leucotrichia brochophora

XML Treatment for Leucotrichia chiriquiensis

XML Treatment for Leucotrichia denticulata

XML Treatment for Leucotrichia dianeae

XML Treatment for Leucotrichia dinamica

XML Treatment for Leucotrichia extraordinaria

XML Treatment for Leucotrichia fairchildi

XML Treatment for Leucotrichia forrota

XML Treatment for Leucotrichia fulminea

XML Treatment for Leucotrichia gomezi

XML Treatment for Leucotrichia hispida

XML Treatment for Leucotrichia imitator

XML Treatment for Leucotrichia inflaticornis

XML Treatment for Leucotrichia inops

XML Treatment for Leucotrichia interrupta

XML Treatment for Leucotrichia kateae

XML Treatment for Leucotrichia laposka

XML Treatment for Leucotrichia lerma

XML Treatment for Leucotrichia limpia

XML Treatment for Leucotrichia mutica

XML Treatment for Leucotrichia padera

XML Treatment for Leucotrichia pectinata

XML Treatment for Leucotrichia pictipes

XML Treatment for Leucotrichia procera

XML Treatment for Leucotrichia repanda

XML Treatment for Leucotrichia rhomba

XML Treatment for Leucotrichia riostoumae

XML Treatment for Leucotrichia sarita

XML Treatment for Leucotrichia sidneyi

XML Treatment for Leucotrichia tapantia

XML Treatment for Leucotrichia termitiformis

XML Treatment for Leucotrichia tritoven

XML Treatment for Leucotrichia tubifex

XML Treatment for Leucotrichia viridis

XML Treatment for Leucotrichia yungarum

## Figures and Tables

**Figure 5.**
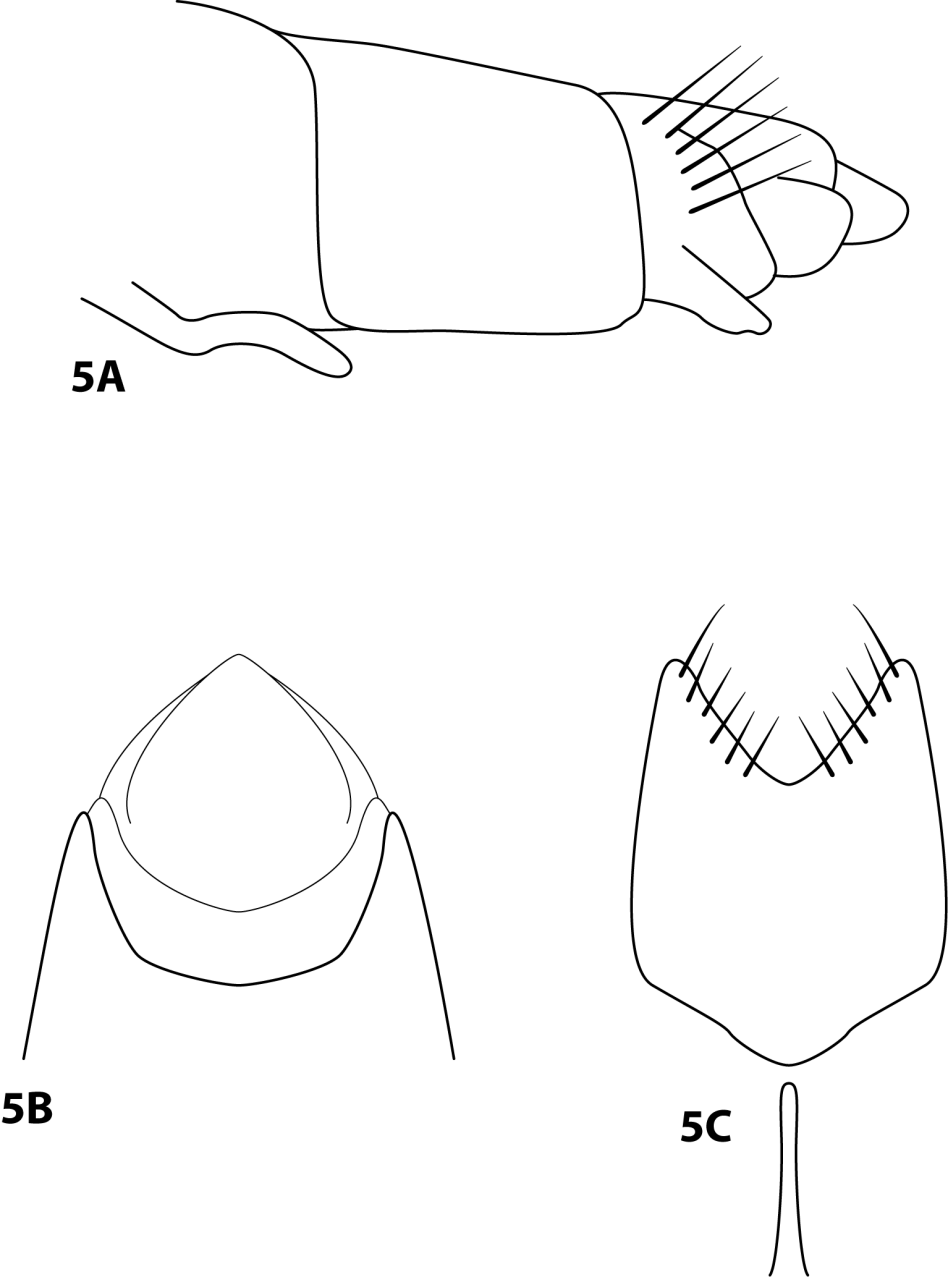
*Leucotrichia adela* Wells & Wichard, 1989 (redrawn from Wells and Wichard 1989). Male genitalia: **A** segments VII–X, lateral **B** segments IX–X, dorsal **C** mesoventral process of segment VII and segment VIII, ventral.

**Figure 6.**
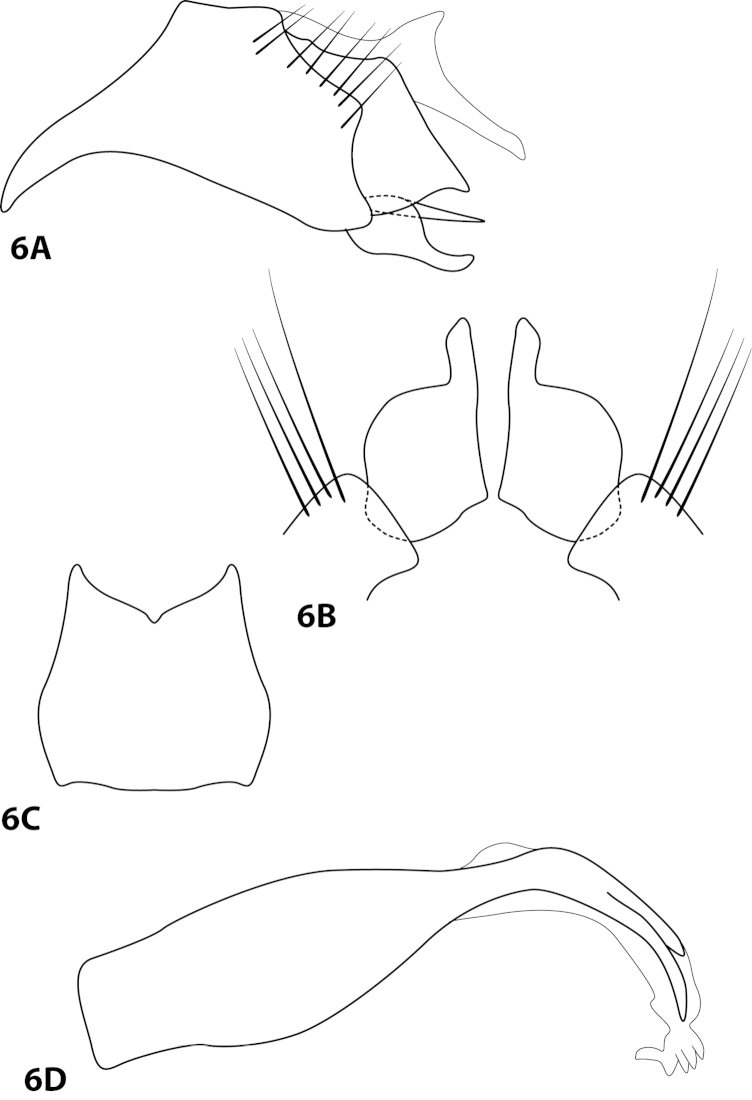
*Leucotrichia alisensis* Rueda Martín, 2011 (redrawn from Rueda Martín 2011). Male genitalia: **A** segments IX–X, lateral **B** apex of segment IX and inferior appendage, ventral **C** segment VIII, ventral **D** phallus, lateral.

**Figure 13.**
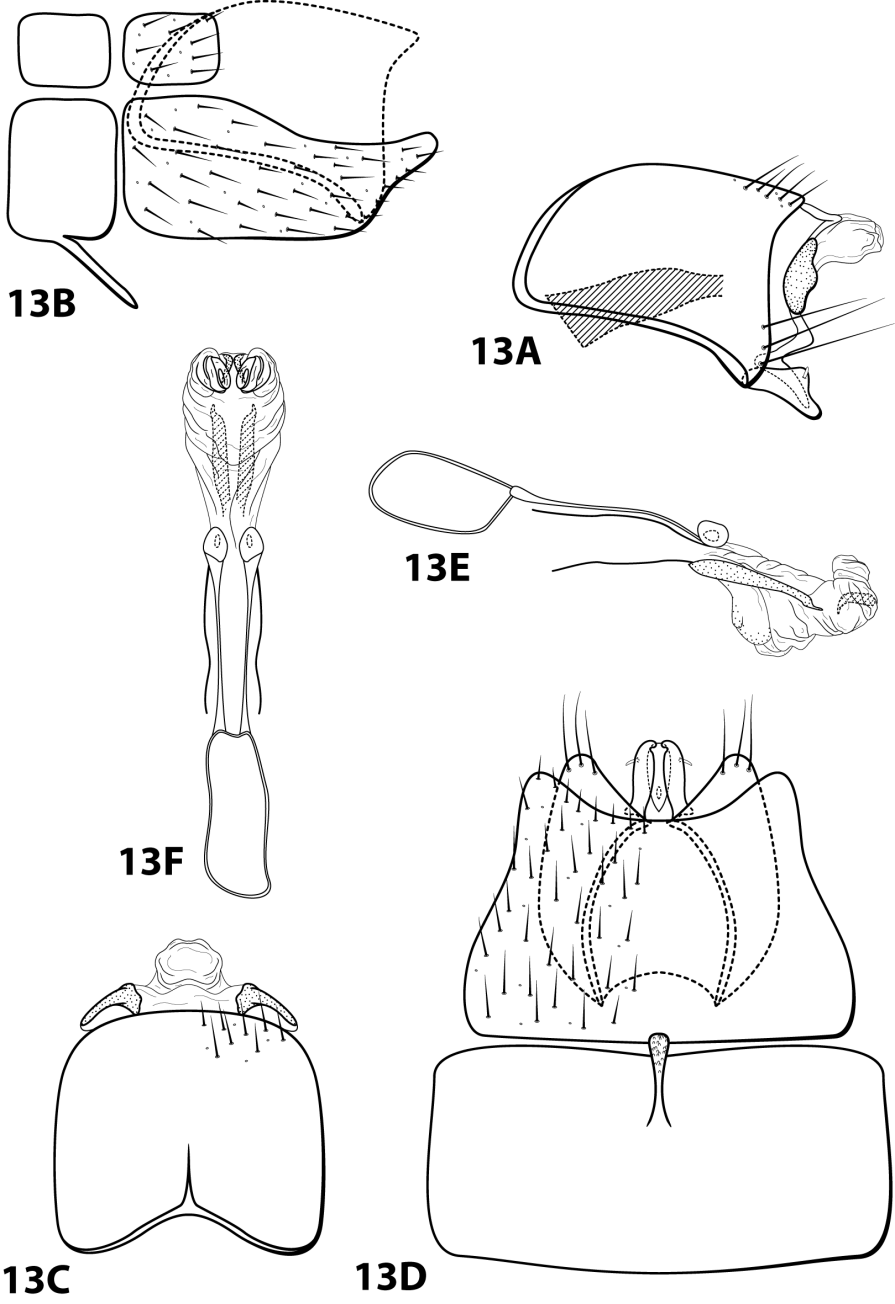
*Leucotrichia chiriquiensis* Flint, 1970 (USNM70896). Male genitalia: **A** segments IX–X, lateral (base of phallus crosshatched) **B** segments VII–VIII and segment IX margin, lateral **C** segments IX–X, dorsal **D** segments VII–IX, ventral **E** phallus, lateral **F** phallus, dorsal.

**Figure 17.**
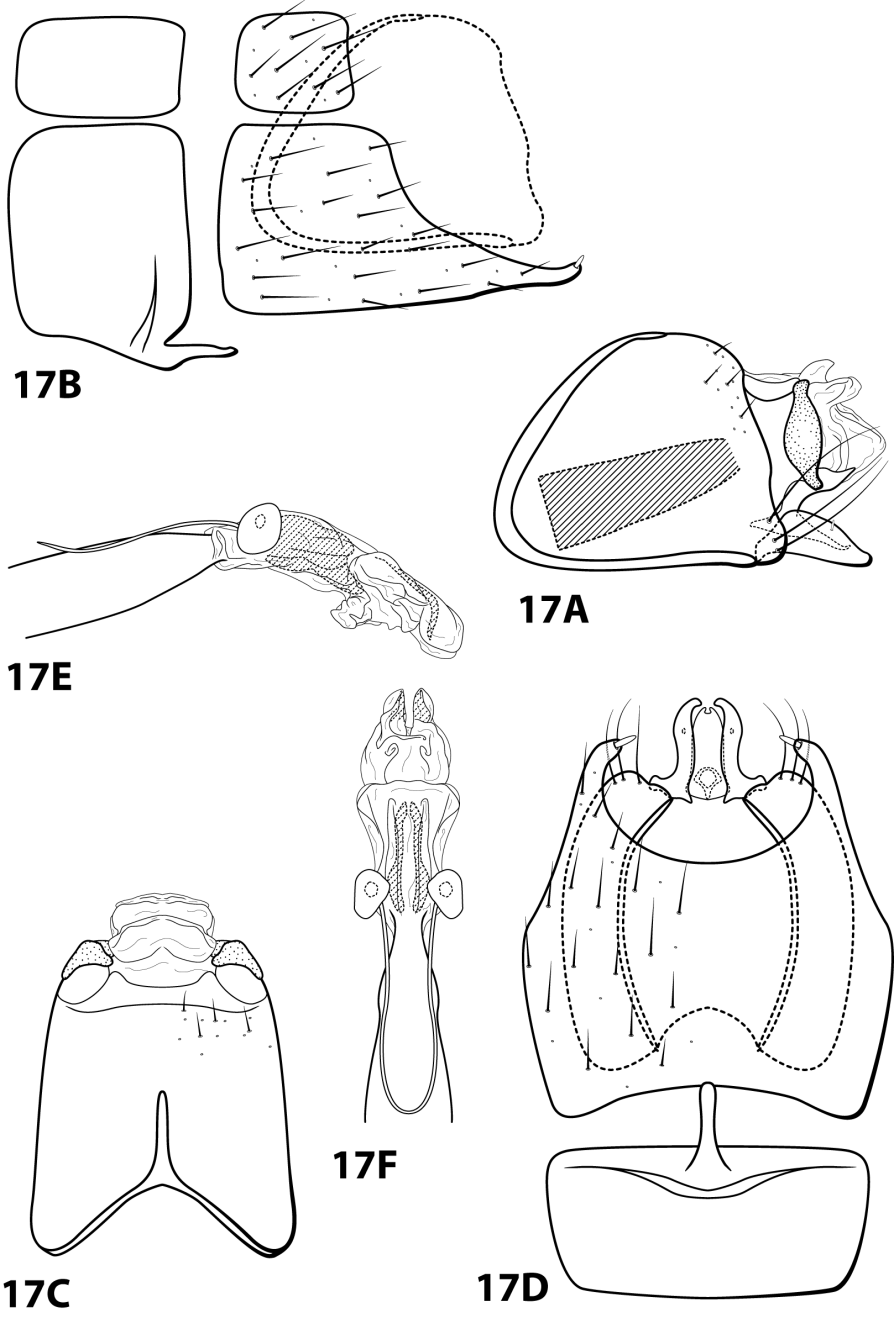
*Leucotrichia extraordinaria* Bueno-Soria, Santiago-Fragoso & Barba-Álvarez, 2001 (UMSP000140695). Male genitalia: **A** segments IX–X, lateral (base of phallus crosshatched) **B** segments VII–VIII and segment IX margin, lateral **C** segments IX–X, dorsal **D** segments VII–IX, ventral **E** phallus, lateral **F** phallus, dorsal.

**Figure 19.**
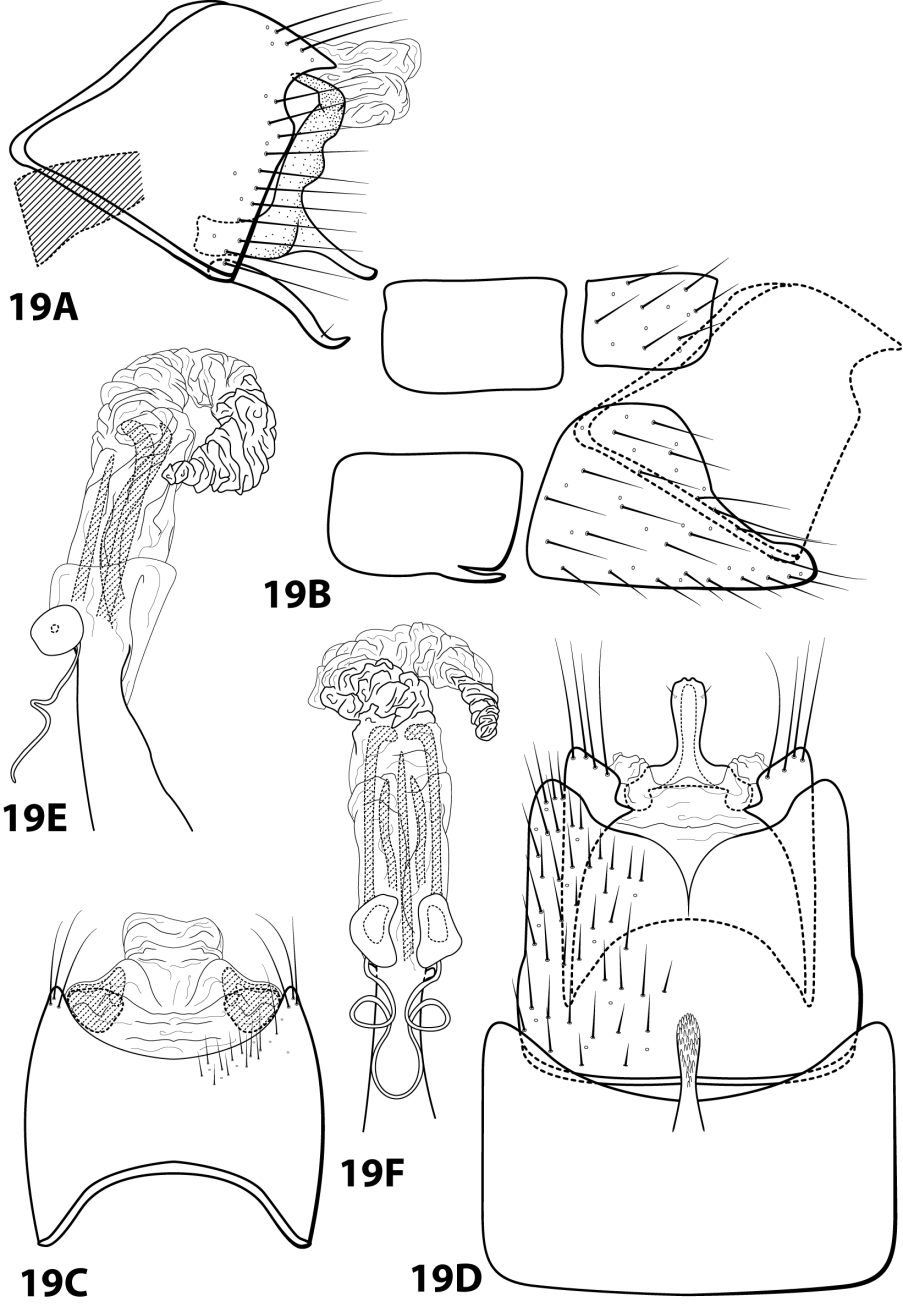
*Leucotrichia forrota* Oláh & Johanson, 2011 (NHRSKAJO000000329). Male genitalia: **A** segments IX–X, lateral (base of phallus crosshatched) **B** segments VII–VIII and segment IX margin, lateral **C** segments IX–X, dorsal **D** segments VII–IX, ventral **E** phallus, lateral **F** phallus, dorsal.

**Figure 20.**
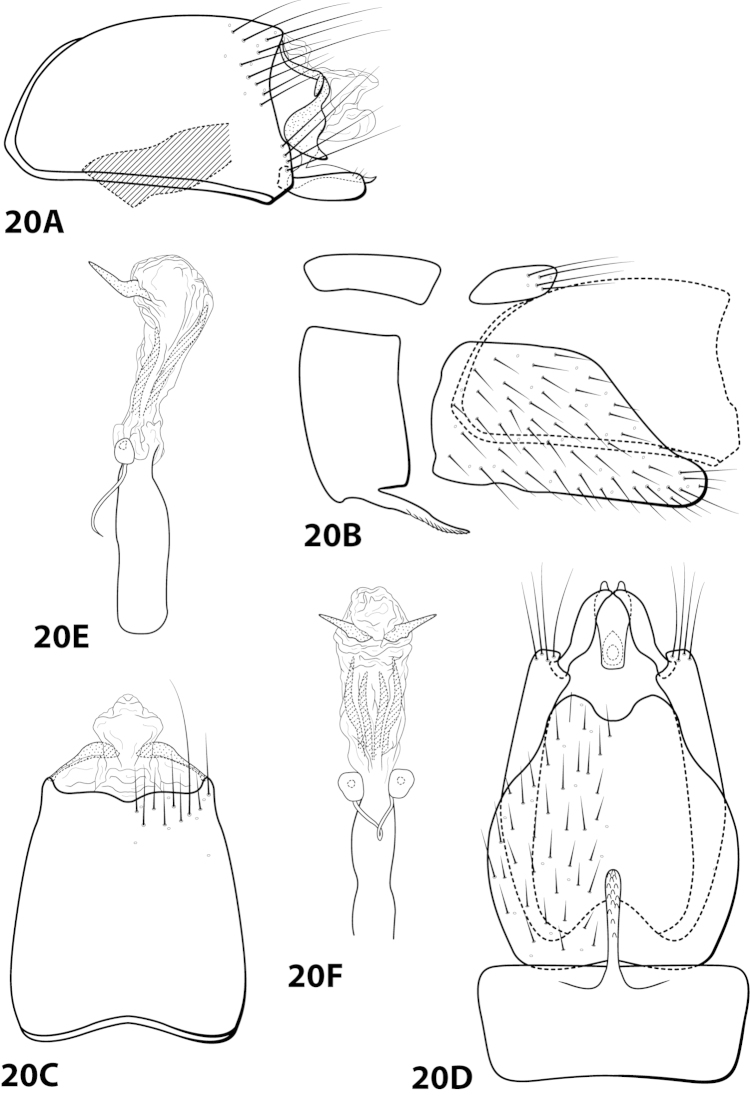
*Leucotrichia fulminea* sp. n. (UMSP000140611). Male genitalia: **A** segments IX–X, lateral (base of phallus crosshatched) **B** segments VII–VIII and segment IX margin, lateral **C** segments IX–X, dorsal **D** segments VII–IX, ventral **E** phallus, lateral **F** phallus, dorsal.

**Figure 24.**
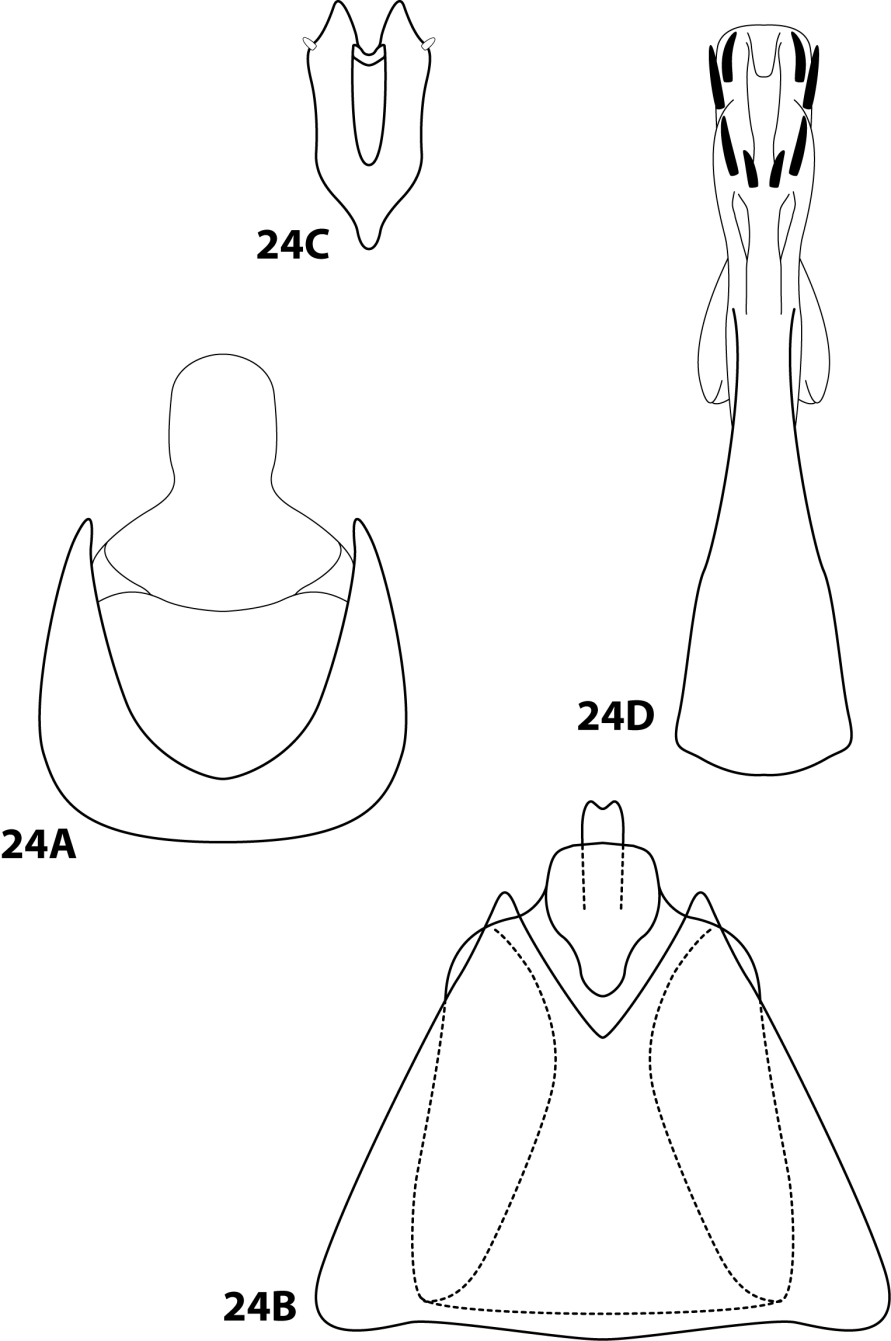
*Leucotrichia inflaticornis* Botosaneanu, 1993 (redrawn from Botosaneanu and Alkins-Koo 1993). Male genitalia: **A** segment IX, dorsal **B** segments VIII–IX, ventral **C** subgenital plate, not to scale, view not specified **D** phallus, ventral.

**Figure 25.**
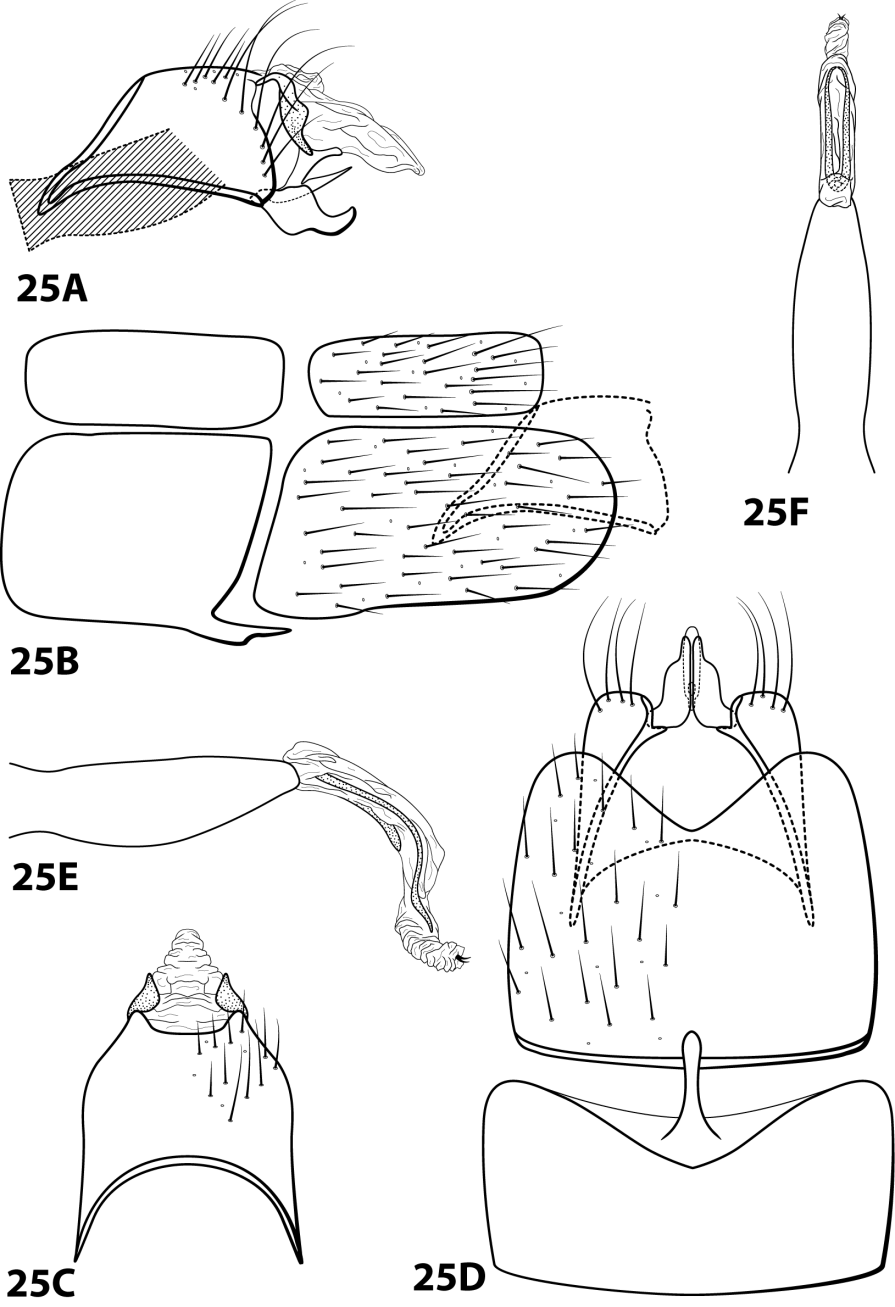
*Leucotrichia inops* Flint, 1991 (USNM104530). Male genitalia: **A** segments IX–X, lateral (base of phallus crosshatched) **B** segments VII–VIII and segment IX margin, lateral **C** segments IX–X, dorsal **D** segments VII–IX, ventral **E** phallus, lateral **F** phallus, dorsal.

**Figure 27.**
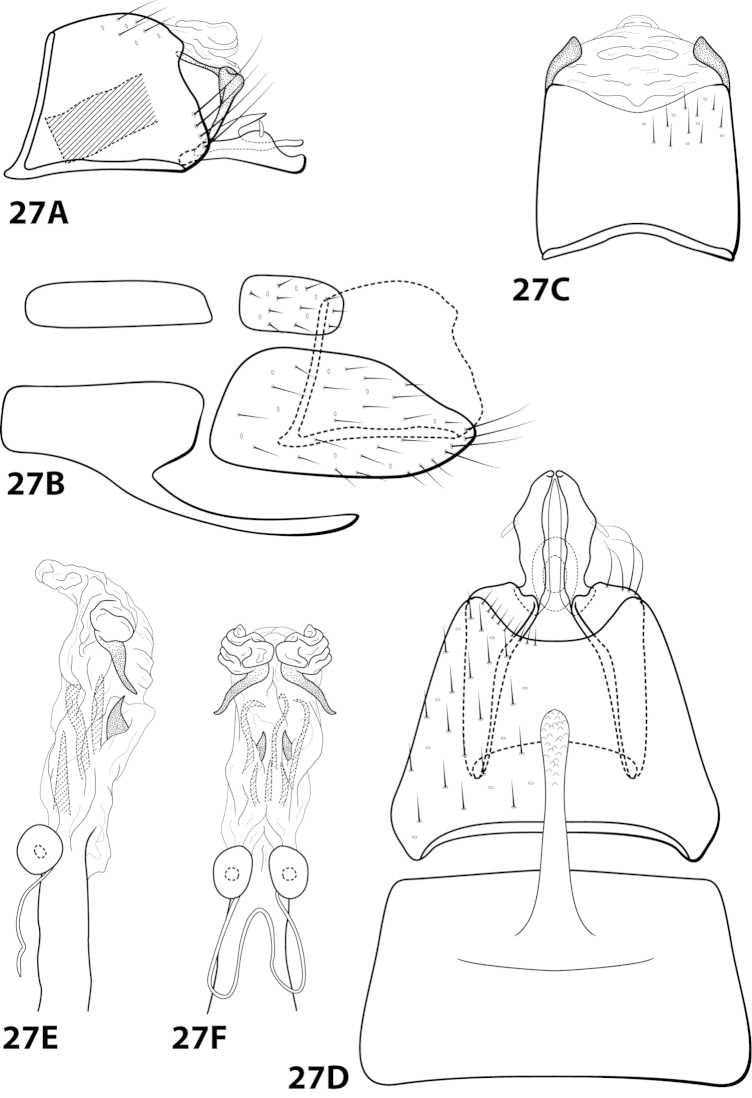
*Leucotrichia kateae* sp. n. (UMSP000201690). Male genitalia: **A** segments IX–X, lateral (base of phallus crosshatched) **B** segments VII–VIII and segment IX margin, lateral **C** segments IX–X, dorsal **D** segments VII–IX, ventral **E** phallus, lateral **F** phallus, dorsal.

**Figure 28.**
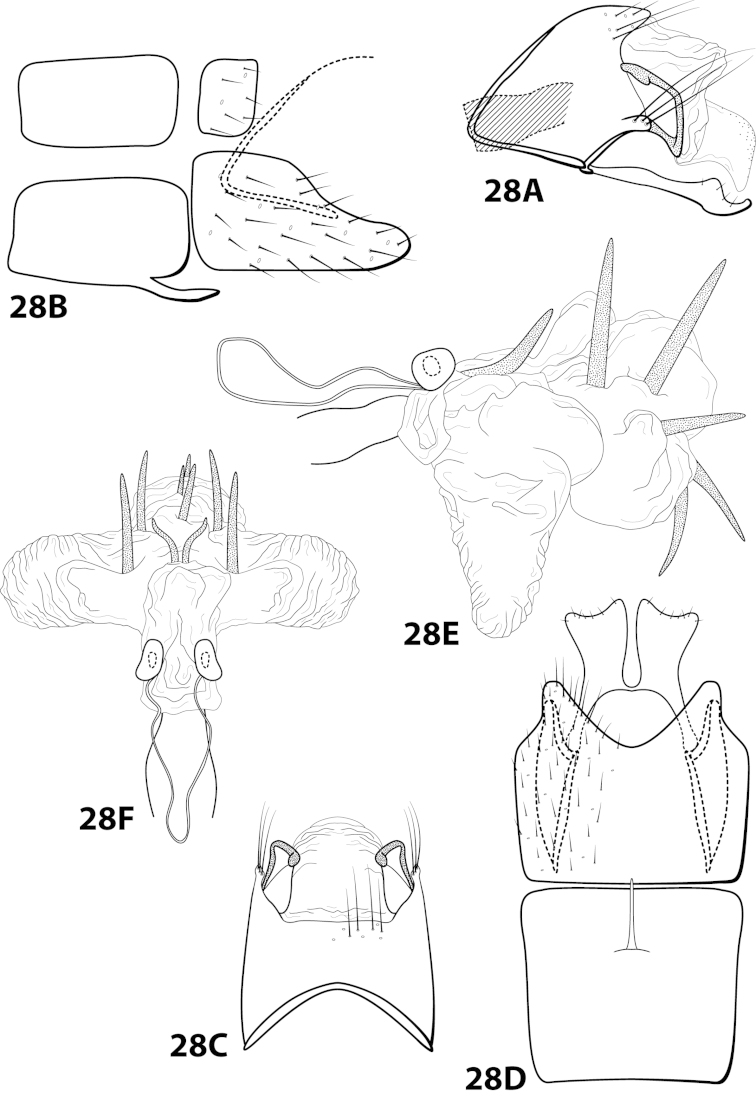
*Leucotrichia laposka* Oláh & Johanson, 2011 (NHRSKAJO000000328). Male genitalia: **A** segments IX–X, lateral (base of phallus crosshatched) **B** segments VII–VIII and segment IX margin, lateral **C** segments IX–X, dorsal **D** segments VII–IX, ventral **E** phallus, lateral **F** phallus, dorsal.

**Figure 29.**
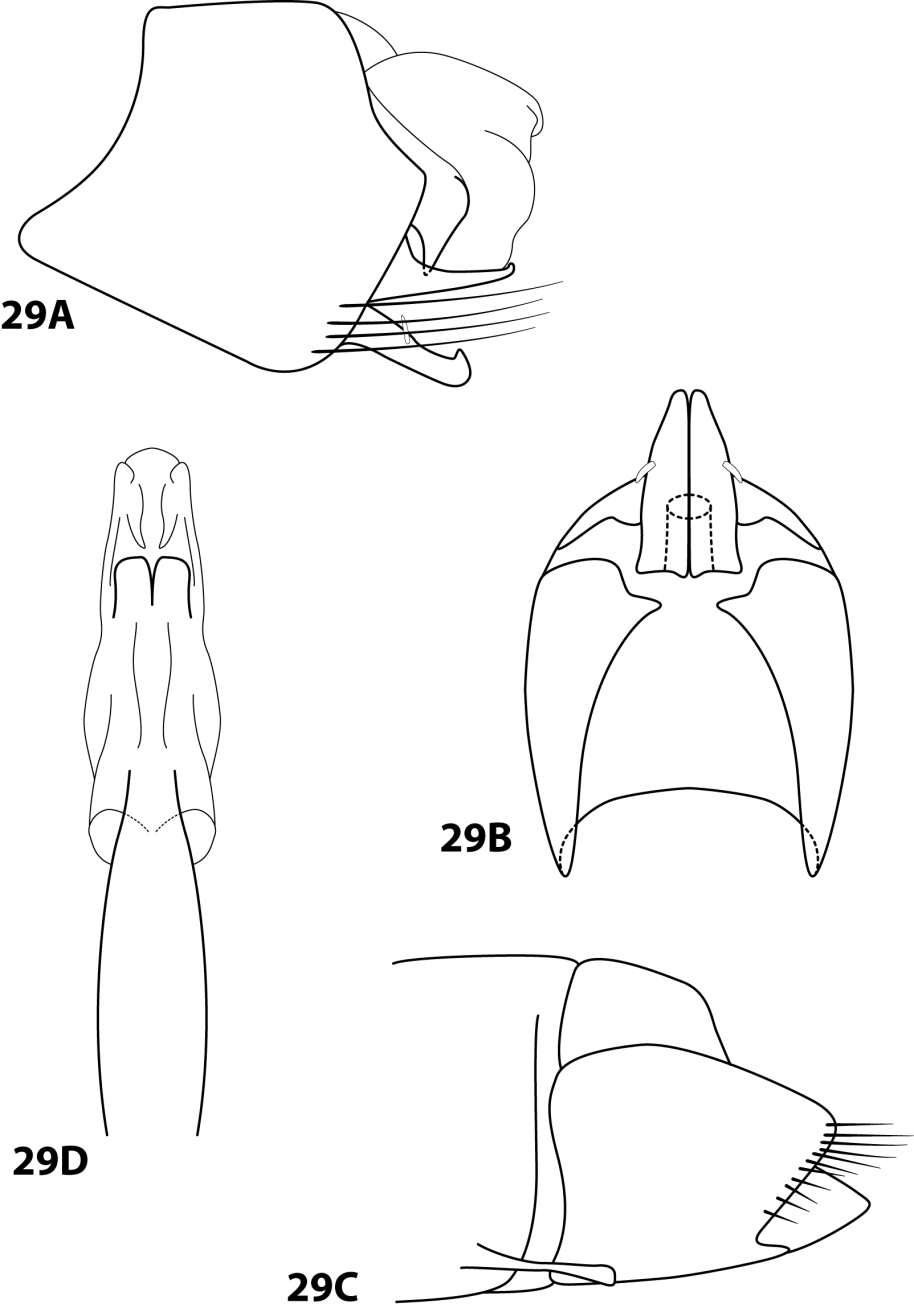
*Leucotrichia lerma* Angrisano & Burgos, 2002 (redrawn from Angrisano and Burgos 2002). Male genitalia: **A** segments IX–X, lateral **B** segment IX, ventral **C** segment VII–VIII, oblique **D** phallus, view not specified.

**Figure 31.**
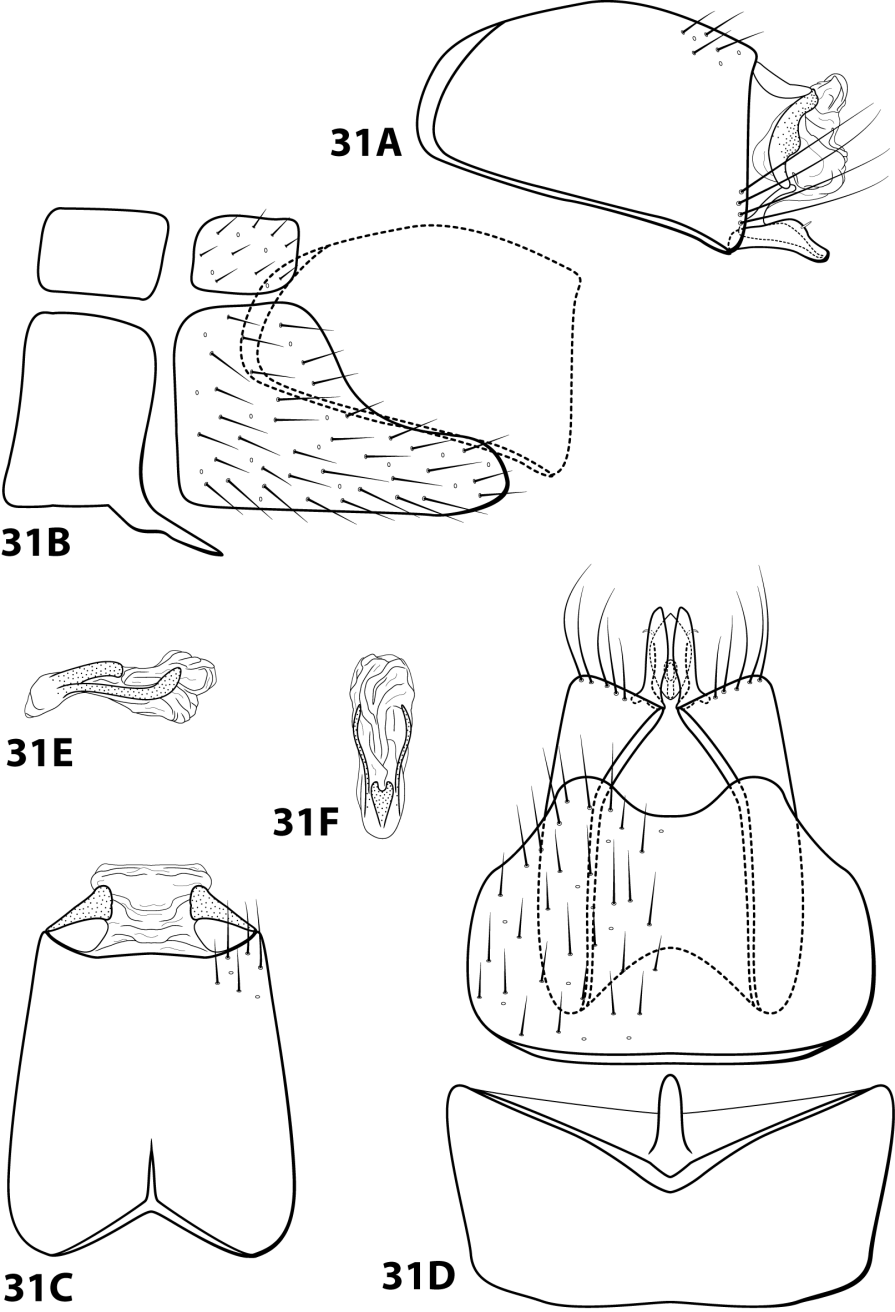
*Leucotrichia mutica* Flint, 1991 (USNM04525). Male genitalia: **A** segments IX–X, lateral (base of phallus crosshatched) **B** segments VII–VIII and segment IX margin, lateral **C** segments IX–X, dorsal **D** segments VII–IX, ventral **E** phallus, lateral **F** phallus, dorsal.

**Figure 36.**
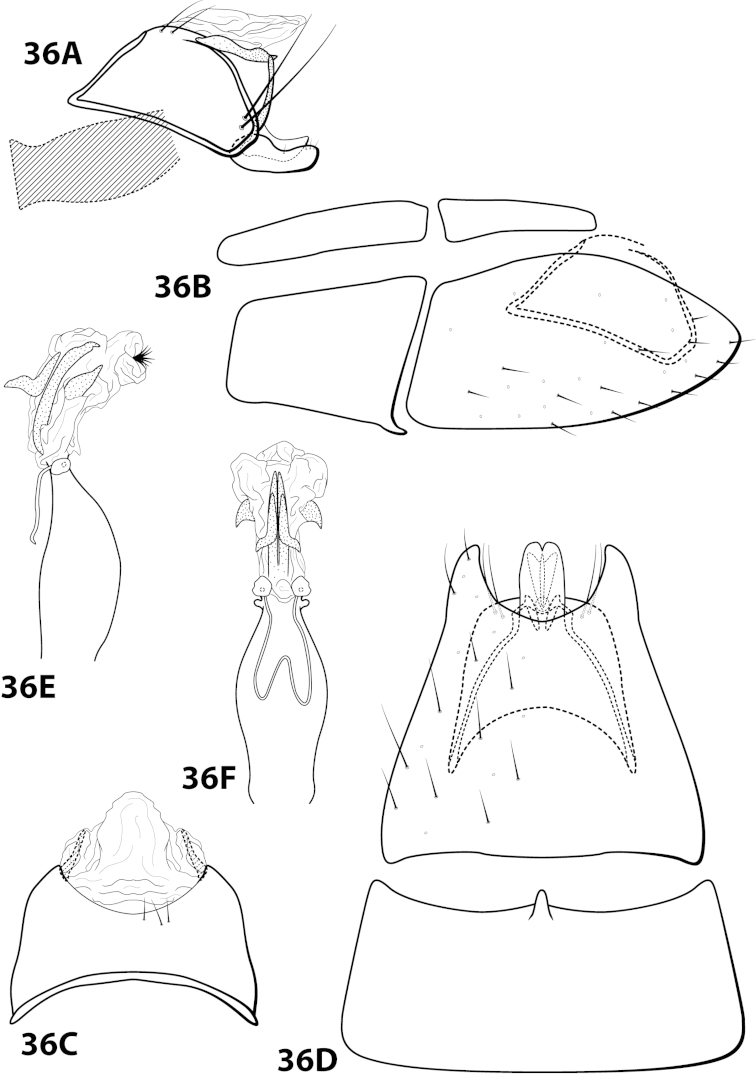
*Leucotrichia repanda* sp. n. (UMSP000201685). Male genitalia: **A** segments IX–X, lateral (base of phallus crosshatched) **B** segments VII–VIII and segment IX margin, lateral **C** segments IX–X, dorsal **D** segments VII–IX, ventral **E** phallus, lateral **F** phallus, dorsal.

**Figure 38.**
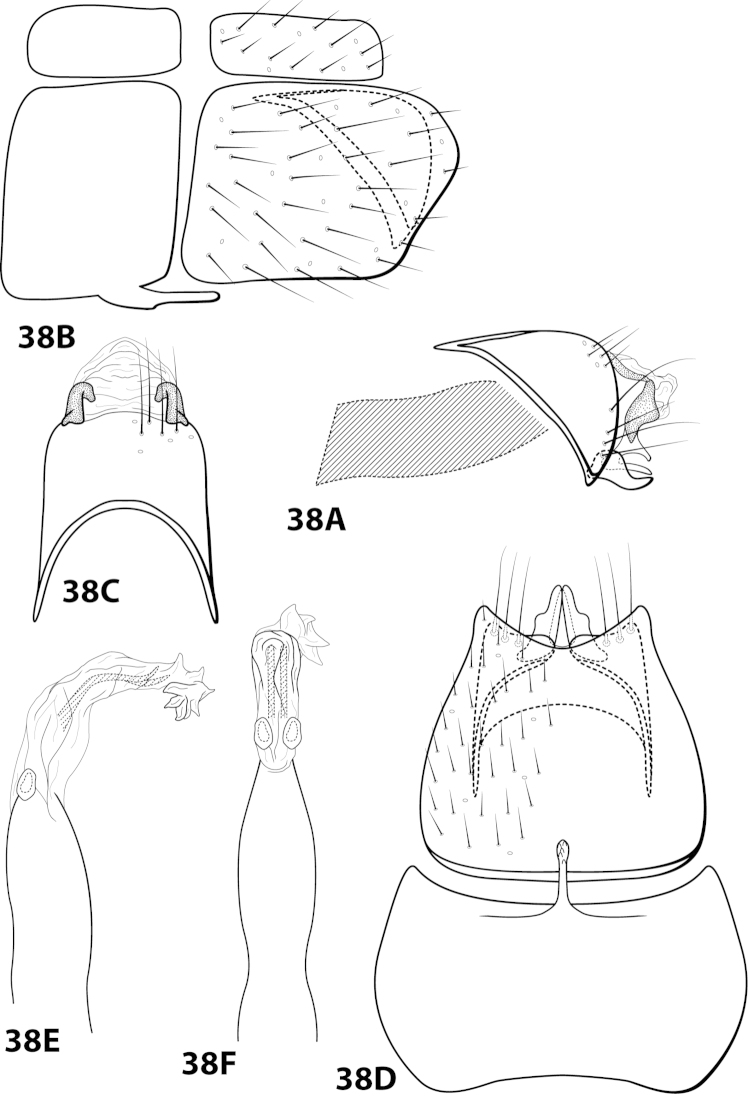
*Leucotrichia riostoumae* sp. n. (UMSP000140832). Male genitalia: **A** segments IX–X, lateral (base of phallus crosshatched) **B** segments VII–VIII and segment IX margin, lateral **C** segments IX–X, dorsal **D** segments VII–IX, ventral **E** phallus, lateral **F** phallus, dorsal.

**Figure 40.**
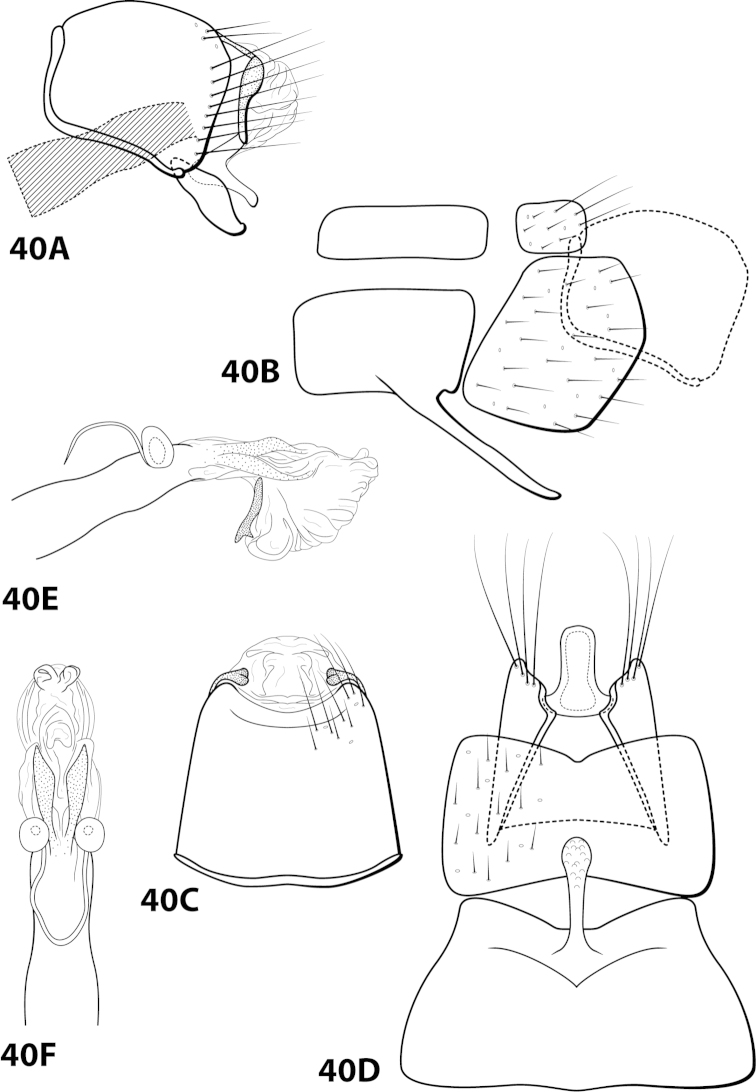
*Leucotrichia sidneyi* sp. n. (UMSP000140465). Male genitalia: **A** segments IX–X, lateral (base of phallus crosshatched) **B** segments VII–VIII and segment IX margin, lateral **C** segments IX–X, dorsal **D** segments VII–IX, ventral **E** phallus, lateral **F** phallus, dorsal.

**Figure 42.**
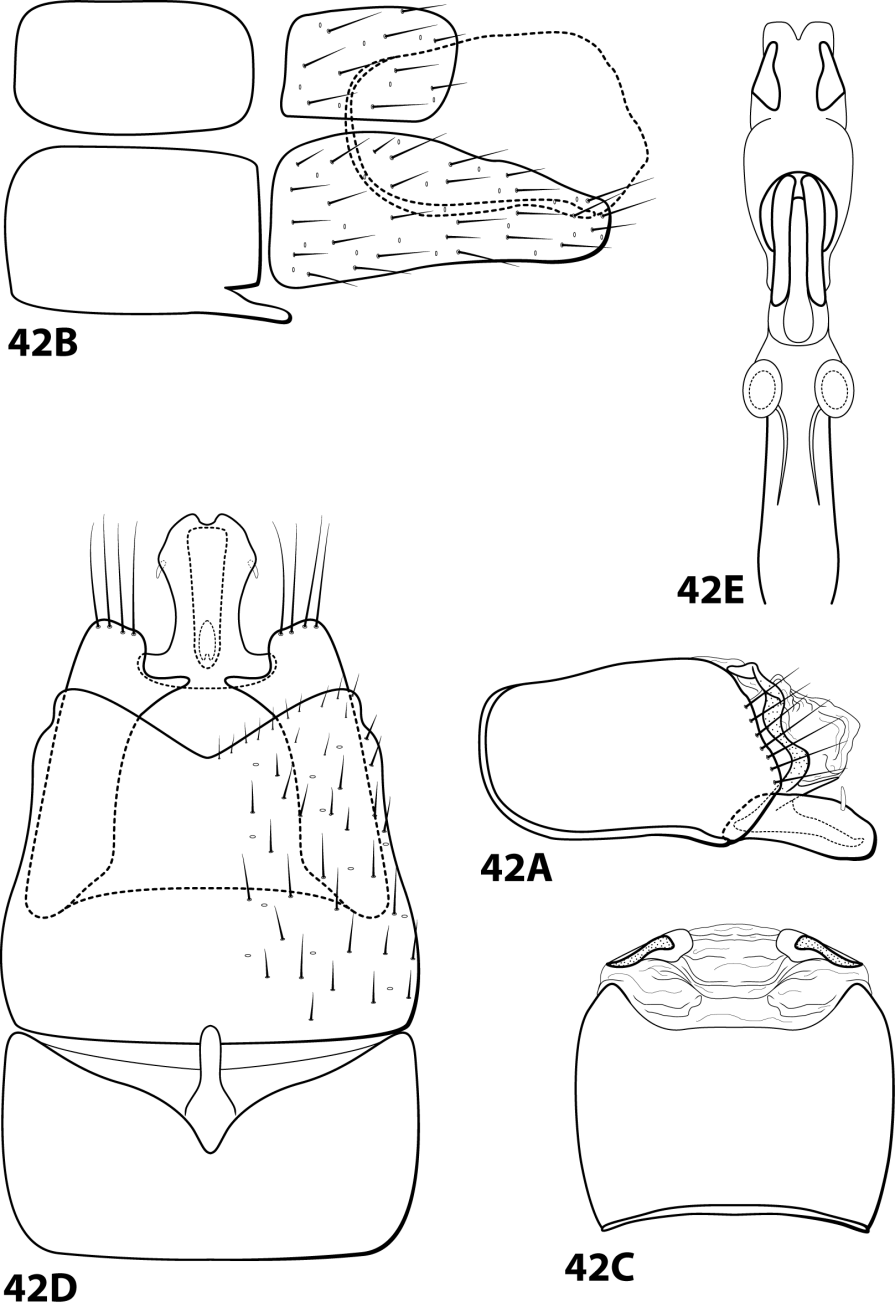
*Leucotrichia termitiformis* Botosaneanu, 1993 (UMSP000140326). Male genitalia: **A** segments IX–X, lateral **B** segments VII–VIII and segment IX margin, lateral **C** segments IX–X, dorsal **D** segments VII–IX, ventral **E** phallus, dorsal, redrawn from original illustration.

**Figure 44.**
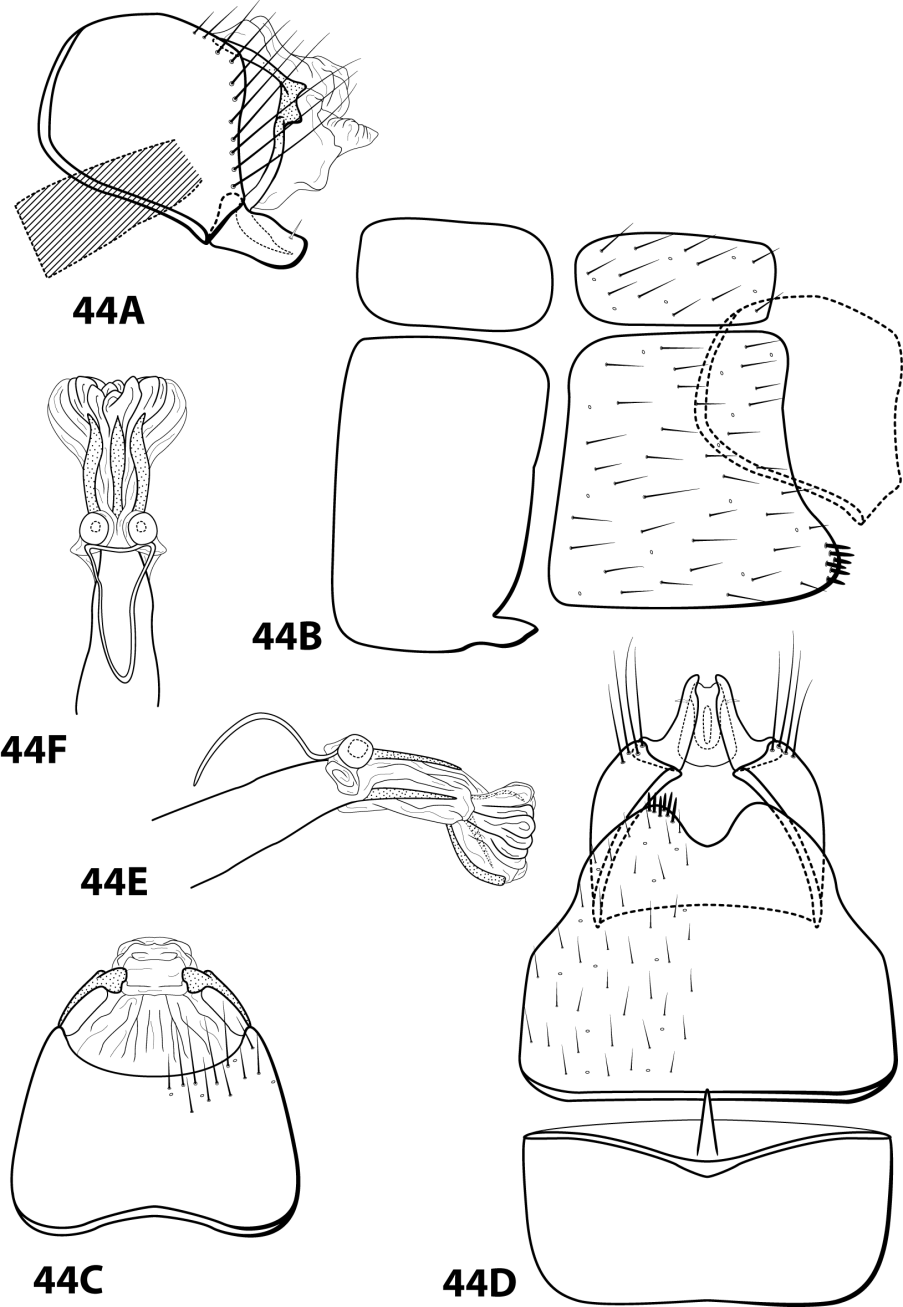
*Leucotrichia tubifex* Flint, 1964 (USNM66885). Male genitalia: **A** segments IX–X, lateral (base of phallus crosshatched) **B** segments VII–VIII and segment IX margin, lateral **C** segments IX–X, dorsal **D** segments VII–IX, ventral **E** phallus, lateral **F** phallus, dorsal.
